# Supplementary material for: Cholesterol removal improves performance of a model biomimetic system to co-deliver a photothermal agent and a STING agonist for cancer immunotherapy
Source: Nat Commun. 2023 Aug 22;14:5111. doi: 10.1038/s41467-023-40814-9 (PMC10444796; doi:10.1038/s41467-023-40814-9)
Supplement: Supplementary file 1 — Supplementary Information [file 41467_2023_40814_MOESM1_ESM.pdf]

Supplementary Information

Cholesterol removal improves performance of a model biomimetic system to co-deliver a photothermal agent and a STING agonist for cancer immunotherapy

Lin Li[1], Mengxing Zhang[2], Jing Li[1], Tiantian Liu[2], Qixue Bao[1], Xi Li[1], Jiaying Long[3], Leyao Fu[1], Zhirong Zhang[3], Shiqi Huang[2], Zhenmi Liu[1,*], Ling Zhang[2,1,*]

[1] Institute of Systems Epidemiology, West China School of Public Health and West China Fourth Hospital, Sichuan University, Chengdu 610041, China.

[2] Med-X center for Materials, College of Polymer Science and Engineering, Sichuan University, Chengdu 610065, China.

[3] Key Laboratory of Drug Targeting and Drug Delivery Systems of Ministry of Education, West China School of Pharmacy, Sichuan University, Chengdu 610065, China.

***Corresponding author**

Prof. Ling Zhang

E-mail: zhangling83@scu.edu.cn

Prof. Zhenmi Liu

E-mail: liuzhenmi1983@hotmail.com

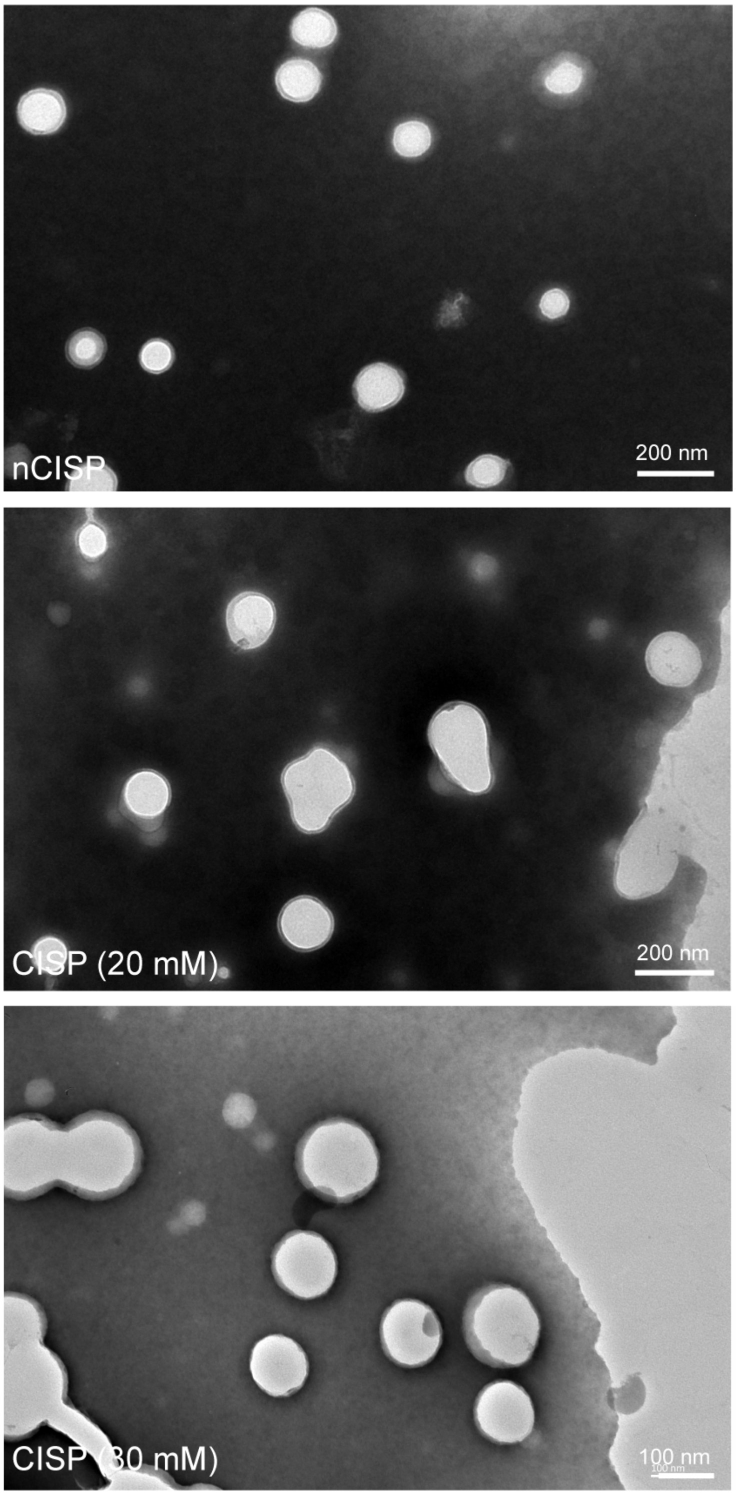

Supplementary Figure 1. Representative transmission electron microscope (TEM) images of nCISP, CISP (20 mM) and CISP (30 mM). *n* = 3 independent samples. The experiments were repeated three times independently with similar results. QFN, Quercetin-ferrum nanoparticles; QFN_{717}, QFN loaded with SR-717; nCISP, normal-Cholesterol cell membrane coated ICB agent and QFN_{717}; CISP (20 mM), low-Cholesterol membrane (treated with 20 mM β-CD) coated ICB agent and QFN_{717}; CISP (30 mM), low-Cholesterol membrane (treated with 30 mM β-CD) coated ICB agent and QFN_{717}.

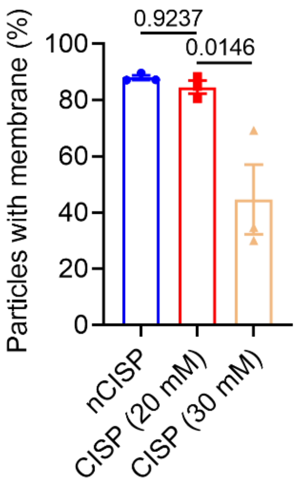

Supplementary Figure 2. The proportion of nanoparticles coated with cell membrane detected by TEM. Data represent mean ± SEM, *n* = 3 independent samples. Statistical significance was determined by one-way ANOVA test, and it was two-sided and adjustments were made for multiple comparisons. The experiments were repeated three times independently with similar results. Source data are provided as a Source Data file. QFN, Quercetin-ferrum nanoparticles; QFN_{717}, QFN loaded with SR-717; nCISP, normal-Cholesterol cell membrane coated ICB agent and QFN_{717}; CISP (20 mM), low-Cholesterol membrane (treated with 20 mM β-CD) coated ICB agent and QFN_{717}; CISP (30 mM), low-Cholesterol membrane (treated with 30 mM β-CD) coated ICB agent and QFN_{717}.

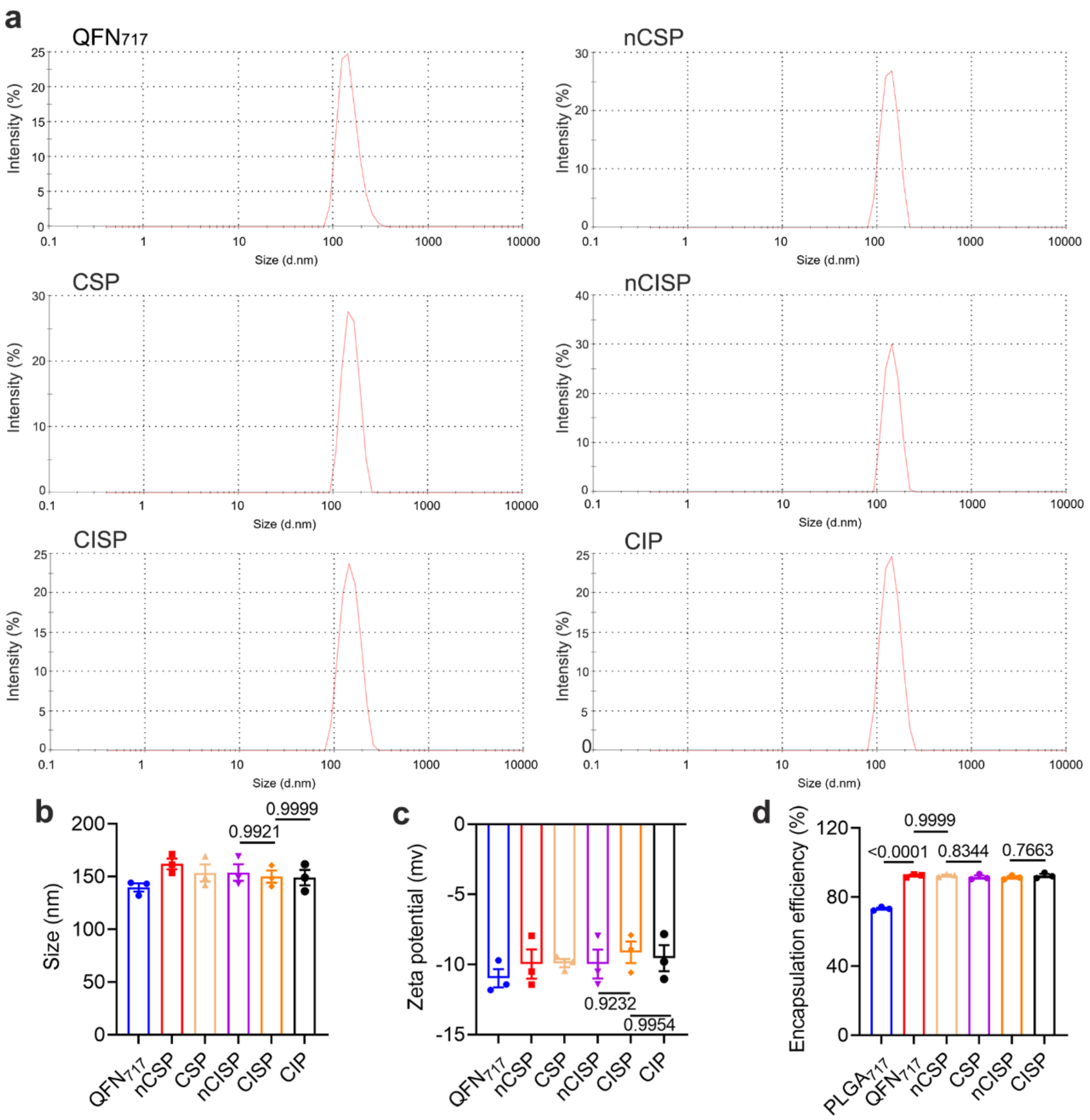

Supplementary Figure 3. Characterization of nanoparticles. a, **b** Sizes of QFN_{717}, nCSP, CSP, nCISP, CISP and CIP measured by Malvern Zetasizer Nano ZS90 dynamic light scattering (DLS) analyzer (Malvern Instrument Ltd, UK). Data represent mean ± SEM, $n = 3$ independent samples. **c** Zeta potential of QFN_{717}, nCSP, CSP, nCISP, CISP and CIP measured by Malvern Zetasizer Nano ZS90 dynamic light scattering (DLS) analyzer. Data represent mean ± SEM, $n = 3$ independent samples. **d** The encapsulation efficiency (EE%) of SR-717 in $PLGA_{717}$ QFN_{717}, nCSP, CSP, nCISP and CISP. Data represent mean ± SEM, $n = 3$ independent samples. Statistical significance was determined by one-way ANOVA test, and it was two-sided and adjustments were made for multiple comparisons (**b**, **c**, **d**). The experiments for **b**, **c** were repeated three times independently with similar results. Source data are provided as a Source Data file. QFN, Quercetin-ferrum nanoparticles; QFN_{717}, QFN loaded with SR-717; $PLGA_{717}$, PLGA nanoparticles loaded with SR-717; nCSP, normal-Cholesterol cell membrane coated QFN_{717}; CSP, low-Cholesterol membrane coated QFN_{717}; nCISP, normal-Cholesterol cell membrane coated ICB agent and QFN_{717}; CISP, low-Cholesterol membrane coated ICB agent and QFN_{717}; CIP, low-Cholesterol membrane coated ICB agent and QFN.

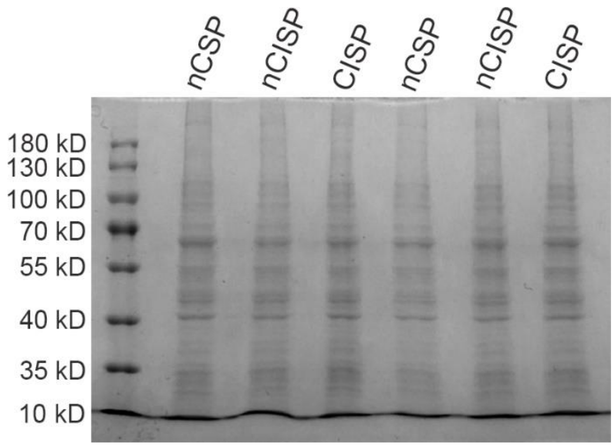

Supplementary Figure 4. Coomassie brilliant blue staining of SDS-PAGE. nCSP, nCISP and CISP with 20 μg proteins were loaded into the SDS-PAGE. The experiments were repeated twice independently with similar results. Source data are provided as a Source Data file. QFN, Quercetin-ferrum nanoparticles; QFN_{717}, QFN loaded with SR-717; nCSP, normal-Cholesterol cell membrane coated QFN_{717}; nCISP, normal-Cholesterol cell membrane coated ICB agent and QFN_{717}; CISP, low-Cholesterol membrane coated ICB agent and QFN_{717}.

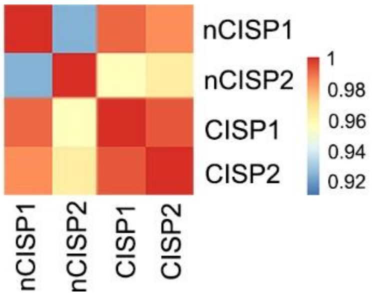

Supplementary Figure 5. Protein expression difference in nCISP and CISP. Label free quantitative proteomics technology was used to study the protein expression nCISP and CISP. *n* = 2 independent samples. Source data are provided as a Source Data file. $PLGA_{717}$, PLGA nanoparticles loaded with SR-717; nCISP, normal-Cholesterol cell membrane coated ICB agent and QFN_{717}; CISP, low-Cholesterol membrane coated ICB agent and QFN_{717}.

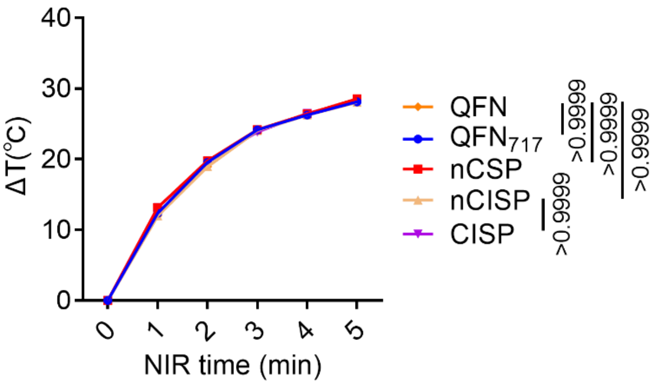

Supplementary Figure 6. Temperature curves of QFN, QFN_{717}, nCSP, nCISP and CISP treated with laser irradiation (1.0 W/cm^2). The concentration of QFN is 200 μg/mL in all groups. Data represent mean ± SEM, $n = 3$ independent samples. Statistical significance was determined by two-way ANOVA test, and it was two-sided and adjustments were made for multiple comparisons. Source data are provided as a Source Data file. QFN, Quercetin-ferrum nanoparticles; QFN_{717}, QFN loaded with SR-717; nCSP, normal-Cholesterol cell membrane coated QFN_{717}; nCISP, normal-Cholesterol cell membrane coated ICB agent and QFN_{717}; CISP, low-Cholesterol membrane coated ICB agent and QFN_{717}.

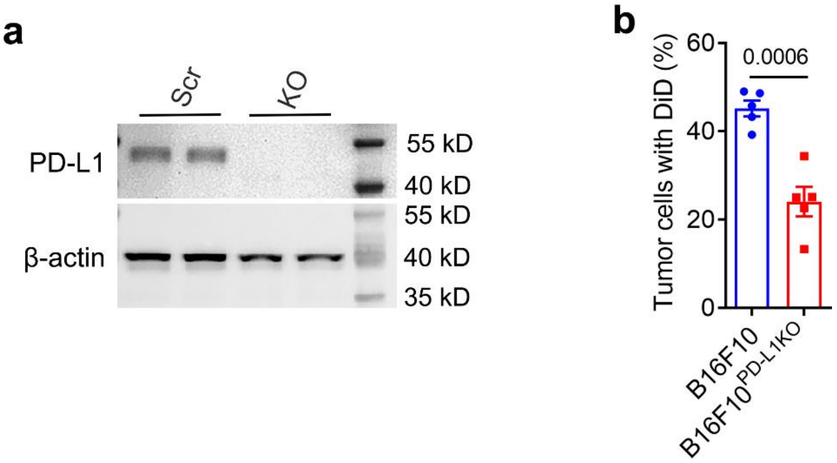

Supplementary Figure 7. The uptake of CISP by B16F10$^{PD\text{-}L1\ KO}$ cells. a Western blot analysis of B16F10 cells transfected with Control Double Nickase Plasmid (Scramble, Scr), and PD-L1 Double Nickase Plasmids (KO). **b** The uptake of CISP by Scramble B16F10 cells and B16F10$^{PD\text{-}L1\ KO}$ cells detected by flow-cytometry. Data represent mean ± SEM, n = 5 independent samples. Student's two-sided t test was used for the statistical analysis. The experiments for **a**, **b** were repeated three times independently with similar results. Source data are provided as a Source Data file.

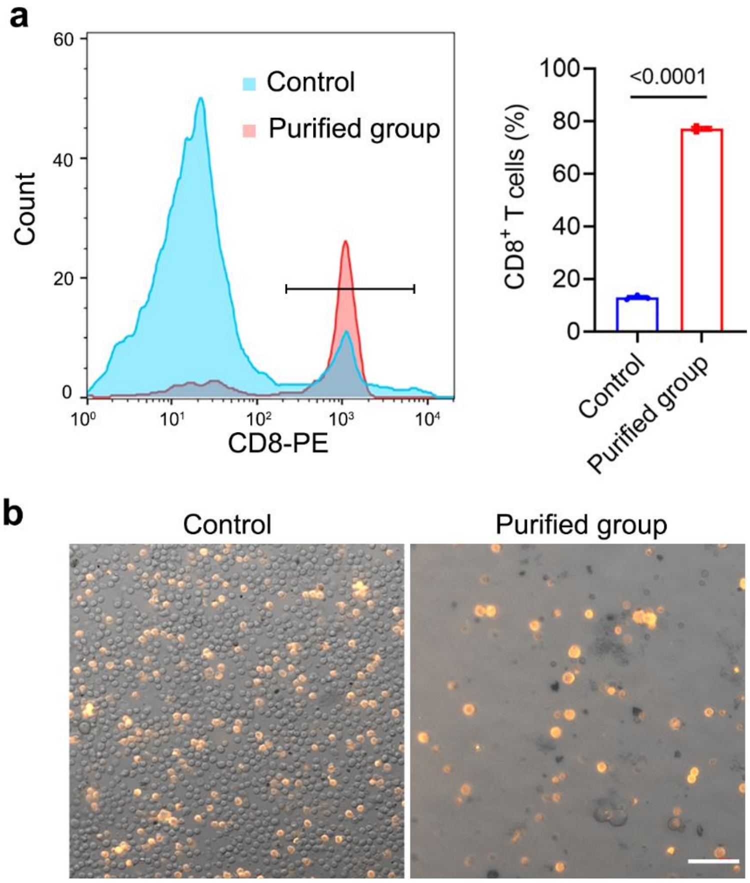

Supplementary Figure 8. Characterization of purified CD8$^+$ T cells. a Flow-cytometric analysis of CD8$^+$ T cells in splenocytes (Control) and purified splenocytes (Purified group). MojoSort™ Isolation Kits was used to isolate CD8$^+$ T cells in the spleens of mice. Data represent mean ± SEM, n = 3 independent samples. Student's two-sided t test was used for the statistical analysis. **b** Representative images of CD8$^+$ T cells (yellow) in splenocytes (Control) and purified splenocytes (Purified group). n = 3 independent samples. Scale bar = 50 μm. The experiments for **b** were repeated twice independently with similar results. Source data are provided as a Source Data file.

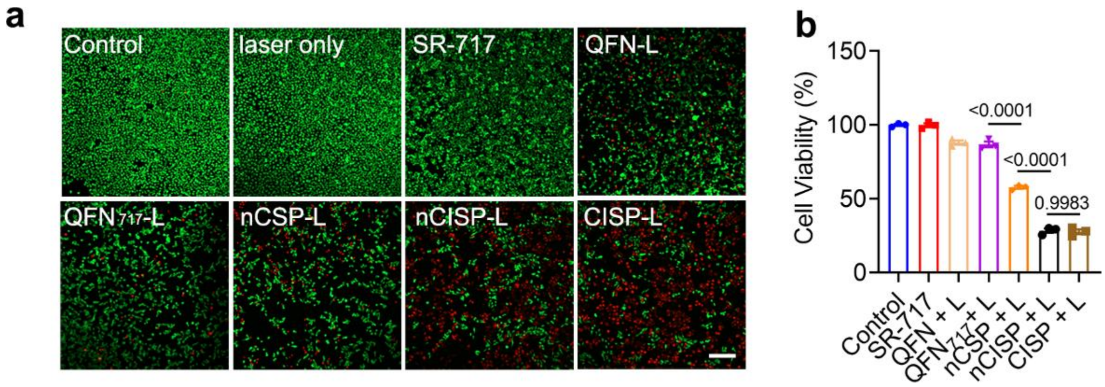

Supplementary Figure 9. Cell viability of B16F10 cells. a Representative images of B16F10 cells stained with fluorescein diacetate (green) and propidium iodide (red). B16F10 cells were incubated with CISP (the dosage of QFN is 30 μg/ml) for 8 h. Then cells were irradiated for 5 min (808 nm, 1.0 W/cm^2), and cell viability was examined at 1 h later. Scale bar = 150 μm. n = 3 independent samples. **b** B16F10 cells were treated with the indicated treatments, and cell viability was detected by CCK-8 kit. Data represent mean ± SEM, n = 3 independent samples. Statistical significance was determined by one-way ANOVA test, and it was two-sided and adjustments were made for multiple comparisons. The experiments for **a**, **b** were repeated three times independently with similar results. Source data are provided as a Source Data file. L, laser. QFN, Quercetin-ferrum nanoparticles; QFN_{717}, QFN loaded with SR-717; nCSP, normal-Cholesterol cell membrane coated QFN_{717}; nCISP, normal-Cholesterol cell membrane coated ICB agent and QFN_{717}; CISP, low-Cholesterol membrane coated ICB agent and QFN_{717}.

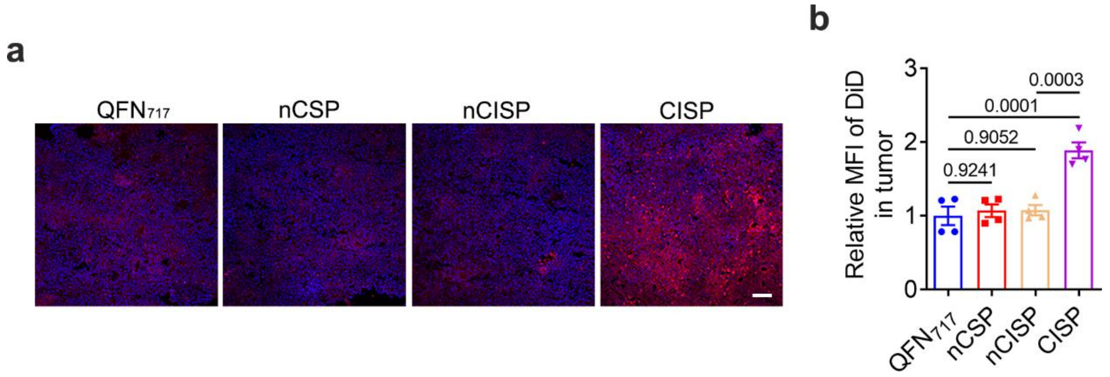

Supplementary Figure 10. Biodistribution of QFN_{717}, nCSP, nCISP and CISP in tumors. a, **b** Representative images of nanoparticles (red) in tumors (**a**) and the relative mean fluorescence intensity (MFI) of DiD in tumors (**b**). Scale bar = 150 μm. Mice bearing B16F10 melanoma were treated with QFN_{717}, nCSP, nCISP and CISP (marked with DiD, 6 μg/mouse, iv.), and mice were sacrificed at 6 h post injection. Data represent mean ± SEM, $n = 3$ mice. Statistical significance was determined by one-way ANOVA test, and it was two-sided and adjustments were made for multiple comparisons. The experiments for **a**, **b** were repeated three times independently with similar results. Source data are provided as a Source Data file. QFN, Quercetin-ferrum nanoparticles; QFN_{717}, QFN loaded with SR-717; nCSP, normal-Cholesterol cell membrane coated QFN_{717}; nCISP, normal-Cholesterol cell membrane coated ICB agent and QFN_{717}; CISP, low-Cholesterol membrane coated ICB agent and QFN_{717}.

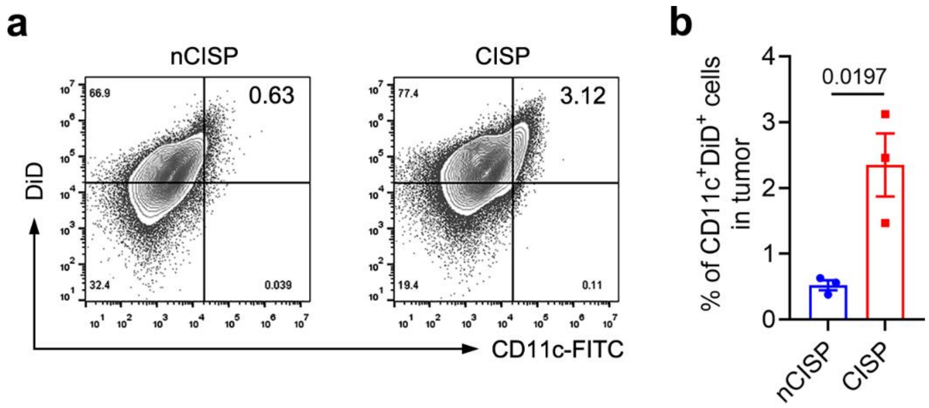

Supplementary Figure 11. Flow-cytometric analysis of the uptake of nCISP and CISP by DCs in tumors. a, **b** Representative flow cytometry plot (**a**) and the proportion of DiD$^+$ DCs in tumors (**b**). Mice bearing B16F10 melanoma were treated with nCISP and CISP (marked with DiD, 6 μg/mouse, iv.), and cells in tumors were isolated at 6 h post injection. Cells were incubated with red blood cell lysis buffer and stained with CD11c-FITC. Data represent mean ± SEM, *n* = 3 mice. Student's two-sided *t* test was used for the statistical analysis. Source data are provided as a Source Data file. QFN, Quercetin-ferrum nanoparticles; QFN_{717}, QFN loaded with SR-717; nCISP, normal-Cholesterol cell membrane coated ICB agent and QFN_{717}; CISP, low-Cholesterol membrane coated ICB agent and QFN_{717}; DCs, dendritic cells.

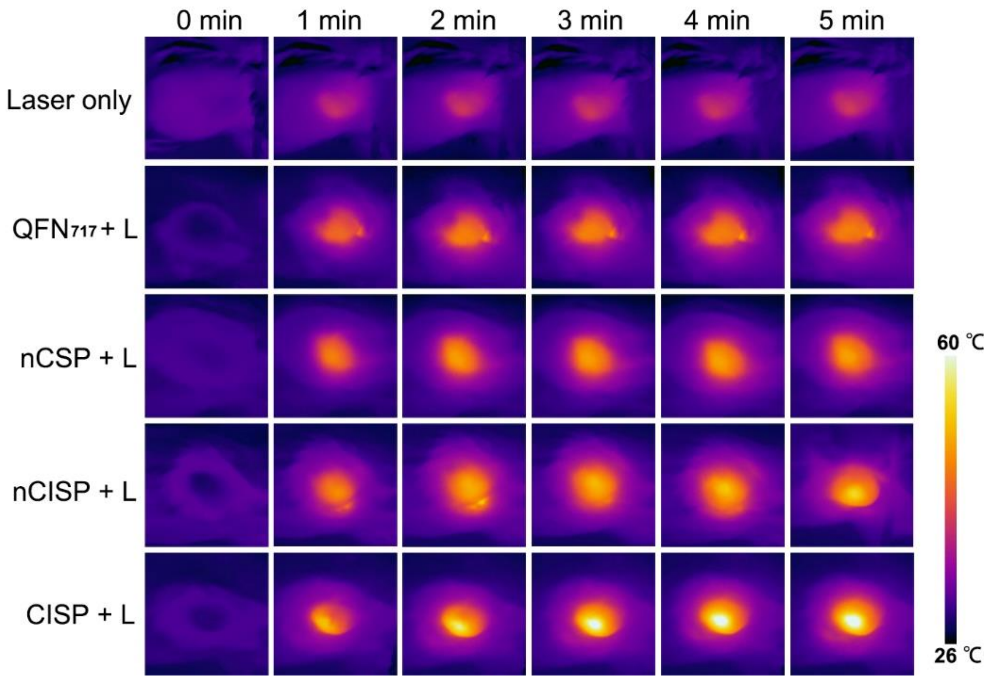

Supplementary Figure 12. Representative infrared thermal images of mice. Mice bearing B16F10 melanoma were treated with QFN_{717}, nCSP, nCISP or CISP, and tumors were irradiated for 5 min with a near-infrared laser (808 nm, 1.0 W/cm^2) at 12 h later. $n = 3$ mice. The experiments were repeated twice independently with similar results. L, laser; QFN, Quercetin-ferrum nanoparticles; QFN_{717}, QFN loaded with SR-717; nCSP, normal-Cholesterol cell membrane coated QFN_{717}; nCISP, normal-Cholesterol cell membrane coated ICB agent and QFN_{717}; CISP, low-Cholesterol membrane coated ICB agent and QFN_{717}.

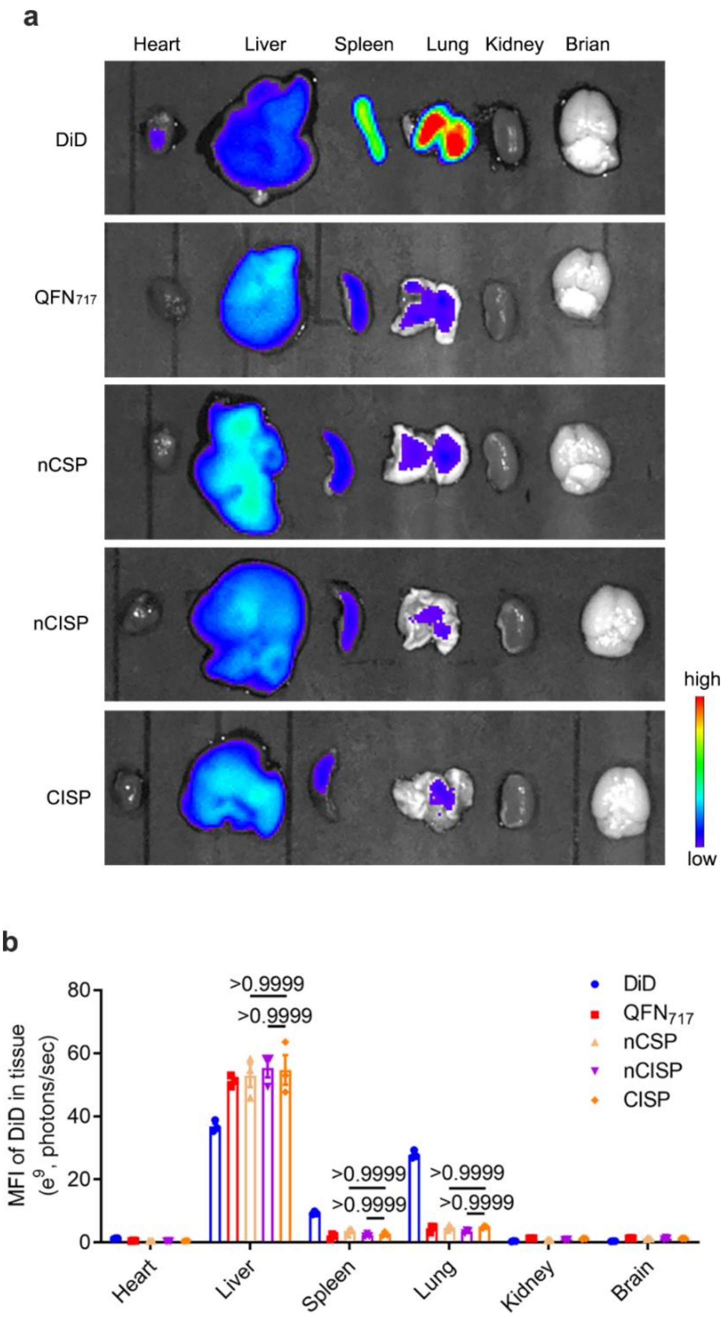

Supplementary Figure 13. Biodistribution of DiD, QFN_{717}, nCSP, nCISP and CISP in the major organs. a, **b** Representative fluorescence images of the major organs (**a**) and the mean fluorescence intensity (MFI) of DiD in the major organs (**b**). Mice bearing B16F10 melanoma were treated with DiD, QFN_{717}, nCSP, nCISP and CISP (marked with DiD, 6 μg/mouse, iv.), and mice were sacrificed at 24 h post injection. Data represent mean ± SEM, $n = 3$ mice. Statistical significance was determined by one-way ANOVA test, and it was two-sided and adjustments were made for multiple comparisons. The experiments for **a**, **b** were repeated twice independently with similar results. Source data are provided as a Source Data file. QFN, Quercetin-ferrum nanoparticles; QFN_{717}, QFN loaded with SR-717; nCSP, normal-Cholesterol cell membrane coated QFN_{717}; nCISP, normal-Cholesterol cell membrane coated ICB agent and QFN_{717}; CISP, low-Cholesterol membrane coated ICB agent and QFN_{717}.

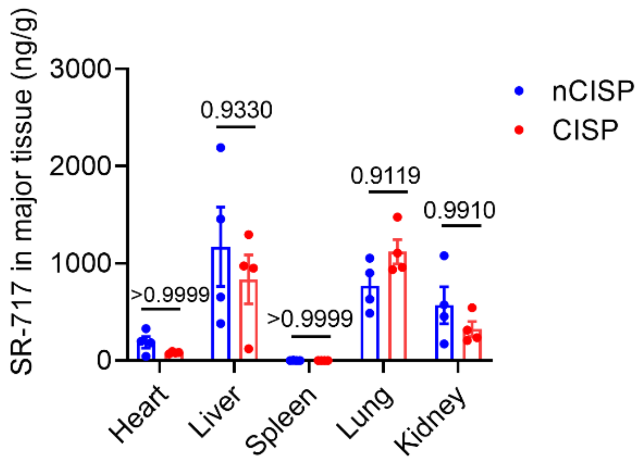

Supplementary Figure 14. Concentration of SR-717 in the major organs. Mice with B16F10 melanoma were treated with nCISP and CISP (10 mg/kg, iv.), and sacrificed at 8 h post injection. The concentration of SR-717 in the major organs was detected by LC-MS/MS. Data represent mean ± SEM, *n* = 4 mice. Statistical significance was determined by one-way ANOVA test, and it was two-sided and adjustments were made for multiple comparisons. Source data are provided as a Source Data file. QFN, Quercetin-ferrum nanoparticles; QFN_{717}, QFN loaded with SR-717; nCISP, normal-Cholesterol cell membrane coated ICB agent and QFN_{717}; CISP, low-Cholesterol membrane coated ICB agent and QFN_{717}.

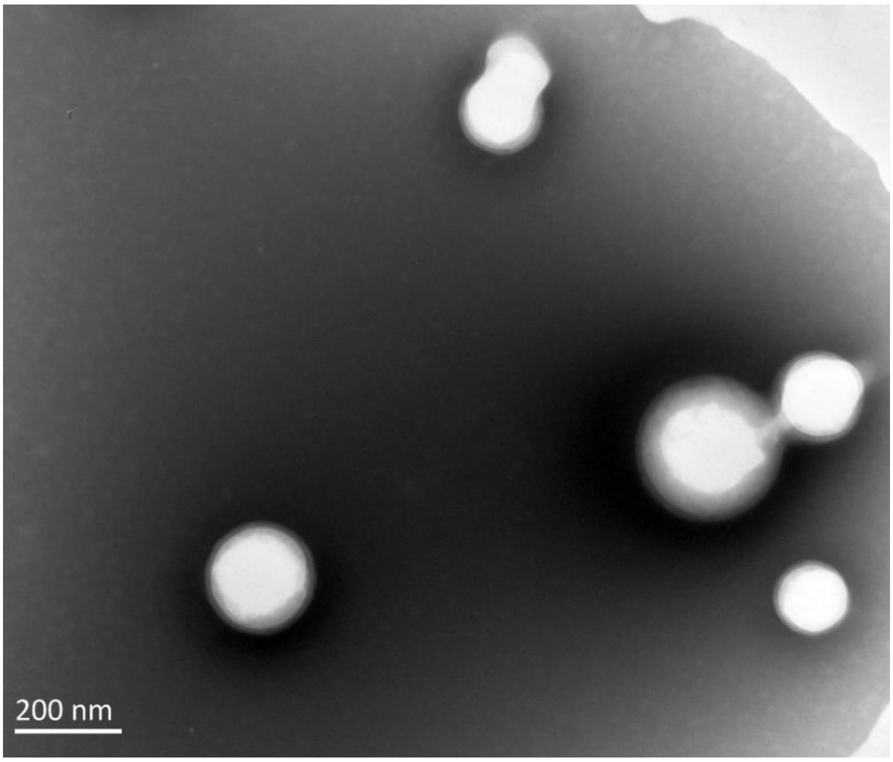

Supplementary Figure 15. Representative TEM image of CSP. *n* = 3 independent samples. The experiments were repeated twice independently with similar results. QFN, Quercetin-ferrum nanoparticles; QFN_{717}, QFN loaded with SR-717; CSP, low-Cholesterol membrane coated QFN_{717}.

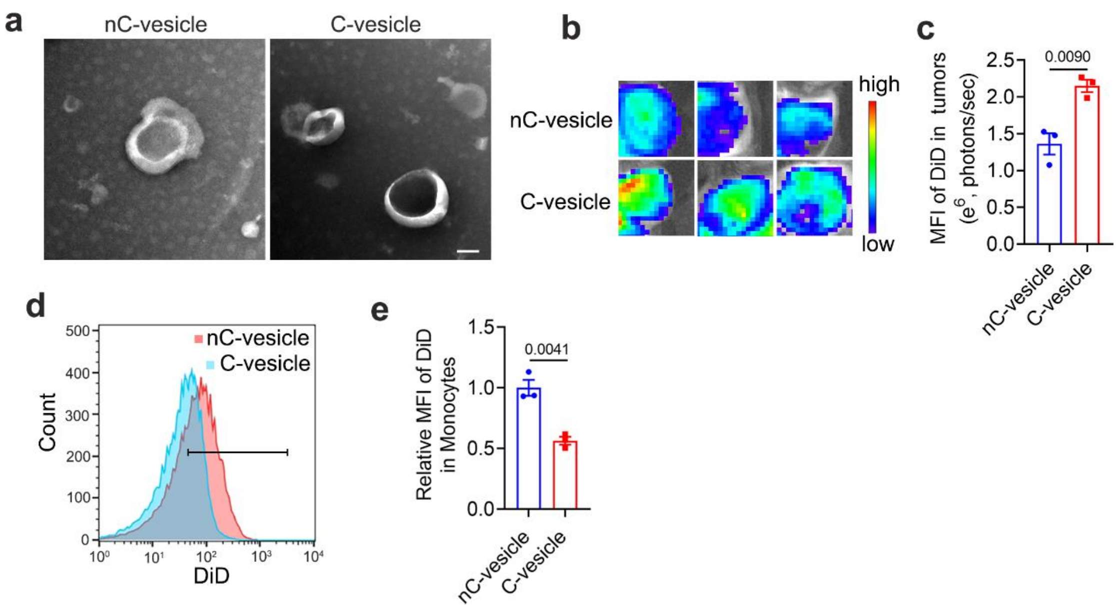

Supplementary Figure 16. Tumor targeting ability of cell membrane vesicle. a Representative TEM image of nC-vesicle (normal cell membrane vesicle sourced from CTLL2-PD1 cells) and C-vesicle (cell membrane vesicle sourced from CTLL2-PD1 cells that were treated with β-CD). Scale bar = 50 nm. **b**, **c** Fuorescence images of B16F10 melanoma at 6 h post injection of nC-vesicle and C-vesicle (1 μg DiD/mouse, iv.) (**b**), and the mean fluorescence intensity (MFI) of DiD in tumors (**c**). Data represent mean ± SEM, n = 3 mice. **d**, **e** The uptake of C-vesicle and nC-vesicle by monocytes in the blood detected by flow cytometry at 0.5 h post injection (**d**) and the relative mean fluorescence intensity (MFI) of DiD in monocytes (**e**). Data represent mean ± SEM, n = 3 mice. Student's two-sided t test was used for the statistical analysis. The experiments for **a**, **d**, **e** were repeated three times independently with similar results. Source data are provided as a Source Data file.

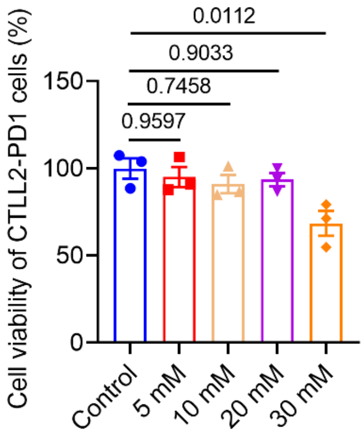

Supplementary Figure 17. Viability of CTLL2-PD1 cells treated with β-CD. Cells were washed with PBS once and incubated with different concentration of (2-hydroxypropyl)-β-cyclodextrin (5 mM, 10 mM, 20 mM and 30 mM) for 30 min, and then washed with PBS once. Cell viability was detected by CCK-8 kit. Data represent mean ± SEM, n = 3 independent samples. Statistical significance was determined by one-way ANOVA test, and it was two-sided and adjustments were made for multiple comparisons. The experiments were repeated three times independently with similar results. Source data are provided as a Source Data file.

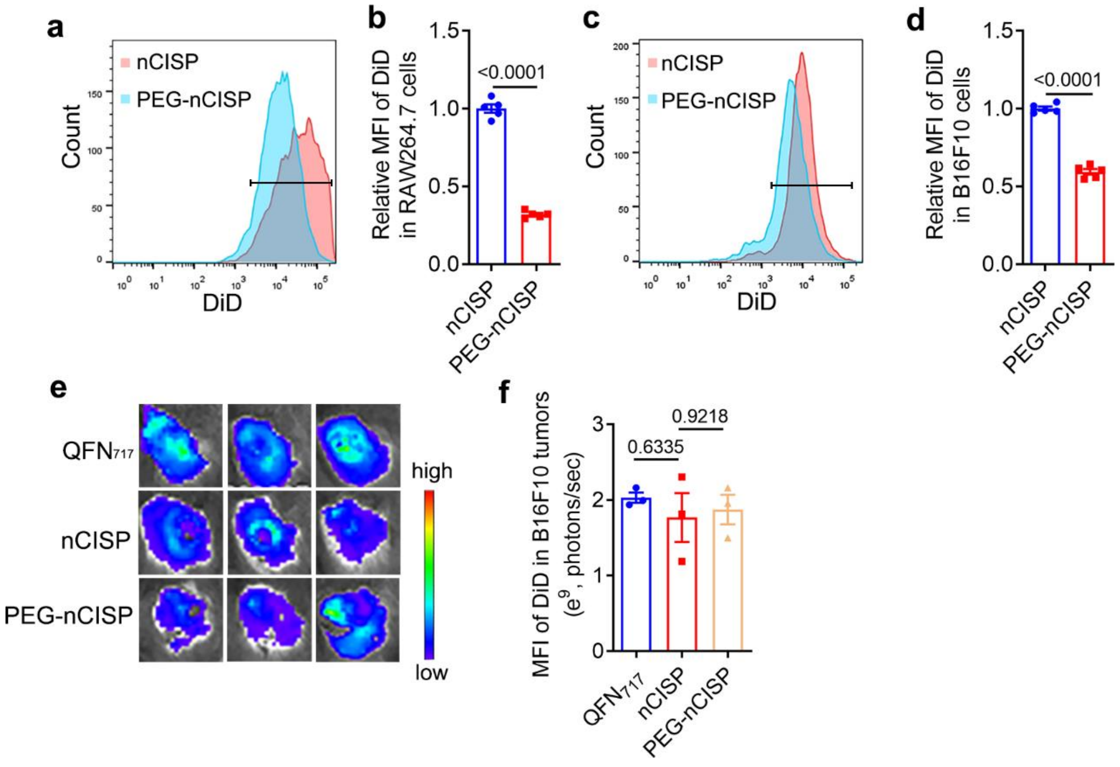

Supplementary Figure 18. Tumor targeting ability of PEG-nCISP. a, **b** The uptake of PEG-nCISP by RAW264.7 cells detected by flow-cytometry (**a**) and the mean fluorescence intensity (MFI) of DiD in RAW264.7 cells (**b**). Data represent mean ± SEM, n = 5 independent samples. **c**, **d** The uptake of PEG-nCISP by B16F10 cells detected by flow-cytometry (**c**) and the mean fluorescence intensity of DiD in B16F10 cells (**d**). Data represent mean ± SEM, n = 5 independent samples. **e**, **f** Fuorescence images of B16F10 melanoma at 12 h post injection of QFN_{717}, nCISP and PEG-nCISP (**e**), and the mean fluorescence intensity of DiD in tumors (**f**). Data represent mean ± SEM, n = 3 mice. Student's two-sided t test was used for the statistical analysis in **b**, **d**. Statistical significance was determined by one-way ANOVA test in **f**, and it was two-sided and adjustments were made for multiple comparisons. The experiments for **a**, **b**, **c**, **d** were repeated three times independently with similar results. The experiments for **e**, **f** were repeated twice independently with similar results. Source data are provided as a Source Data file. QFN, Quercetin-ferrum nanoparticles; QFN_{717}, QFN loaded with SR-717; nCISP, normal-Cholesterol cell membrane coated ICB agent and QFN_{717}; PEG-nCISP, normal-Cholesterol cell membrane with PEG coated ICB agent and QFN_{717}.

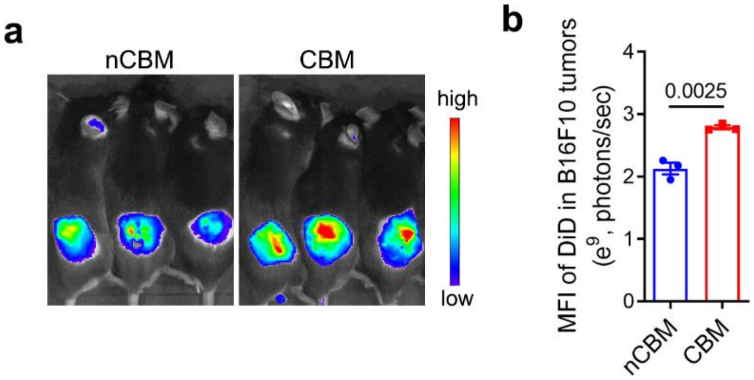

Supplementary Figure 19. Tumor targeting ability of CBM. a, **b** Fluorescence images of B16F10 melanoma at 12 h post injection of nCBM and CBM (**a**), and the mean fluorescence intensity (MFI) of DiD in tumors (**b**). Data represent mean ± SEM, n = 3 mice. Student's two-sided t test was used for the statistical analysis in **b**. The experiments were repeated three times independently with similar results. Source data are provided as a Source Data file. CBM, low-Cholesterol cell membrane sourced from B16F10 cells coated PLGA nanoparticles; nCBM, normal-Cholesterol cell membrane sourced from B16F10 cells coated PLGA nanoparticles.

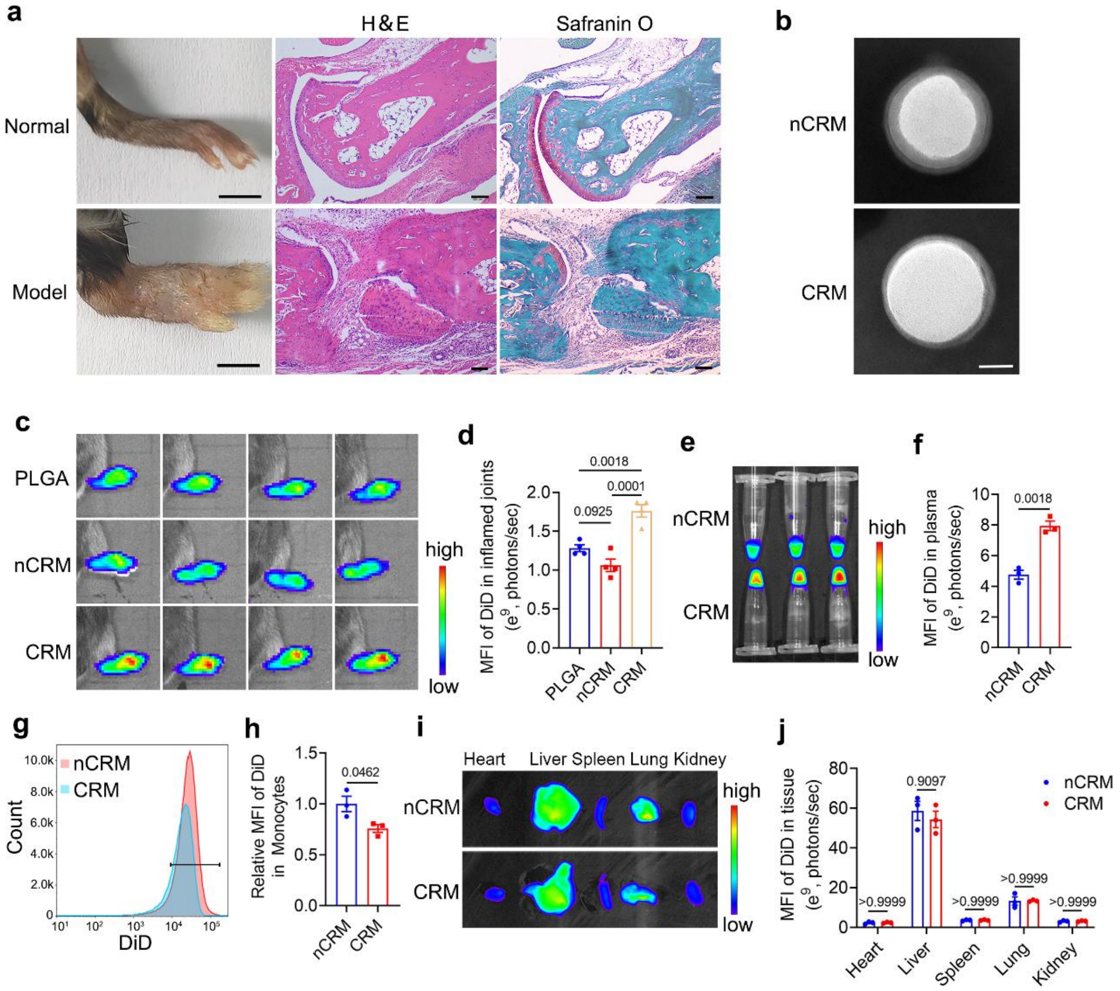

Supplementary Figure 20. Targeting ability of CRM in an arthritis model. a Representative image of ankle joints (Scale bar = 0.3 cm), H&E (Scale bar = 100 µm) and Safranin-O and toluidine blue staining of ankle joints (Scale bar = 100 µm). *n* = 3 mice. **b** Representative TEM images of nCRM and CRM. Scale bar = 50 nm. *n* = 3 independent samples. **c**, **d** Fluorescence images of model ankle joints at 6 h post injection of nCRM and CRM (**c**), and the mean fluorescence intensity (MFI) of DiD in model ankle joints (**d**). Data represent mean ± SEM, *n* = 4 mice. **e**, **f** Fluorescence images of plasma at 0.5 h post injection of particles (**e**), and the mean fluorescence intensity of DiD in the plasma (**f**). Data represent mean ± SEM, *n* = 3 mice. **g**, **h** The uptake of nCRM and CRM by monocytes in the blood detected by flow-cytometry (**g**), and the relative mean fluorescence intensity of DiD in monocytes (**h**). Data represent mean ± SEM, *n* = 3 mice. **i**, **j** Representative fluorescence images of major organs at 0.5 h post injection of nCRM and CRM (**i**), and the mean fluorescence intensity of DiD in the major organs (**j**). Data represent mean ± SEM, *n* = 3 mice. Student's two-sided *t* test was used for the statistical analysis in **d**, **f**, **h**. Statistical significance was determined by one-way ANOVA test in **j**, and it was two-sided and adjustments were made for multiple comparisons. The experiments for **b**, **g**, **h** were repeated three times independently with similar results. The experiments for **a** were repeated twice independently with similar results. Source data are provided as a Source Data file. nCRM, normal-Cholesterol cell membrane sourced from RAW264.7 cells coated

PLGA nanoparticles. CRM, low-Cholesterol cell membrane sourced from RAW264.7 cells coated PLGA nanoparticles.

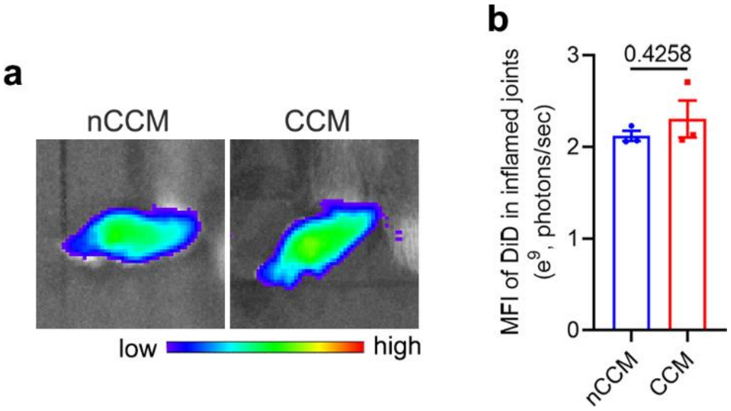

Supplementary Figure 21. Targeting ability of CCM in an arthritis model. a, **b** Fluorescence images of ankle joints at 6 h post injection of nCCM and CCM (**a**), and the mean fluorescence intensity (MFI) of DiD in ankle joints with arthritis (**b**). Data represent mean ± SEM, $n = 3$ mice. Student's two-sided t test was used for the statistical analysis in **b**. The experiments were repeated twice independently with similar results. Source data are provided as a Source Data file. nCCM, normal-Cholesterol cell membrane sourced from CTLL2-PD1 cells coated PLGA nanoparticles. CCM, low-Cholesterol cell membrane sourced from CTLL2-PD1 cells coated PLGA nanoparticles.

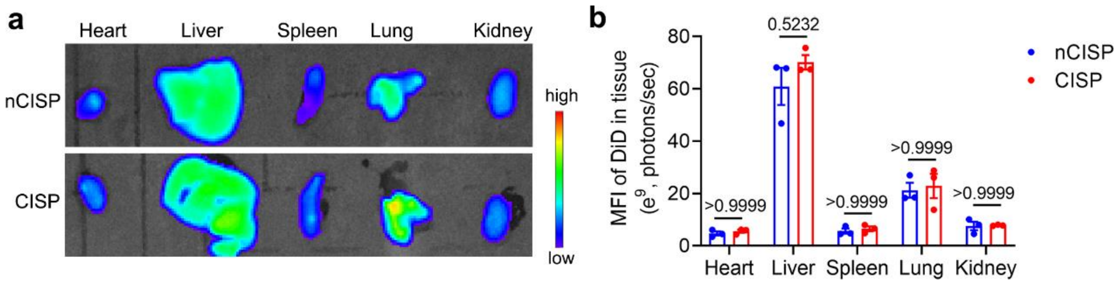

Supplementary Figure 22. Biodistribution of nCISP and CISP in the major organs at 0.5 h post injection. a, **b** Representative fluorescence images of major organs in mice (**a**), and the mean fluorescence intensity (MFI) of DiD in the major organs (**b**). Data represent mean ± SEM, *n* = 3 mice. Statistical significance was determined by one-way ANOVA test in **b**, and it was two-sided and adjustments were made for multiple comparisons. Source data are provided as a Source Data file. QFN, Quercetin-ferrum nanoparticles; QFN_{717}, QFN loaded with SR-717; nCISP, normal-Cholesterol cell membrane coated ICB agent and QFN_{717}; CISP, low-Cholesterol membrane coated ICB agent and QFN_{717}.

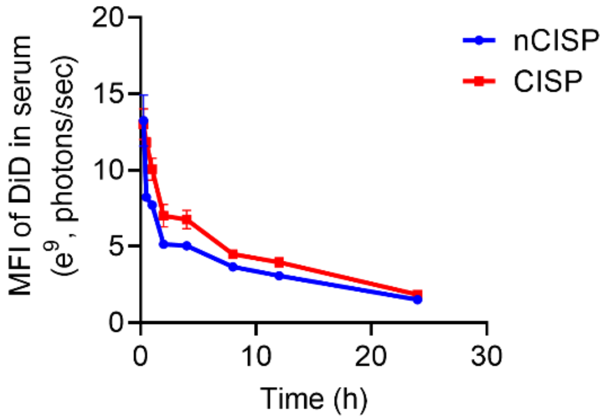

Supplementary Figure 23. Semi-quantitative analysis of nCISP and CISP in serum. Mice bearing B16F10 melanoma were treated with nCISP and CISP (marked with DiD), and sacrificed at different time points to isolate serum. The semi-quantitative analysis of nanoparticles in serum is based on the fluorescence images. Data represent mean ± SEM, *n* = 3 mice. Source data are provided as a Source Data file. QFN, Quercetin-ferrum nanoparticles; QFN_{717}, QFN loaded with SR-717; nCISP, normal-Cholesterol cell membrane coated ICB agent and QFN_{717}; CISP, low-Cholesterol membrane coated ICB agent and QFN_{717}.

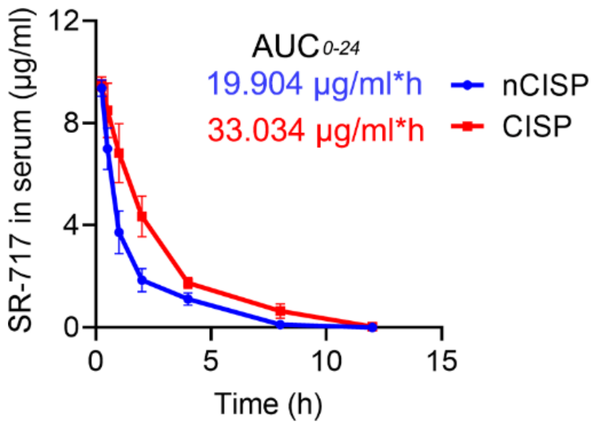

Supplementary Figure 24. Concentration of SR-717 in serum. Mice with B16F10 melanoma were treated with nCISP and CISP (10 mg/kg, iv.) and sacrificed at 0.25 h, 0.5 h, 2 h, 4 h, 8 h and 12 h. The concentration of SR-717 in serum was detected by LC-MS/MS. Data represent mean ± SEM, n = 5 mice. Source data are provided as a Source Data file. QFN, Quercetin-ferrum nanoparticles; QFN_{717}, QFN loaded with SR-717; nCISP, normal-Cholesterol cell membrane coated ICB agent and QFN_{717}; CISP, low-Cholesterol membrane coated ICB agent and QFN_{717}.

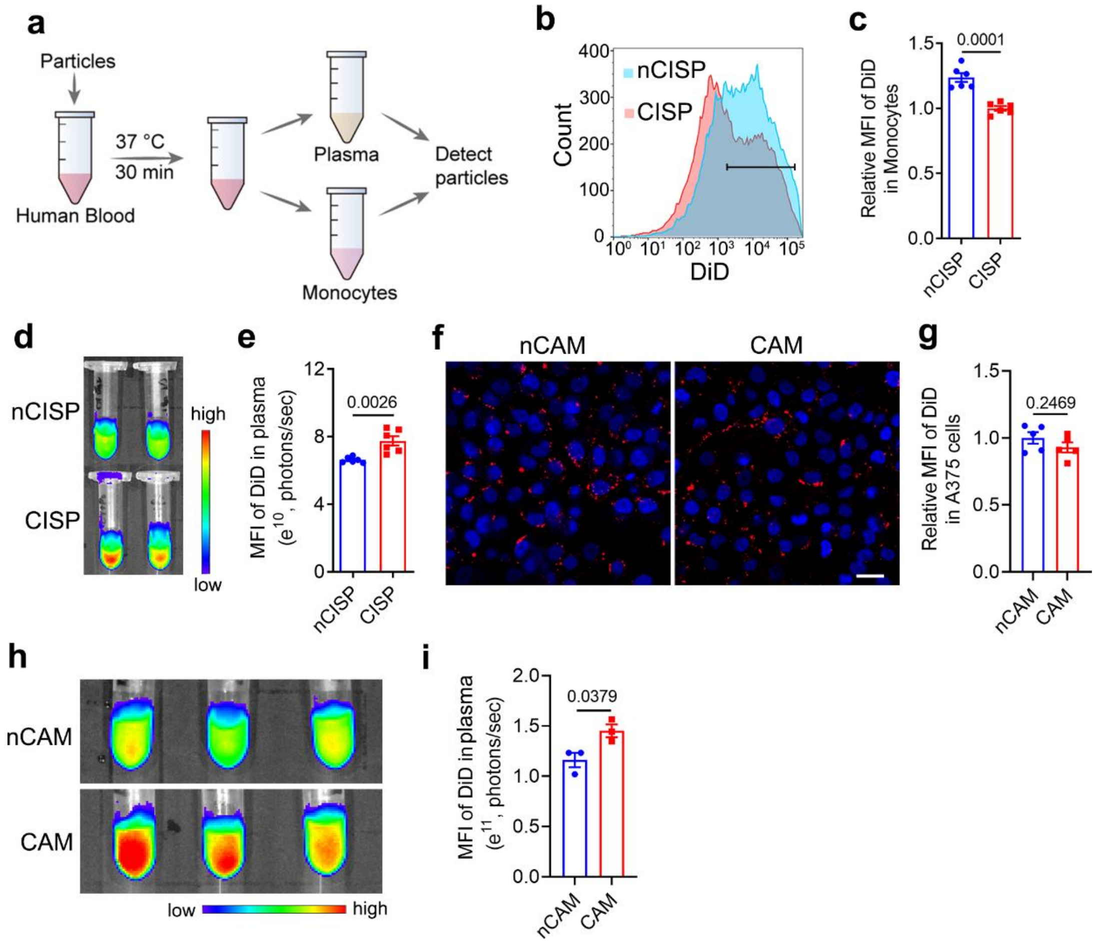

Supplementary Figure 25. Uptake of nanoparticles by monocytes in human blood. **a** Schematic illustration showing the process to detect CISP in human blood. **b**, **c** Uptake of nCISP and CISP by monocytes in human blood detected by flow-cytometry (**b**) and the relative mean fluorescence intensity (MFI) of DiD in monocytes (**c**). Data represent mean ± SEM, n = 6 independent samples. **d**, **e** Representative fluorescence images of plasma (**d**) and MFI of DiD in plasma (**e**). Data represent mean ± SEM, n = 6 independent samples. **f**, **g** Representative fluorescence images of A375 cells treated with nCAM and CAM (Red) (**f**), and the relative MFI of DiD in cells (**g**). Scale bar = 20 μm. Data represent mean ± SEM, n = 5 independent samples. **h**, **i** Fluorescence images of plasma at 0.5 h post incubation (**h**), and MFI of DiD in plasma (**i**). Data represent mean ± SEM, n = 3 independent samples. Student's two-sided t test was used for the statistical analysis in **c**, **e**, **g**, **i**. The experiments for **b**, **c**, **d**, **e**, **f**, **g** were repeated twice independently with similar results. Source data are provided as a Source Data file. QFN, Quercetin-ferrum nanoparticles; QFN_{717}, QFN loaded with SR-717; nCISP, normal-Cholesterol cell membrane coated ICB agent and QFN_{717}; CISP, low-Cholesterol membrane coated ICB agent and QFN_{717}; nCAM, normal-Cholesterol cell membrane sourced from A375 cells coated PLGA nanoparticles. CAM, low-Cholesterol cell membrane sourced from A375 cells coated PLGA nanoparticles.

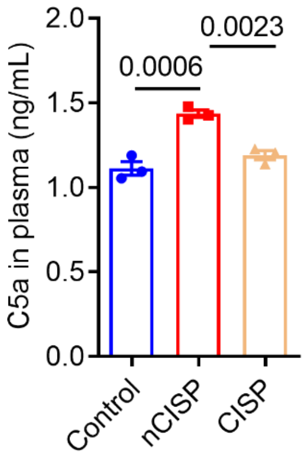

Supplementary Figure 26. Concentration of C5a in plasma. Mice with B16F10 melanoma were treated with nCISP and CISP (10 mg/kg), and the plasma was collected to detect C5a at 0.5 h post injection. Data represent mean ± SEM, *n* = 3 mice. Statistical significance was determined by one-way ANOVA test, and it was two-sided and adjustments were made for multiple comparisons. Source data are provided as a Source Data file. QFN, Quercetin-ferrum nanoparticles; QFN_{717}, QFN loaded with SR-717; nCISP, normal-Cholesterol cell membrane coated ICB agent and QFN_{717}; CISP, low-Cholesterol membrane coated ICB agent and QFN_{717}.

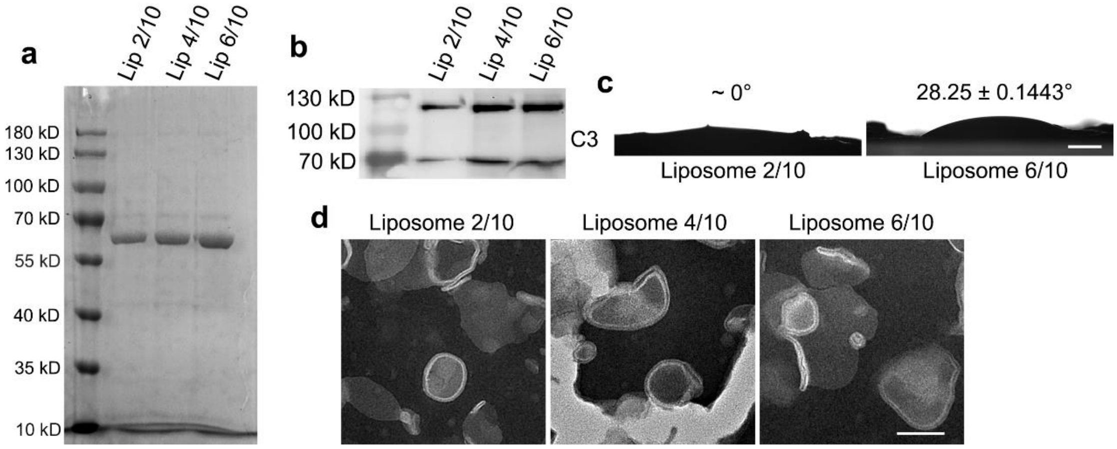

Supplementary Figure 27. Characterization of liposomes. **a** Images of coomassie brilliant blue staining of SDS-PAGE gel. Liposomes (0.5 mg/mL) with different content of cholesterol were incubated with serum of mice, and the protein absorbed on them were assessed then. **b** Western blot analysis of complement C3 absorbed on liposomes. **c** Water contact angle of Liposome 2/10 and Liposome 6/10 at 0.08s. Data represent mean ± SEM, $n = 3$ independent samples. **d** TEM images of Liposome 2/10, Liposome 4/10 and Liposome 6/10. Scale bar = 100 nm. The experiments for **a**, **b, c, d** were repeated twice independently with similar results. Lip 2/10, liposomes made from cholesterol (2 mg) and phospholipids (10 mg); Lip 4/10, liposomes made from cholesterol (4 mg) and phospholipids (10 mg); Lip 6/10, liposomes made from cholesterol (6 mg) and phospholipids (10 mg).

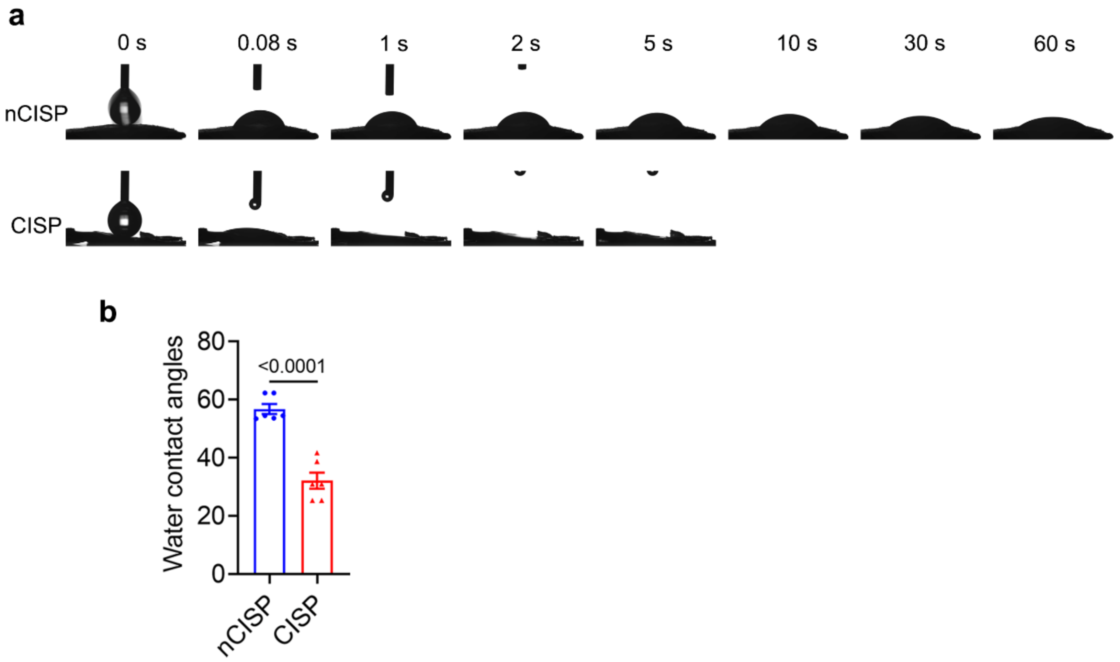

Supplementary Figure 28. Water contact angle of nCISP and CISP tableting on glass slices. a, **b** Representative images are show in (**a**), and water contact angle of nCISP and CISP at 0.08s are shown in (**b**). Data represent mean ± SEM, *n* = 6 independent samples. Student's two-sided *t* test was used for the statistical analysis in **b**. The experiments were repeated twice independently with similar results. Source data are provided as a Source Data file. QFN, Quercetin-ferrum nanoparticles; QFN_{717}, QFN loaded with SR-717; nCISP, normal-Cholesterol cell membrane coated ICB agent and QFN_{717}; CISP, low-Cholesterol membrane coated ICB agent and QFN_{717}.

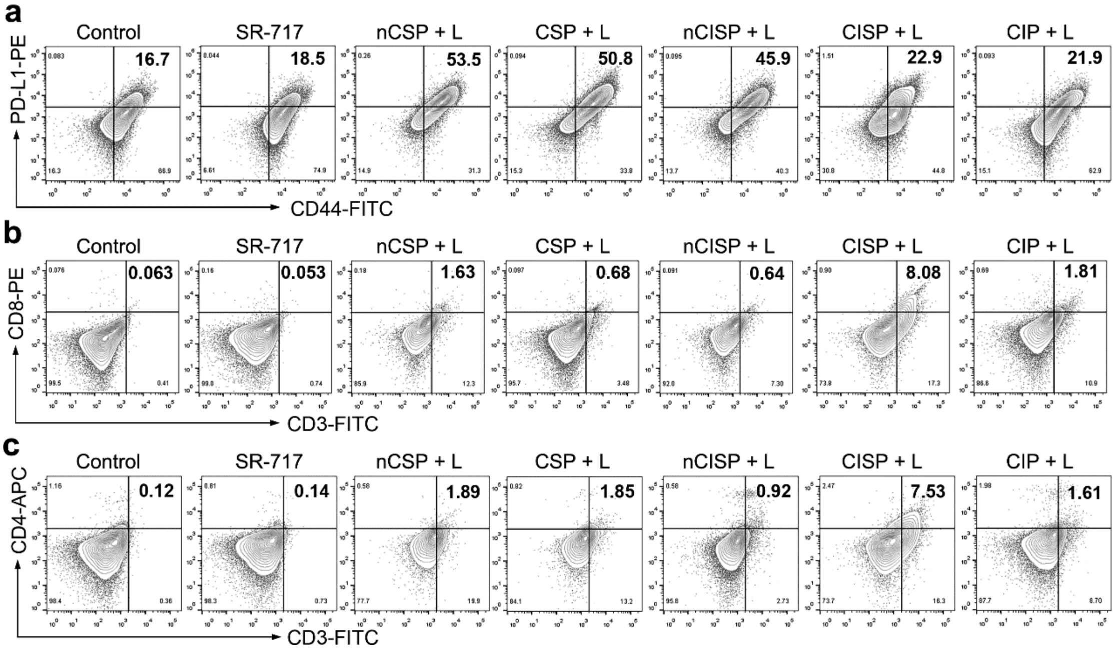

Supplementary Figure 29. The anti-tumor immunity activation in B16F10 melanoma. a Flow-cytometric analysis of $CD44^{+}PD\text{-}L1^{+}$ cells in tumors at 6 days post laser irradiation. n = 4 mice. **b** Flow-cytometric analysis of $CD3^{+}CD8^{+}$ cells in tumors at 6 days post laser irradiation. n = 4 mice. **c** Flow-cytometric analysis of $CD3^{+}CD4^{+}$ cells in tumors at 6 days post laser irradiation. n = 4 mice. L, laser; QFN, Quercetin-ferrum nanoparticles; QFN_{717}, QFN loaded with SR-717; nCSP, normal-Cholesterol cell membrane coated QFN_{717}; CSP, low-Cholesterol membrane coated QFN_{717}; nCISP, normal-Cholesterol cell membrane coated ICB agent and QFN_{717}; CISP, low-Cholesterol membrane coated ICB agent and QFN_{717}; CIP, low-Cholesterol membrane coated ICB agent and QFN.

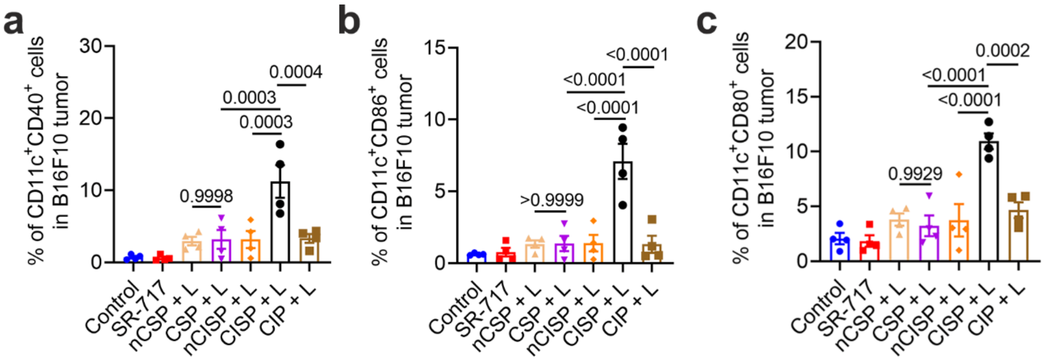

Supplementary Figure 30. Maturation of DCs in B16F10 melanoma. **a**, **b**, **c** Flow-cytometric analysis of $CD11c^+CD40^+$ cells (**a**), $CD11c^+CD86^+$ cells (**b**) and $CD11c^+CD80^+$ cells (**c**) in B16F10 melanoma at 6 days post laser irradiation. Data represent mean ± SEM, *n* = 4 mice. Statistical significance was determined by one-way ANOVA test in **a**, **b**, **c**, and it was two-sided and adjustments were made for multiple comparisons. Source data are provided as a Source Data file. L, laser; QFN, Quercetin-ferrum nanoparticles; QFN_{717}, QFN loaded with SR-717; nCSP, normal-Cholesterol cell membrane coated QFN_{717}; CSP, low-Cholesterol membrane coated QFN_{717}; nCISP, normal-Cholesterol cell membrane coated ICB agent and QFN_{717}; CISP, low-Cholesterol membrane coated ICB agent and QFN_{717}; CIP, low-Cholesterol membrane coated ICB agent and QFN.

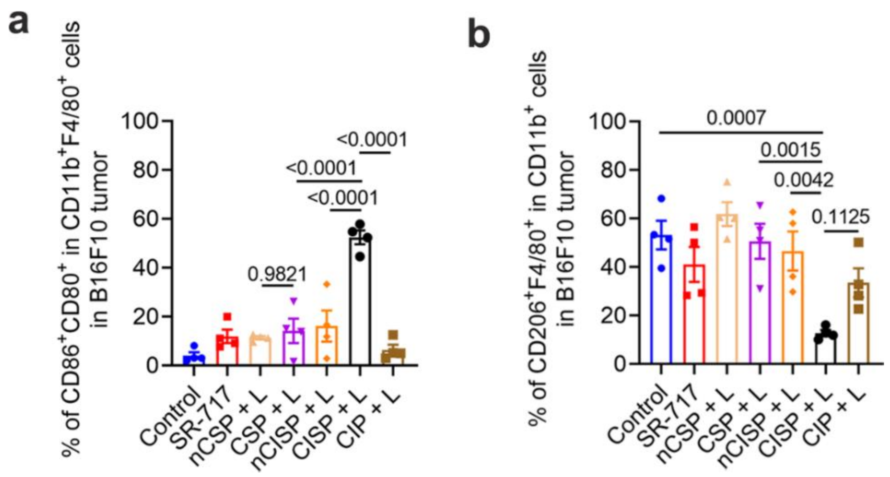

Supplementary Figure 31. Macrophages in B16F10 melanoma. **a** Flow-cytometric analysis of $CD80^+CD86^+$ cells in $CD11b^+F4/80^+$ cells (M1) in B16F10 melanoma at 6 days post laser irradiation. Data represent mean ± SEM, *n* = 4 mice. **b** Flow-cytometric analysis of $CD206^+F4/80^+$ cells in $CD11b^+$ cells (M2) in B16F10 melanoma at 6 days post laser irradiation. Data represent mean ± SEM, *n* = 4 mice. Statistical significance was determined by one-way ANOVA test in **a**, **b**, and it was two-sided and adjustments were made for multiple comparisons. Source data are provided as a Source Data file. L, laser; QFN, Quercetin-ferrum nanoparticles; QFN_{717}, QFN loaded with SR-717; nCSP, normal-Cholesterol cell membrane coated QFN_{717}; CSP, low-Cholesterol membrane coated QFN_{717}; nCISP, normal-Cholesterol cell membrane coated ICB agent and QFN_{717}; CISP, low-Cholesterol membrane coated ICB agent and QFN_{717}; CIP, low-Cholesterol membrane coated ICB agent and QFN.

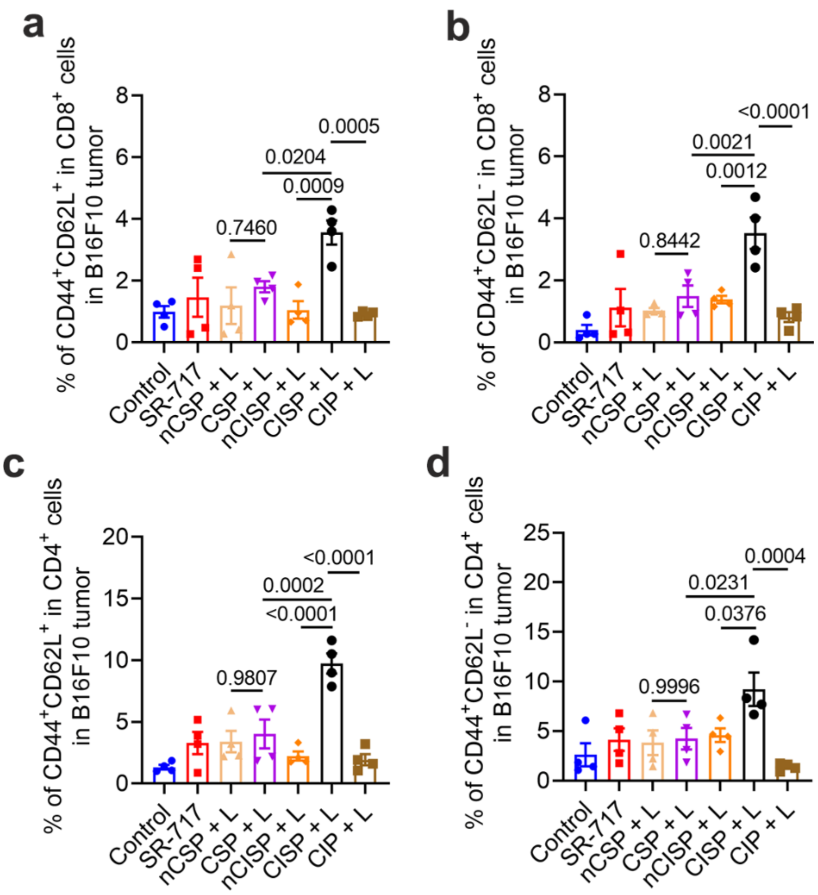

Supplementary Figure 32. Memory T cells in B16F10 melanoma. **a**, **b**, **c**, **d** Memory T cells including $CD8^{+}$Tcm (**a**), $CD8^{+}$Tem (**b**), $CD4^{+}$Tcm (**c**) and $CD4^{+}$Tem (**d**) in tumors were examined by flow cytometer. Tumors were harvested at 6 days post laser irradiation. Data represent mean ± SEM, n = 4 mice. Statistical significance was determined by one-way ANOVA test in **a**, **b**, **c**, **d** and it was two-sided and adjustments were made for multiple comparisons. Source data are provided as a Source Data file. L, laser; QFN, Quercetin-ferrum nanoparticles; QFN_{717}, QFN loaded with SR-717; nCSP, normal-Cholesterol cell membrane coated QFN_{717}; CSP, low-Cholesterol membrane coated QFN_{717}; nCISP, normal-Cholesterol cell membrane coated ICB agent and QFN_{717}; CISP, low-Cholesterol membrane coated ICB agent and QFN_{717}; CIP, low-Cholesterol membrane coated ICB agent and QFN.

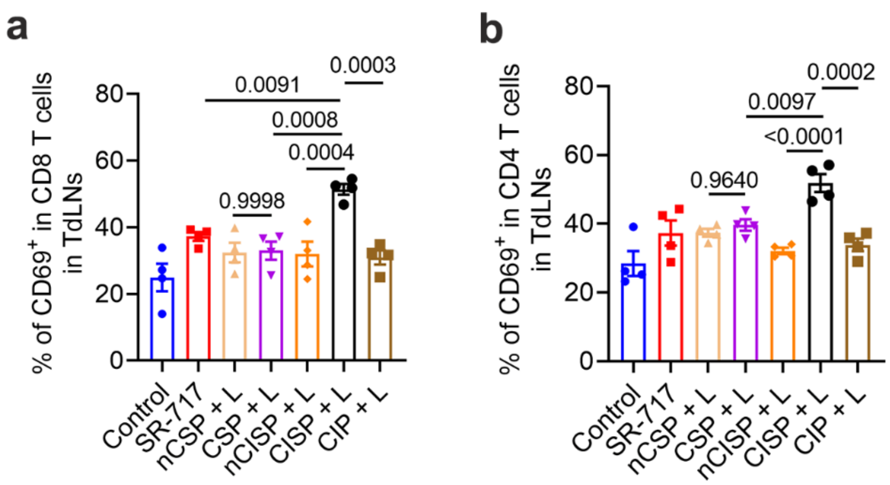

Supplementary Figure 33. Activated T cells in TdLNs in mice bearing B16F10 melanoma. a Flow-cytometric analysis of $CD69^+$ cells in $CD3^+CD8^+$ cells in TdLNs at 6 days post laser irradiation. Data represent mean ± SEM, *n* = 4 mice. **b** Flow-cytometric analysis of $CD69^+$ cells in $CD3^+CD4^+$ cells in TdLNs at 6 days post laser irradiation. Data represent mean ± SEM, *n* = 4 mice. Statistical significance was determined by one-way ANOVA test in **a**, **b** and it was two-sided and adjustments were made for multiple comparisons. Source data are provided as a Source Data file. L, laser, QFN, Quercetin-ferrum nanoparticles; QFN_{717}, QFN loaded with SR-717; nCSP, normal-Cholesterol cell membrane coated QFN_{717}; CSP, low-Cholesterol membrane coated QFN_{717}; nCISP, normal-Cholesterol cell membrane coated ICB agent and QFN_{717}; CISP, low-Cholesterol membrane coated ICB agent and QFN_{717}; CIP, low-Cholesterol membrane coated ICB agent and QFN; TdLNs, Tumor draining lymph nodes.

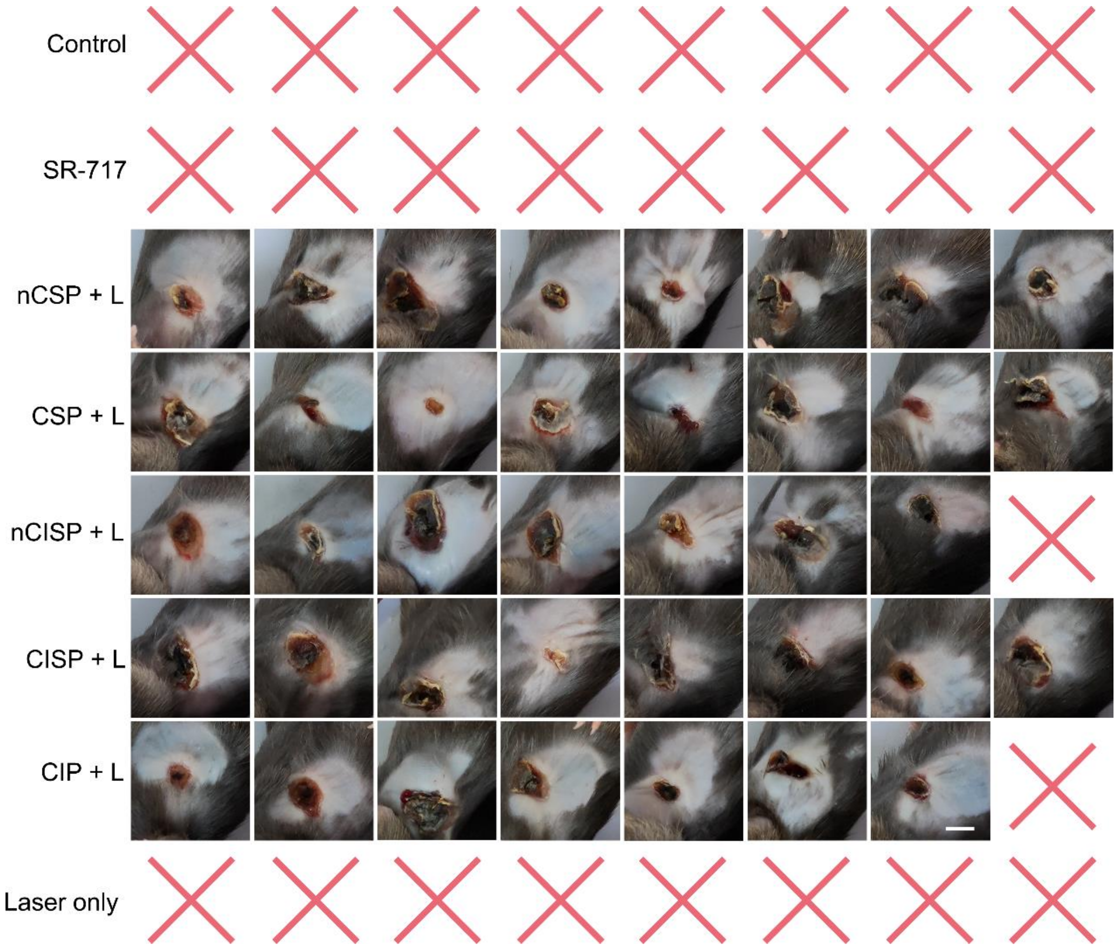

Supplementary Figure 34. Images of melanoma in mice at 16 days post tumor inoculation. Red cross line represents the dead mouse. Scale bar = 0.5 cm. n = 8 mice. L, laser; QFN, Quercetin-ferrum nanoparticles; QFN_{717}, QFN loaded with SR-717; nCSP, normal-Cholesterol cell membrane coated QFN_{717}; CSP, low-Cholesterol membrane coated QFN_{717}; nCISP, normal-Cholesterol cell membrane coated ICB agent and QFN_{717}; CISP, low-Cholesterol membrane coated ICB agent and QFN_{717}; CIP, low-Cholesterol membrane coated ICB agent and QFN.

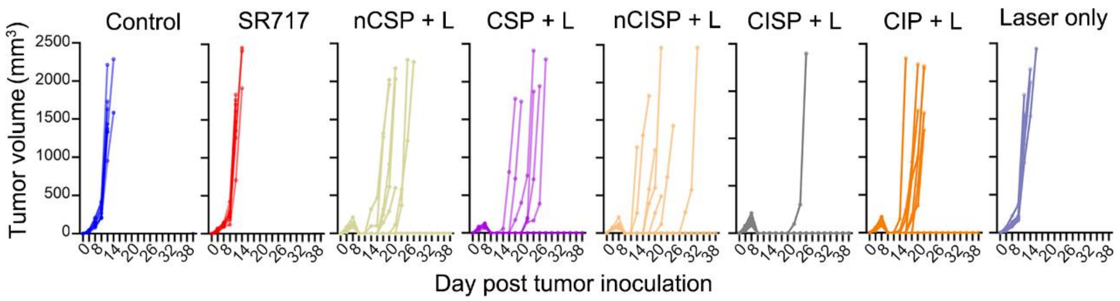

Supplementary Figure 35. Tumor growth curve of mice bearing B16F10 melanoma. *n* = 8 mice. Source data are provided as a Source Data file. L, laser; QFN, Quercetin-ferrum nanoparticles; QFN_{717}, QFN loaded with SR-717; nCSP, normal-Cholesterol cell membrane coated QFN_{717}; CSP, low-Cholesterol membrane coated QFN_{717}; nCISP, normal-Cholesterol cell membrane coated ICB agent and QFN_{717}; CISP, low-Cholesterol membrane coated ICB agent and QFN_{717}; CIP, low-Cholesterol membrane coated ICB agent and QFN.

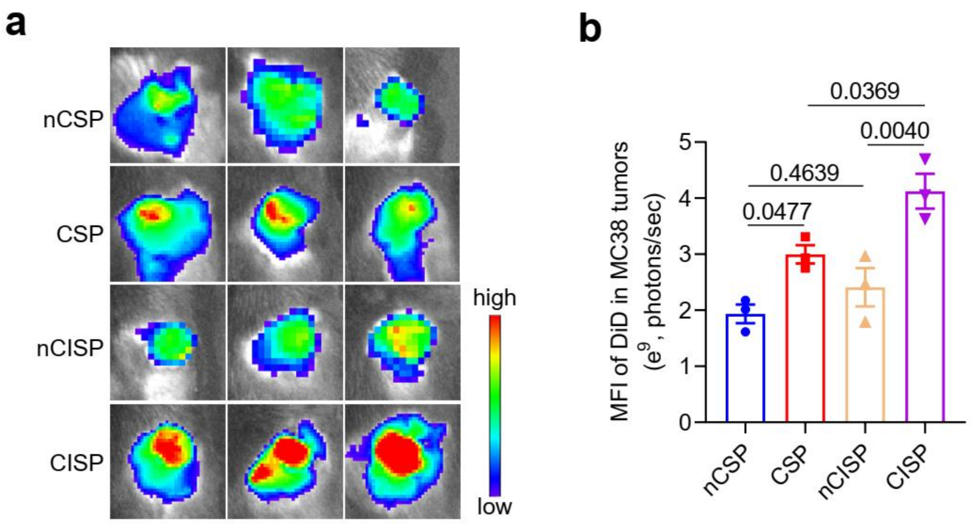

Supplementary Figure 36. CISP targeted to MC38 tumors in mice. a, **b** Fluorescence images of MC38 tumors at 6 h post treated with of nCSP, CSP, nCISP and CISP (**a**) and the mean fluorescence intensity (MFI) of DiD in tumors (**b**). Data represent mean ± SEM, $n = 3$ mice. The experiments were repeated twice independently with similar results. Statistical significance was determined by one-way ANOVA test in **b** and it was two-sided and adjustments were made for multiple comparisons. Source data are provided as a Source Data file. QFN, Quercetin-ferrum nanoparticles; QFN_{717}, QFN loaded with SR-717; nCSP, normal-Cholesterol cell membrane coated QFN_{717}; CSP, low-Cholesterol membrane coated QFN_{717}; nCISP, normal-Cholesterol cell membrane coated ICB agent and QFN_{717}; CISP, low-Cholesterol membrane coated ICB agent and QFN_{717}.

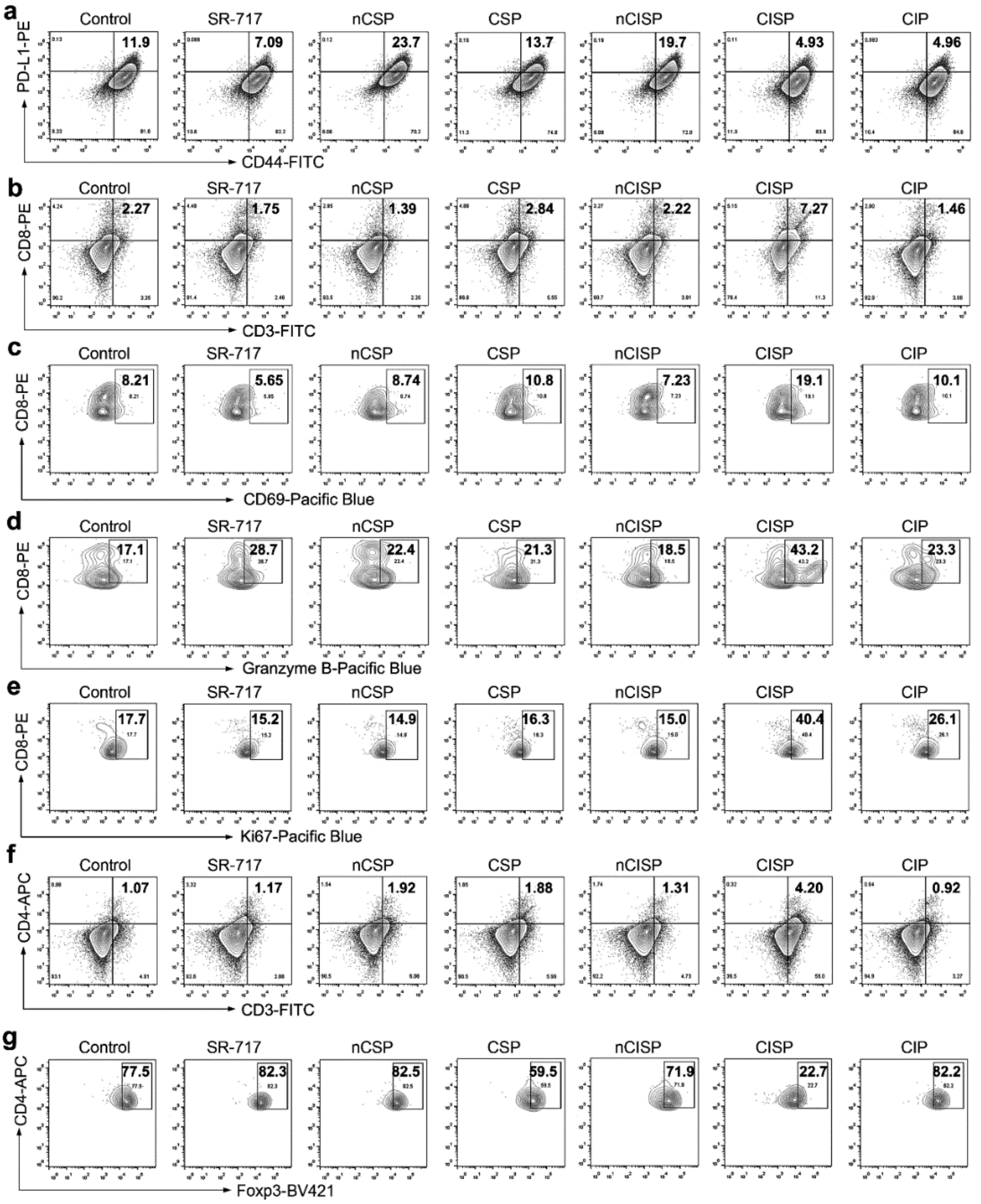

Supplementary Figure 37. The anti-tumor immunity activation in MC38 tumors. **a** Flow-cytometric analysis of $CD44^{+}PD\text{-}L1^{+}$ cells in MC38 tumors at 18 days post tumor inoculation. *n* = 4 mice. **b** Flow-cytometric analysis of $CD3^{+}CD8^{+}$ cells in MC38 tumors at 18 days post tumor inoculation. *n* = 4 mice. **c** Flow-cytometric analysis of $CD69^{+}$ cells in CD8 T cells in MC38 tumors at 18 days post tumor inoculation. (*n* = 4 mice). **d** Flow-cytometric analysis of Granzyme B^{+} cells in CD8 T cells in MC38 tumors at 18 days post tumor inoculation. *n* = 4 mice. **e** Flow-cytometric analysis of $Ki67^{+}$ cells in CD8 T cells in MC38 tumors at 18 days post tumor inoculation. *n* = 4 mice. **f** Flow-cytometric analysis of $CD3^{+}CD4^{+}$ cells in MC38 tumors at 18 days post tumor inoculation. *n* = 4 mice. **g** Flow-cytometric analysis of $Foxp3^{+}$ cells in CD4 T cells in MC38 tumors at 18 days post tumor inoculation. *n* = 4 mice. QFN, Quercetin-

ferrum nanoparticles; QFN_{717}, QFN loaded with SR-717; nCSP, normal-Cholesterol cell membrane coated QFN_{717}; CSP, low-Cholesterol membrane coated QFN_{717}; nCISP, normal-Cholesterol cell membrane coated ICB agent and QFN_{717}; CISP, low-Cholesterol membrane coated ICB agent and QFN_{717}; CIP, low-Cholesterol membrane coated ICB agent and QFN.

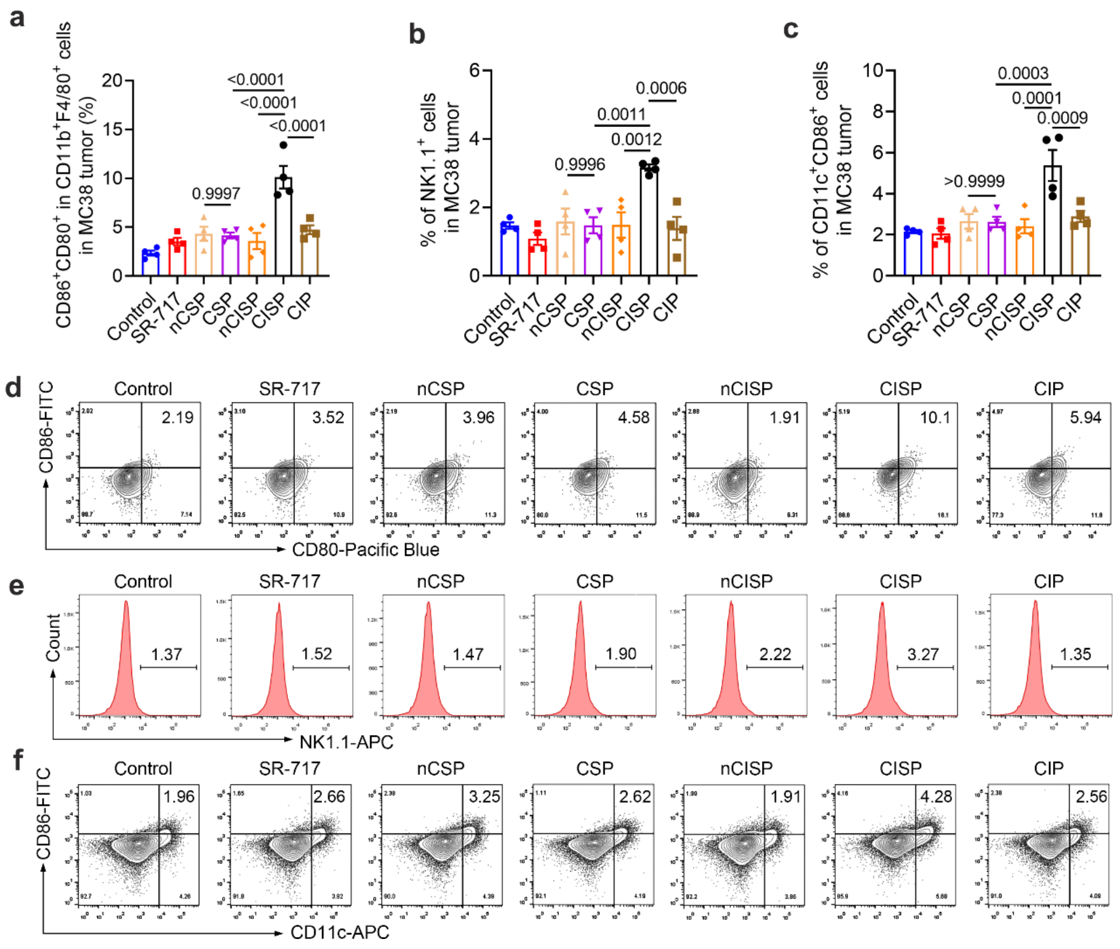

Supplementary Figure 38. Macrophages, NK cells and DCs in MC38 tumors. a Flow-cytometric analysis of CD80$^+$CD86$^+$ cells in CD11b$^+$F4/80$^+$ cells in MC38 tumors at 18 days post tumor inoculation. Data represent mean ± SEM, *n* = 4 mice. **b** Flow-cytometric analysis of NK1.1$^+$ cells in MC38 tumors at 18 days post tumor inoculation. Data represent mean ± SEM, *n* = 4 mice. **c** Flow-cytometric analysis of CD11c$^+$CD86$^+$ cells in MC38 tumors at 18 days post tumor inoculation. Data represent mean ± SEM, *n* = 4 mice. **d** Representative flow cytometry dot plot of CD80$^+$CD86$^+$ cells in CD11b$^+$F4/80$^+$ cells in MC38 tumors. *n* = 4 mice. **e** Representative flow cytometry dot plot of NK1.1$^+$ cells in MC38 tumors. *n* = 4 mice. **f** Representative flow cytometry dot plot of CD11c$^+$CD86$^+$ in MC38 tumors. *n* = 4 mice. Statistical significance was determined by one-way ANOVA test in **a**, **b**, **c**, and it was two-sided and adjustments were made for multiple comparisons. Source data are provided as a Source Data file. QFN, Quercetin-ferrum nanoparticles; QFN_{717}, QFN loaded with SR-717; nCSP, normal-Cholesterol cell membrane coated QFN_{717}; CSP, low-Cholesterol membrane coated QFN_{717}; nCISP, normal-Cholesterol cell membrane coated ICB agent and QFN_{717}; CISP, low-Cholesterol membrane coated ICB agent and QFN_{717}; CIP, low-Cholesterol membrane coated ICB agent and QFN.

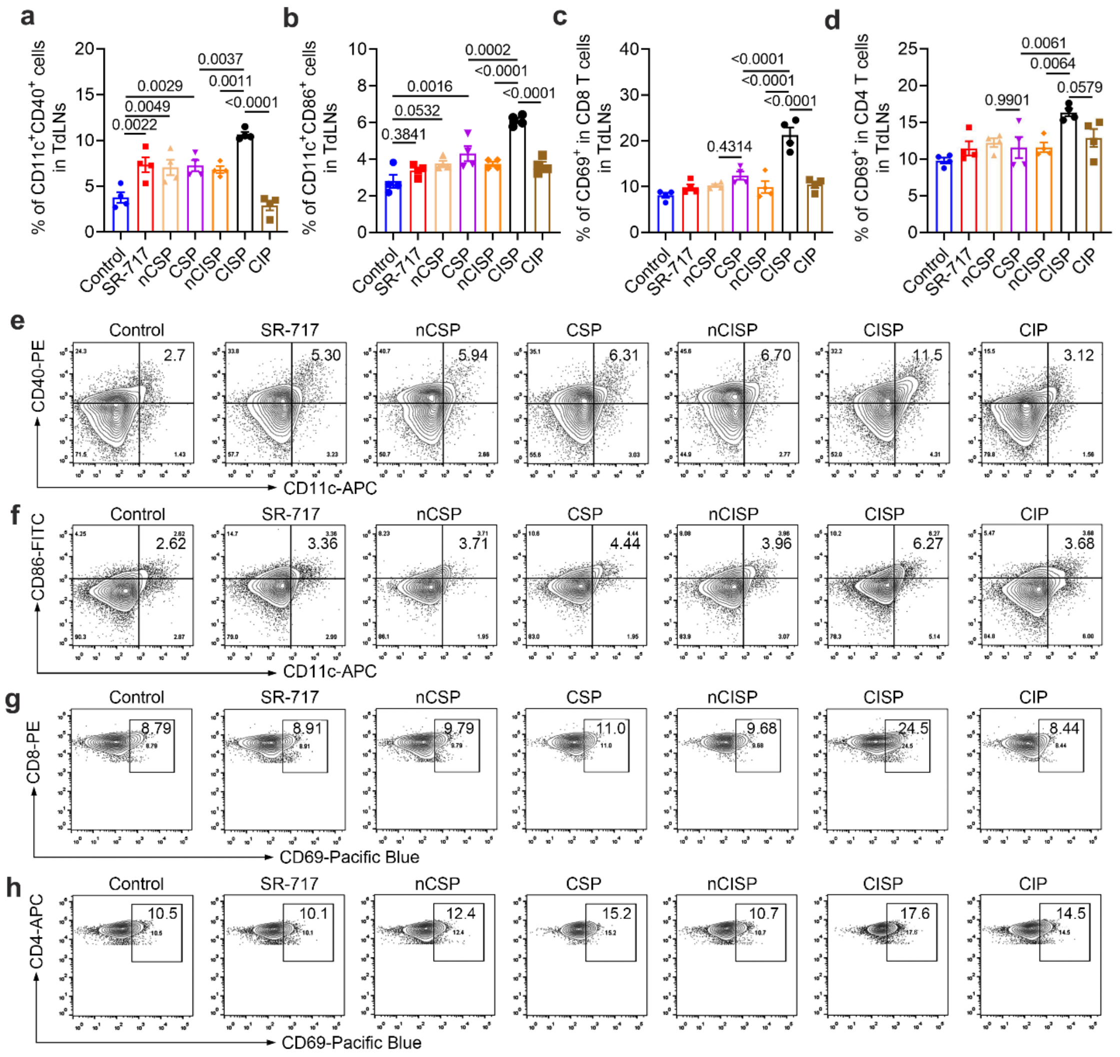

Supplementary Figure 39. DCs and T cells in the TdLNs in mice bearing MC38 tumors. **a**, **b** Flow-cytometric analysis of CD11c$^+$CD40$^+$ cells (**a**) and CD11$^+$CD86$^+$ cells (**b**) in the TdLNs at 18 days post tumor inoculation. Data represent mean ± SEM, n = 4 mice. **c**, **d** Flow-cytometric analysis of CD69$^+$ cells in CD3$^+$CD8$^+$ cells (**c**) and CD69$^+$ cells in CD3$^+$CD4$^+$ cells (**d**) in TdLNs at 18 days post tumor inoculation. Data represent mean ± SEM, n = 4 mice. **e**, **f** Representative flow cytometry dot plot of CD11c$^+$CD40$^+$ cells (**e**) and CD11$^+$CD86$^+$ cells (**f**) in the TdLNs. n = 4 mice. **g**, **h** Representative flow cytometry dot plot of CD69$^+$ cells in CD8 T cells (**g**) and CD69$^+$cells in CD4 T cells (**h**) in the TdLNs. n = 4 mice. Statistical significance was determined by one-way ANOVA test in **a**, **b**, **c**, **d**, and it was two-sided and adjustments were made for multiple comparisons. Source data are provided as a Source Data file. QFN, Quercetin-ferrum nanoparticles; QFN_{717}, QFN loaded with SR-717; nCSP, normal-Cholesterol cell membrane coated QFN_{717}; CSP, low-Cholesterol membrane coated QFN_{717}; nCISP, normal-Cholesterol cell membrane coated ICB agent and QFN_{717}; CISP, low-Cholesterol membrane coated ICB agent and QFN_{717}; CIP, low-Cholesterol membrane coated ICB agent and QFN; TdLNs, Tumor draining lymph nodes.

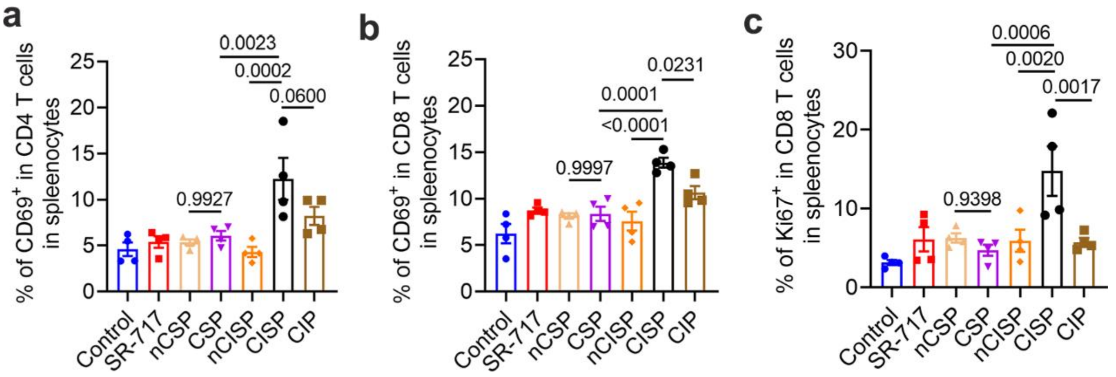

Supplementary Figure 40. Activated T cells in splenocytes in mice bearing MC38 tumors. a, **b**, **c** Flow-cytometric analysis of $CD69^+$ cells in $CD3^+CD4^+$ cells (**a**), $CD69^+$ cells in $CD3^+CD8^+$ cells (**b**), and $Ki67^+$ cells in $CD3^+CD8^+$ cell (**c**) in splenocytes at 18 days post tumor inoculation. Data represent mean ± SEM, n = 4 mice. Statistical significance was determined by one-way ANOVA test in **a**, **b**, **c**, and it was two-sided and adjustments were made for multiple comparisons. Source data are provided as a Source Data file. QFN, Quercetin-ferrum nanoparticles; QFN_{717}, QFN loaded with SR-717; nCSP, normal-Cholesterol cell membrane coated QFN_{717}; CSP, low-Cholesterol membrane coated QFN_{717}; nCISP, normal-Cholesterol cell membrane coated ICB agent and QFN_{717}; CISP, low-Cholesterol membrane coated ICB agent and QFN_{717}; CIP, low-Cholesterol membrane coated ICB agent and QFN.

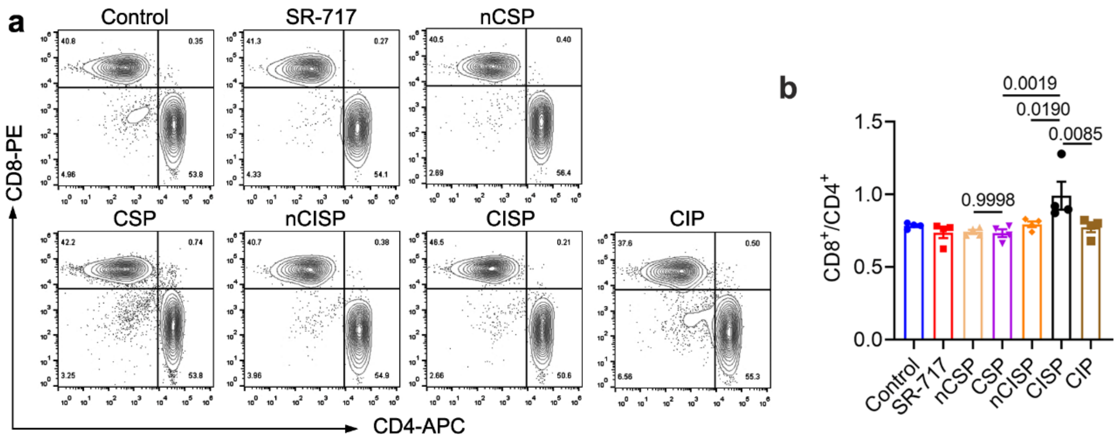

Supplementary Figure 41. Proportions of CD8$^+$ and CD4$^+$ T cells in TdLNs in mice bearing MC38 tumors. a, **b** Representative flow cytometry dot pot gated on CD3$^+$ T cells (**a**) and the proportions of CD8$^+$/CD4$^+$ (**b**). Data represent mean ± SEM, n = 4 mice. Statistical significance was determined by one-way ANOVA test in **b**, and it was two-sided and adjustments were made for multiple comparisons. Source data are provided as a Source Data file. QFN, Quercetin-ferrum nanoparticles; QFN_{717}, QFN loaded with SR-717; nCSP, normal-Cholesterol cell membrane coated QFN_{717}; CSP, low-Cholesterol membrane coated QFN_{717}; nCISP, normal-Cholesterol cell membrane coated ICB agent and QFN_{717}; CISP, low-Cholesterol membrane coated ICB agent and QFN_{717}; CIP, low-Cholesterol membrane coated ICB agent and QFN.

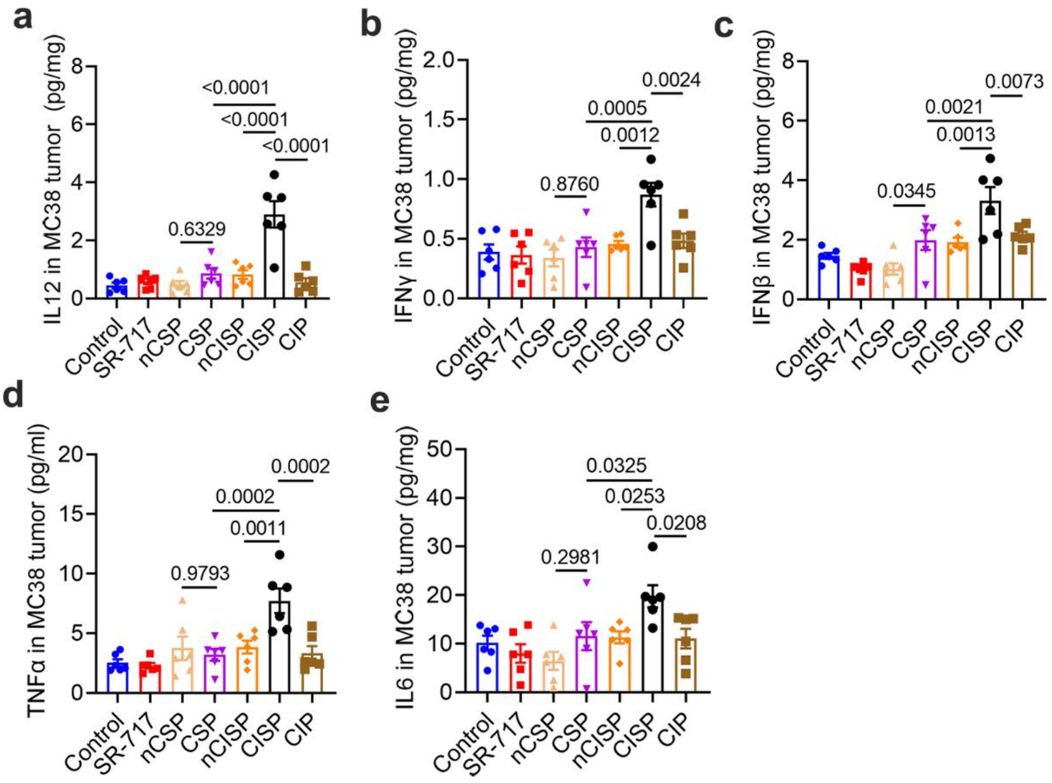

Supplementary Figure 42. Concentration of cytokines in MC38 tumors. a, b, c, d, e Concentration of IL12 (**a**), IFNγ (**b**), IFNβ (**c**), TNFα (**d**) and IL-6 (**e**) in MC38 tumors at 18 days post tumor inoculation. Data represent mean ± SEM, n = 6 mice. Statistical significance was determined by one-way ANOVA test in **a**, **b**, **c**, **d**, **e**, and it was two-sided and adjustments were made for multiple comparisons. Source data are provided as a Source Data file. QFN, Quercetin-ferrum nanoparticles; QFN_{717}, QFN loaded with SR-717; nCSP, normal-Cholesterol cell membrane coated QFN_{717}; CSP, low-Cholesterol membrane coated QFN_{717}; nCISP, normal-Cholesterol cell membrane coated ICB agent and QFN_{717}; CISP, low-Cholesterol membrane coated ICB agent and QFN_{717}; CIP, low-Cholesterol membrane coated ICB agent and QFN.

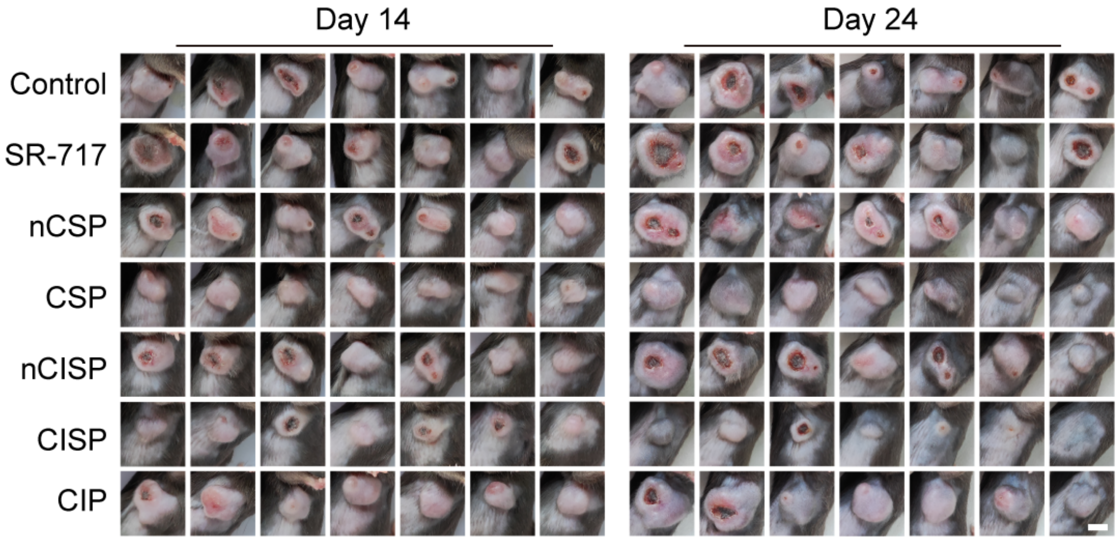

Supplementary Figure 43. Images of MC38 tumors at 14 days and 24 days post tumor inoculation. *n* = 7 mice. Scale bar = 0.5 cm. QFN, Quercetin-ferrum nanoparticles; QFN_{717}, QFN loaded with SR-717; nCSP, normal-Cholesterol cell membrane coated QFN_{717}; CSP, low-Cholesterol membrane coated QFN_{717}; nCISP, normal-Cholesterol cell membrane coated ICB agent and QFN_{717}; CISP, low-Cholesterol membrane coated ICB agent and QFN_{717}; CIP, low-Cholesterol membrane coated ICB agent and QFN.

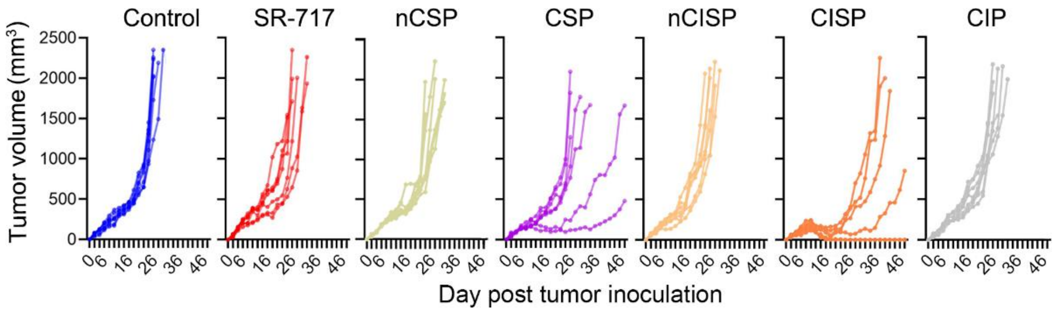

Supplementary Figure 44. Tumor growth curve of mice bearing MC38 tumors. *n* = 7 mice. Source data are provided as a Source Data file. QFN, Quercetin-ferrum nanoparticles; QFN_{717}, QFN loaded with SR-717; nCSP, normal-Cholesterol cell membrane coated QFN_{717}; CSP, low-Cholesterol membrane coated QFN_{717}; nCISP, normal-Cholesterol cell membrane coated ICB agent and QFN_{717}; CISP, low-Cholesterol membrane coated ICB agent and QFN_{717}; CIP, low-Cholesterol membrane coated ICB agent and QFN.

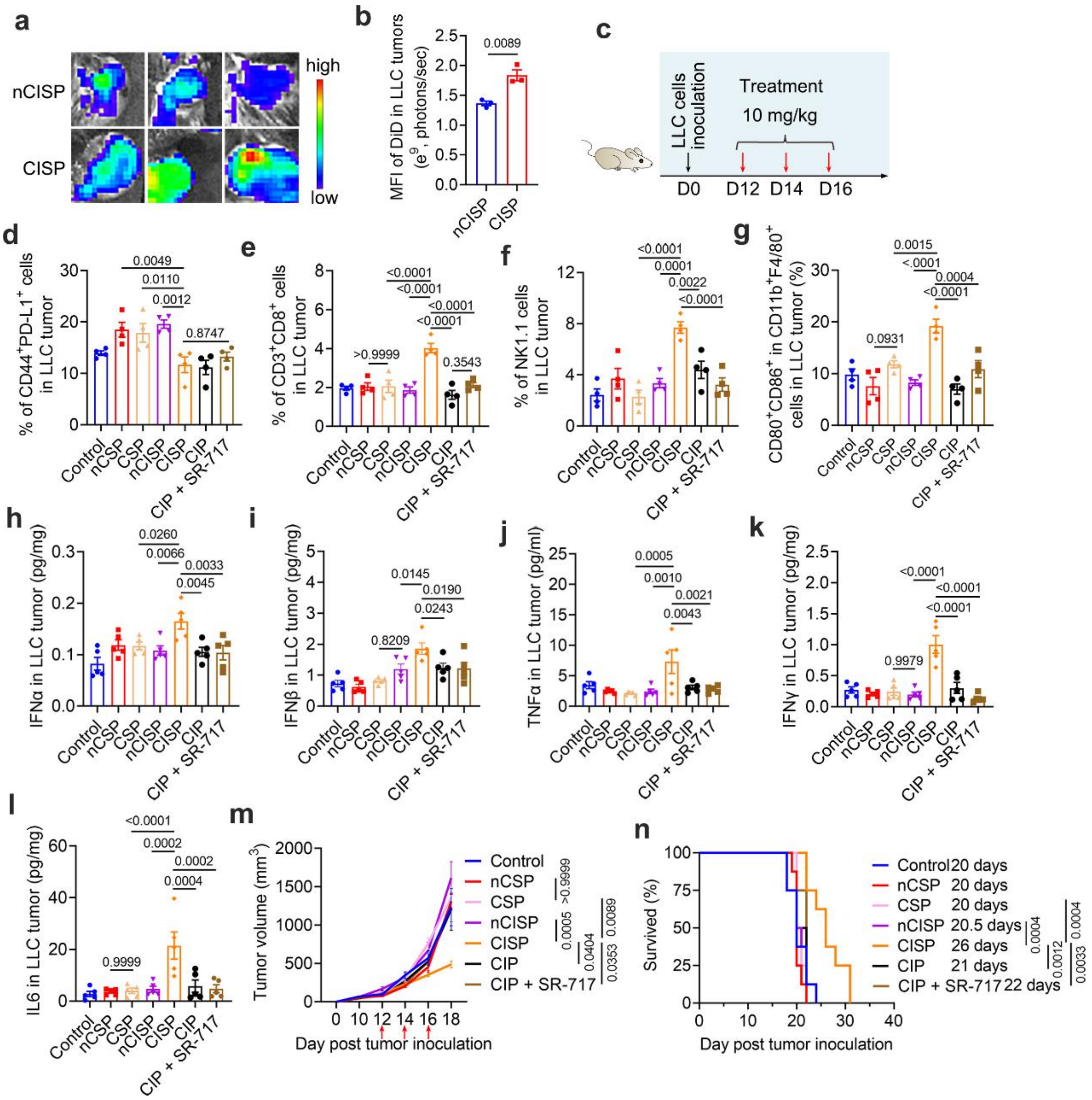

Supplementary Figure 45. The therapeutic effects of CISP in mice bearing LLC tumors. **a**, **b** Fluorescence images of LLC tumors at 6 h post injection of nCISP and CISP (**a**), and the mean fluorescence intensity (MFI) of DiD in tumors (**b**). Data represent mean ± SEM, n = 3 mice. **c** Schematic illustration showing the experiment process. Red arrows indicate the administration of nanomedicines. The dosage of SR-717 is 10 mg/kg. **d** Flow-cytometric analysis of $CD44^+PD\text{-}L1^+$ cells in LLC tumors at 18 days post tumor inoculation. Data represent mean ± SEM, n = 4 mice. **e** Flow-cytometric analysis of $CD3^+CD8^+$ cells in LLC tumors at 18 days post tumor inoculation. Data represent mean ± SEM, n = 4 mice. **f** Flow-cytometric analysis of $NK1.1^+$ cells in LLC tumors at 18 days post tumor inoculation. Data represent mean ± SEM, n = 4 mice. **g** Flow-cytometric analysis of $CD80^+CD86^+$ cells in $F4/80^+CD11b^+$ cells in LLC tumors at 18 days post tumor inoculation. Data represent mean ± SEM, n = 4 mice. **h**, **i**, **j**, **k**, **l** IFNα (**h**), IFNβ (**i**), TNFα (**j**), IFNγ (**k**) and IL6 (**l**) in LLC tumors at 18 days post tumor inoculation. Data represent mean ± SEM, n = 5 mice. **m** Tumor growth curve of mice bearing LLC tumors. Red arrows indicate administration of nanomedicines. Data represent mean ± SEM, n = 8 mice. **n** Survival curve of mice bearing LLC tumors. n = 8 mice. Student's two-sided t test was used for the statistical

analysis in **b**. Statistical significance was determined by one-way ANOVA test in **d**, **e**, **f**, **g**, **h**, **i**, **j**, **k**, **l**, and it was two-sided and adjustments were made for multiple comparisons. Statistical significance was determined by two-way ANOVA test in **m**, and it was two-sided and adjustments were made for multiple comparisons. Survival was measured using the Kaplan-Meier method and statistical significance was calculated by log-rank test in **n**. Source data are provided as a Source Data file. QFN, Quercetin-ferrum nanoparticles; QFN_{717}, QFN loaded with SR-717; nCSP, normal-Cholesterol cell membrane coated QFN_{717}; CSP, low-Cholesterol membrane coated QFN_{717}; nCISP, normal-Cholesterol cell membrane coated ICB agent and QFN_{717}; CISP, low-Cholesterol membrane coated ICB agent and QFN_{717}; CIP, low-Cholesterol membrane coated ICB agent and QFN.

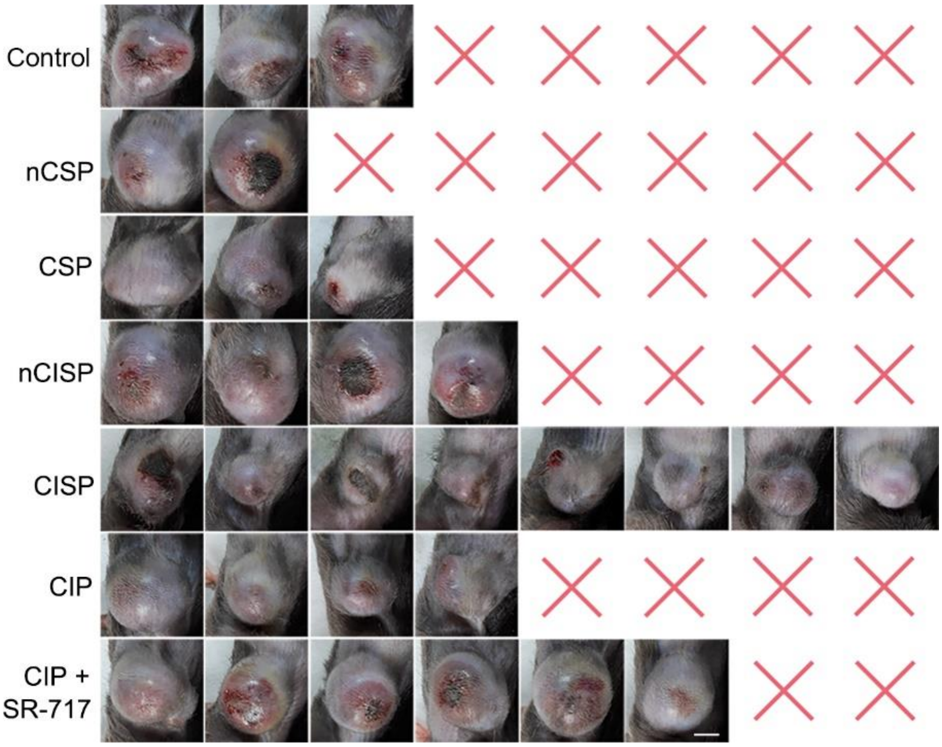

Supplementary Figure 46. Images of LLC tumors in mice at 20 days post tumor inoculation. Red cross line represents the dead mouse. $n = 8$ mice. Scale bar = 0.5 cm. QFN, Quercetin-ferrum nanoparticles; QFN_{717}, QFN loaded with SR-717; nCSP, normal-Cholesterol cell membrane coated QFN_{717}; CSP, low-Cholesterol membrane coated QFN_{717}; nCISP, normal-Cholesterol cell membrane coated ICB agent and QFN_{717}; CISP, low-Cholesterol membrane coated ICB agent and QFN_{717}; CIP, low-Cholesterol membrane coated ICB agent and QFN.

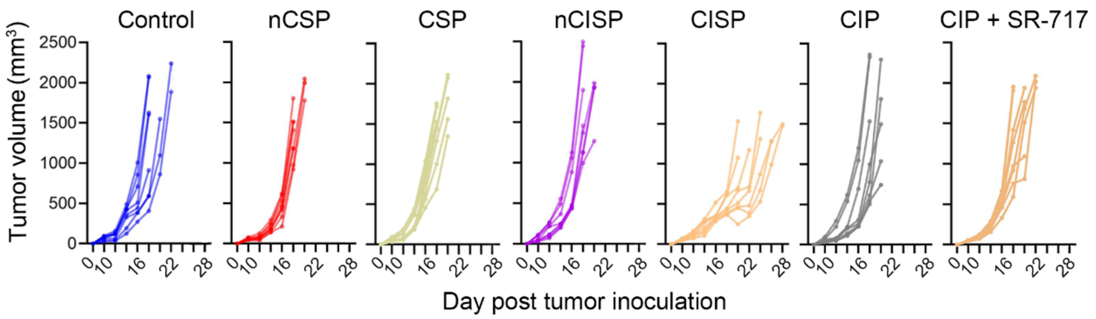

Supplementary Figure 47. Tumor growth curve of mice bearing LLC tumors. n = 8 mice. Source data are provided as a Source Data file. QFN, Quercetin-ferrum nanoparticles; QFN_{717}, QFN loaded with SR-717; nCSP, normal-Cholesterol cell membrane coated QFN_{717}; CSP, low-Cholesterol membrane coated QFN_{717}; nCISP, normal-Cholesterol cell membrane coated ICB agent and QFN_{717}; CISP, low-Cholesterol membrane coated ICB agent and QFN_{717}; CIP, low-Cholesterol membrane coated ICB agent and QFN.

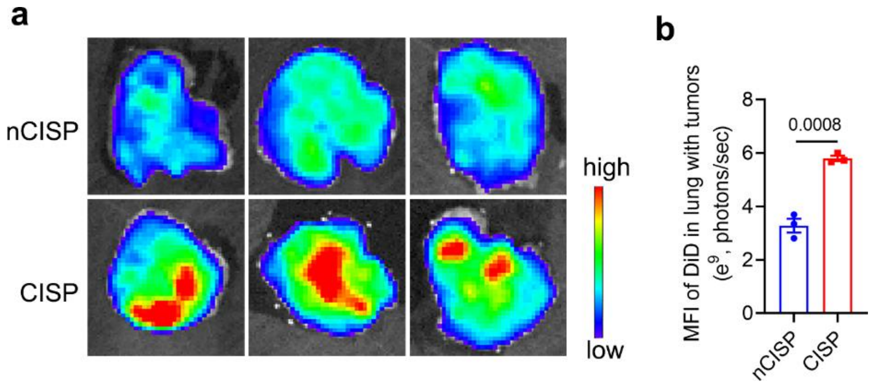

Supplementary Figure 48. Fluorescence images of lung with metastatic melanoma. **a**, **b** Fluorescence images of lung at 6 h post injection of nCISP and CISP (**a**), and the mean fluorescence intensity (MFI) of DiD in lung (**b**). Data represent mean ± SEM, n = 3 mice. Student's two-sided t test was used for the statistical analysis in **b**. Source data are provided as a Source Data file. QFN, Quercetin-ferrum nanoparticles; QFN_{717}, QFN loaded with SR-717; nCISP, normal-Cholesterol cell membrane coated ICB agent and QFN_{717}; CISP, low-Cholesterol membrane coated ICB agent and QFN_{717}.

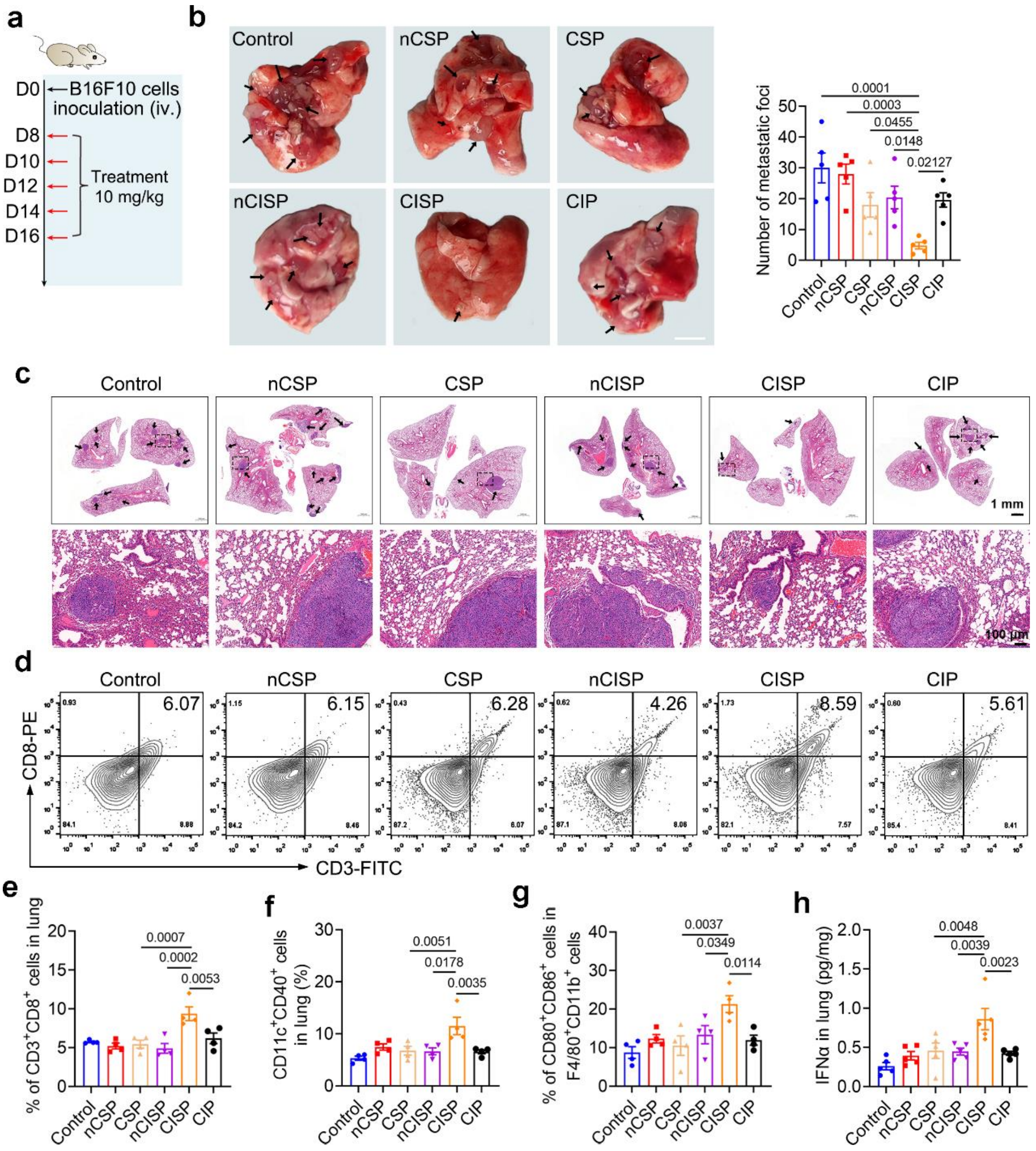

Supplementary Figure 49. The therapeutic effects of CISP in mice bearing metastatic melanoma. a Schematic illustration of the experiment process. The dosage of SR-717 is 10 mg/kg. Red arrows indicate the administration of nanomedicines. **b** Representative images of lung and the number of pulmonary metastasis foci at 18 days post B16F10 injection. Metastasis foci is pointed by arrow. Scale bar = 0.3 cm. Data represent mean ± SEM, *n* = 5 mice. **c** Representative images of H&E staining of lung sections in mice. Metastasis foci is pointed by arrow. *n* = 5 mice. **d**, **e** Flow-cytometric analysis of $CD3^+CD8^+$ cell in lung at 18 days post B16F10 injection. Data represent mean ± SEM, *n* = 4 mice. **f** Flow-cytometric analysis of $CD11c^+CD40^+$ cells in lung at 18 days post B16F10 injection. Data represent mean ± SEM, *n* = 4 mice. **g** Flow-cytometric analysis of $CD80^+CD86^+$ cells in $F4/80^+CD11b^+$ cells in lung at 18 days post B16F10 injection. Data represent mean ± SEM, *n* = 4 mice. **h** IFNα in lung at 18 days post B16F10 injection. Data represent mean ± SEM, *n* = 5 mice. Statistical significance was determined by one-way ANOVA test in **b**, **e**, **f**, **g**, **h**, and it was two-sided and

adjustments were made for multiple comparisons. Source data are provided as a Source Data file. QFN, Quercetin-ferrum nanoparticles; QFN_{717}, QFN loaded with SR-717; nCSP, normal-Cholesterol cell membrane coated QFN_{717}; CSP, low-Cholesterol membrane coated QFN_{717}; nCISP, normal-Cholesterol cell membrane coated ICB agent and QFN_{717}; CISP, low-Cholesterol membrane coated ICB agent and QFN_{717}; CIP, low-Cholesterol membrane coated ICB agent and QFN.

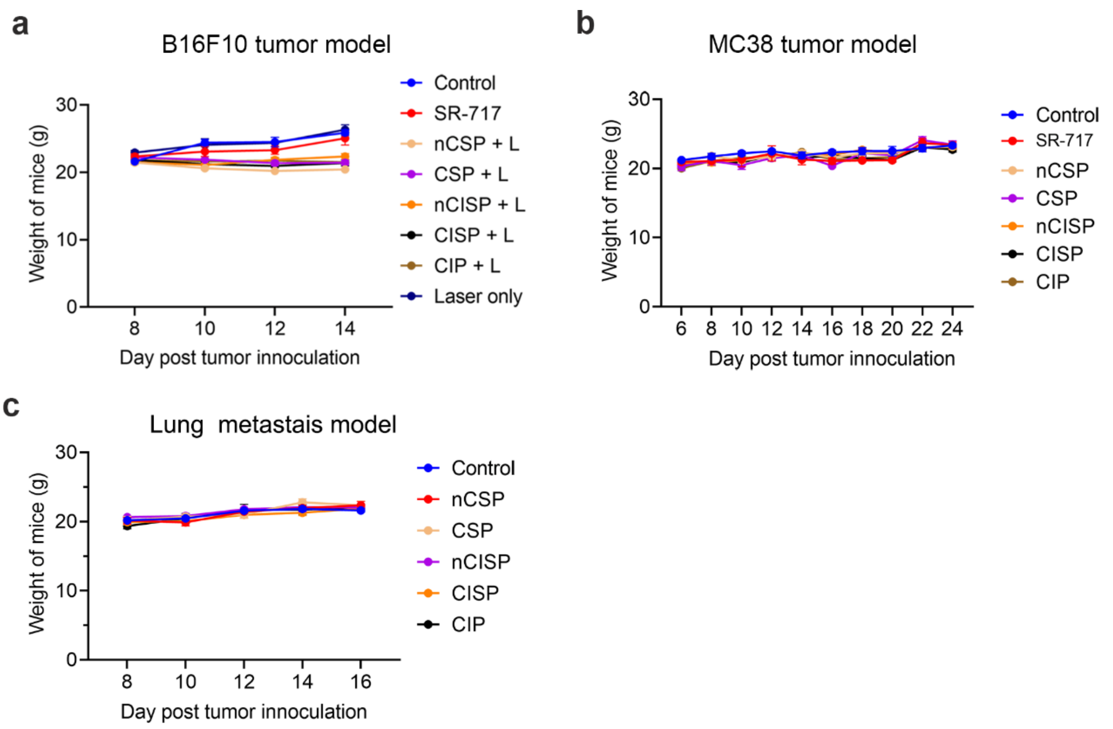

Supplementary Figure 50. Body weight of mice. a Body weight of mice bearing B16F10 melanoma receiving the indicated treatment. Data represent mean ± SEM, *n* = 5 mice. **b** Body weight of mice bearing MC38 tumors receiving the indicated treatment. Data represent mean ± SEM, *n* = 5 mice. **c** Body weight of mice bearing metastatic melanoma receiving the indicated treatment. Data represent mean ± SEM, *n* = 5 mice. Source data are provided as a Source Data file. L, laser; QFN, Quercetin-ferrum nanoparticles; QFN_{717}, QFN loaded with SR-717; nCSP, normal-Cholesterol cell membrane coated QFN_{717}; CSP, low-Cholesterol membrane coated QFN_{717}; nCISP, normal-Cholesterol cell membrane coated ICB agent and QFN_{717}; CISP, low-Cholesterol membrane coated ICB agent and QFN_{717}; CIP, low-Cholesterol membrane coated ICB agent and QFN.

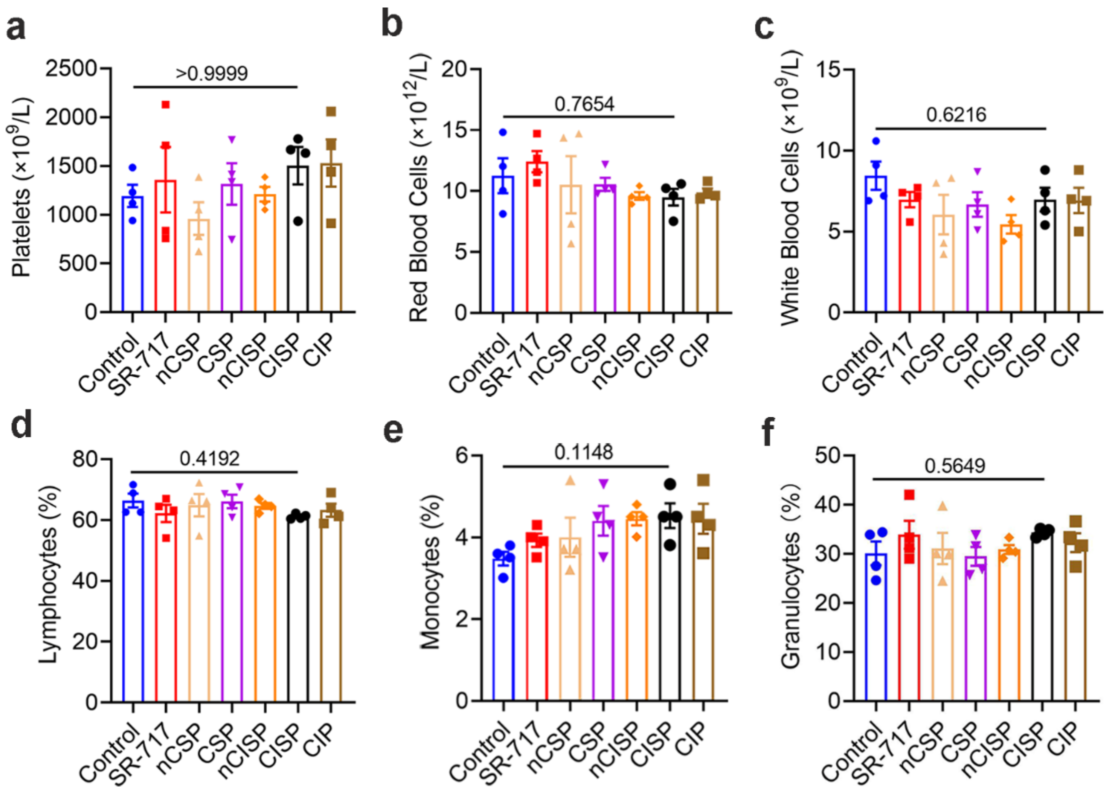

Supplementary Figure 51. Blood routine of mice bearing MC38 tumors at 18 days post tumor inoculation. a Platelets in mice receiving the indicated treatment. Data represent mean ± SEM, n = 4 mice. **b** Red blood cells in mice receiving the indicated treatment. Data represent mean ± SEM, n = 4 mice. **c** White blood cells in mice receiving the indicated treatment. Data represent mean ± SEM, n = 4 mice. **d** Lymphocytes in mice receiving the indicated treatment. Data represent mean ± SEM, n = 4 mice. **e** Monocytes in mice receiving the indicated treatment. Data represent mean ± SEM, n = 4 mice. **f** Granulocytes in mice receiving the indicated treatment. Data represent mean ± SEM, n = 4 mice. Statistical significance was determined by one-way ANOVA test in **a**, **b**, **c**, **d**, **e**, **f**, and it was two-sided and adjustments were made for multiple comparisons. Source data are provided as a Source Data file. QFN, Quercetin-ferrum nanoparticles; QFN_{717}, QFN loaded with SR-717; nCSP, normal-Cholesterol cell membrane coated QFN_{717}; CSP, low-Cholesterol membrane coated QFN_{717}; nCISP, normal-Cholesterol cell membrane coated ICB agent and QFN_{717}; CISP, low-Cholesterol membrane coated ICB agent and QFN_{717}; CIP, low-Cholesterol membrane coated ICB agent and QFN.

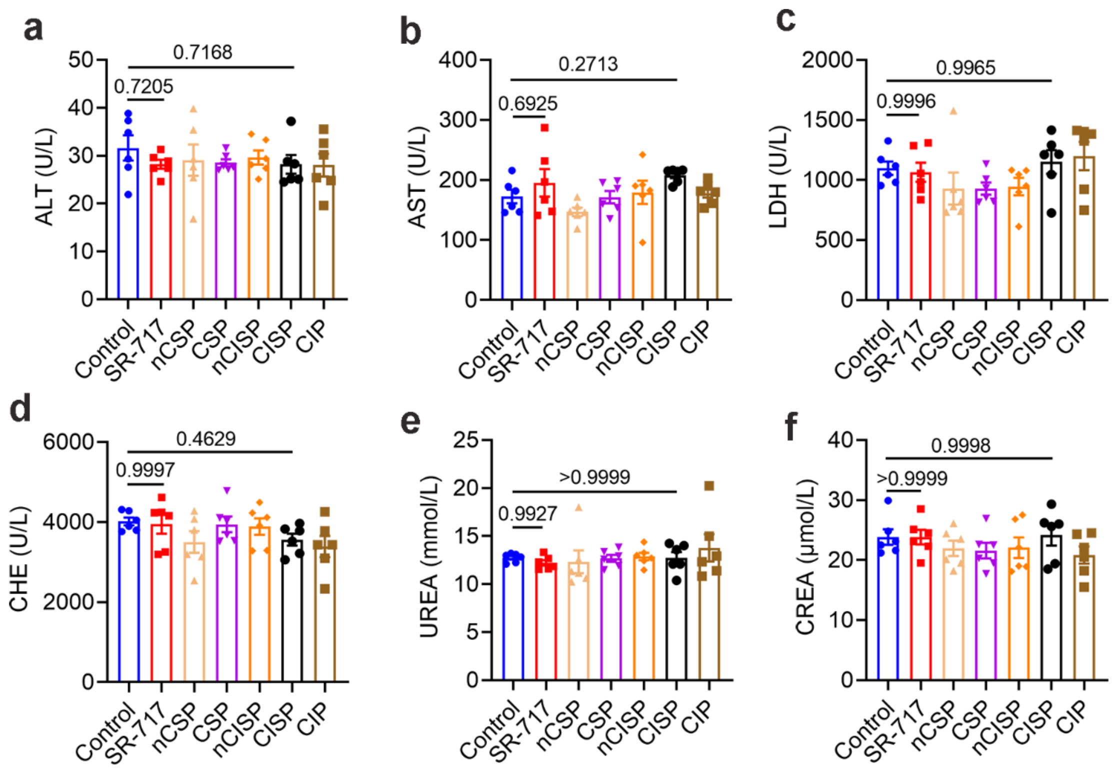

Supplementary Figure 52. Blood biochemistry of mice bearing MC38 tumors at 18 days post tumor inoculation. **a** The serum level of ALT in mice receiving the indicated treatment. Data represent mean ± SEM, *n* = 6 mice. **b** The serum level of AST in mice receiving the indicated treatment. Data represent mean ± SEM, *n* = 6 mice. **c** The serum level of LDH in mice receiving the indicated treatment. Data represent mean ± SEM, *n* = 6 mice. **d** The serum level of CHE in mice receiving the indicated treatment. Data represent mean ± SEM, *n* = 6 mice. **e** The serum level of UREA in mice receiving the indicated treatment. Data represent mean ± SEM, *n* = 6 mice. **f** The serum level of CREA in mice receiving the indicated treatment. Data represent mean ± SEM, *n* = 6 mice. Statistical significance was determined by one-way ANOVA test in **a**, **b**, **c**, **d**, **e**, **f**, and it was two-sided and adjustments were made for multiple comparisons. Source data are provided as a Source Data file. ALT, alanine aminotransferase; AST, aspartate aminotransferase; LDH, lactic dehydrogenase; CHE, Cholinesterase; CREA, Creatine. QFN, Quercetin-ferrum nanoparticles; QFN_{717}, QFN loaded with SR-717; nCSP, normal-Cholesterol cell membrane coated QFN_{717}; CSP, low-Cholesterol membrane coated QFN_{717}; nCISP, normal-Cholesterol cell membrane coated ICB agent and QFN_{717}; CISP, low-Cholesterol membrane coated ICB agent and QFN_{717}; CIP, low-Cholesterol membrane coated ICB agent and QFN.

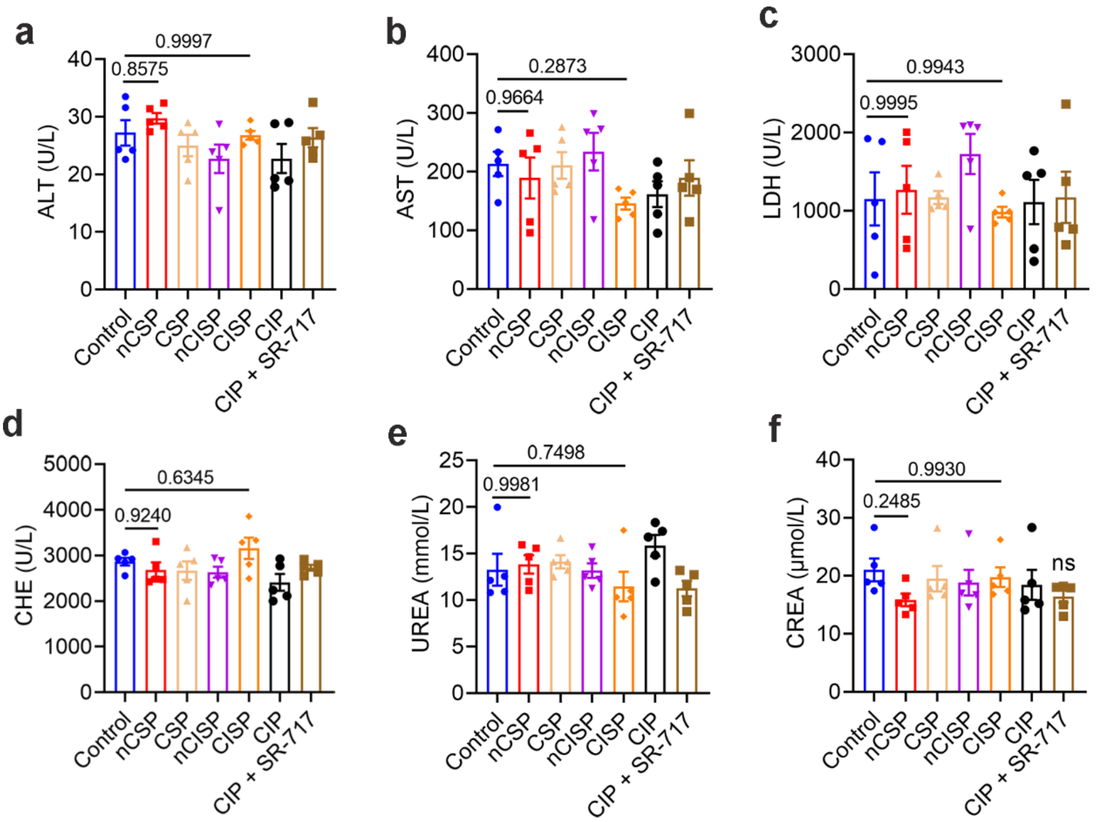

Supplementary Figure 53. Blood biochemistry of mice bearing LLC tumor at 18 days post tumor inoculation. **a** The serum level of ALT in mice receiving the indicated treatment. Data represent mean ± SEM, *n* = 5 mice. **b** The serum level of AST in mice receiving the indicated treatment. Data represent mean ± SEM, *n* = 5 mice. **c** The serum level of LDH in mice receiving the indicated treatment. Data represent mean ± SEM, *n* = 5 mice. **d** The serum level of CHE in mice receiving the indicated treatment. Data represent mean ± SEM, *n* = 5 mice. **e** The serum level of UREA in mice receiving the indicated treatment. Data represent mean ± SEM, *n* = 5 mice. **f** The serum level of CREA in mice receiving the indicated treatment. Data represent mean ± SEM, *n* = 5 mice. Statistical significance was determined by one-way ANOVA test in **a**, **b**, **c**, **d**, **e**, **f**, and it was two-sided and adjustments were made for multiple comparisons. Source data are provided as a Source Data file. ALT, alanine aminotransferase; AST, aspartate aminotransferase; LDH, lactic dehydrogenase; CHE, Cholinesterase; CREA, Creatine. QFN, Quercetin-ferrum nanoparticles; QFN_{717}, QFN loaded with SR-717; nCSP, normal-Cholesterol cell membrane coated QFN_{717}; CSP, low-Cholesterol membrane coated QFN_{717}; nCISP, normal-Cholesterol cell membrane coated ICB agent and QFN_{717}; CISP, low-Cholesterol membrane coated ICB agent and QFN_{717}; CIP, low-Cholesterol membrane coated ICB agent and QFN.

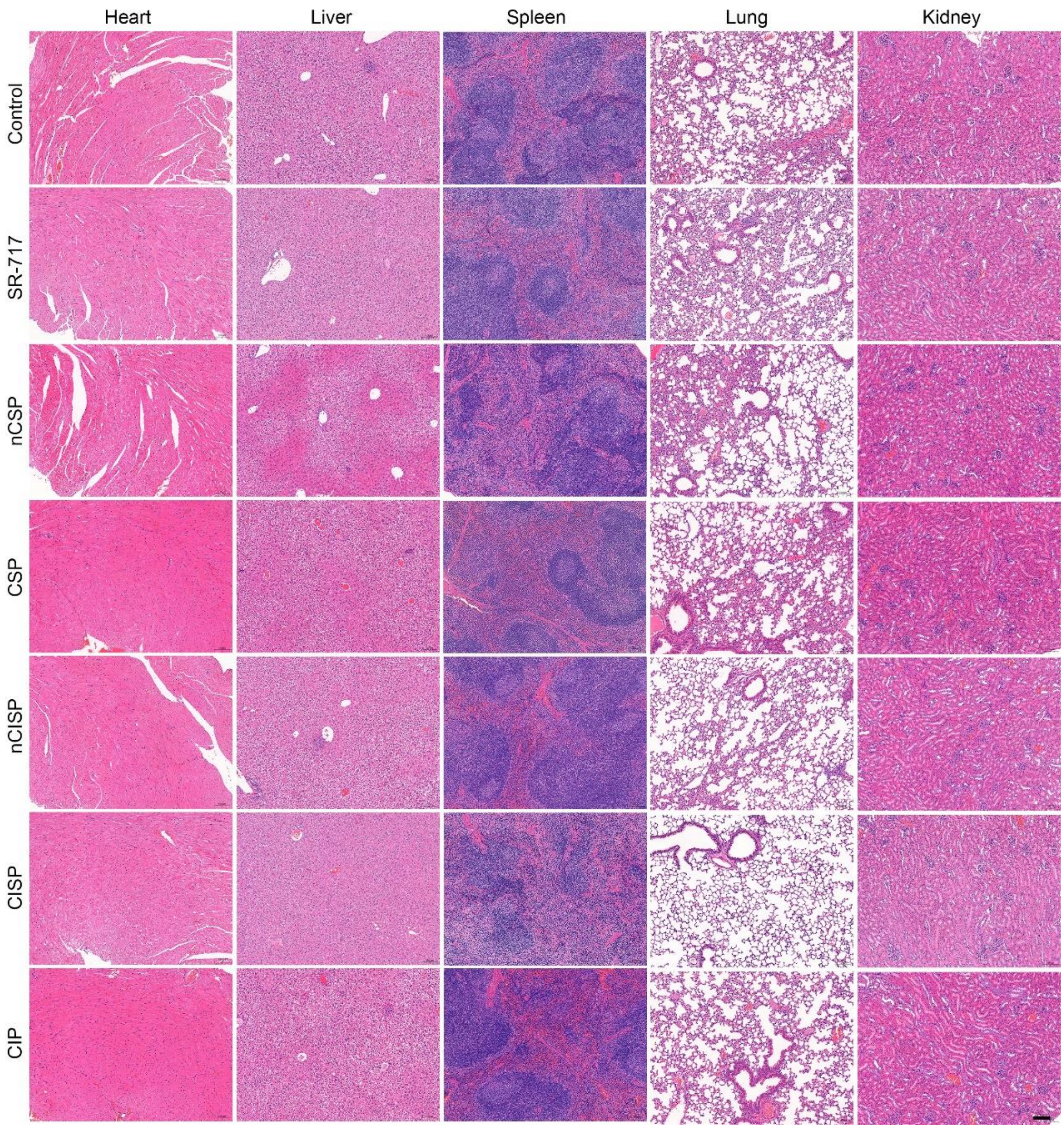

Supplementary Figure 54. H&E staining of major organs in mice bearing MC38 tumors. Mice bearing MC38 tumors receiving the indicated treatment were sacrificed at 18 days post tumor inoculation. Scale bar = 100 μm. n = 3 mice. QFN, Quercetin-ferrum nanoparticles; QFN_{717}, QFN loaded with SR-717; nCSP, normal-Cholesterol cell membrane coated QFN_{717}; CSP, low-Cholesterol membrane coated QFN_{717}; nCISP, normal-Cholesterol cell membrane coated ICB agent and QFN_{717}; CISP, low-Cholesterol membrane coated ICB agent and QFN_{717}; CIP, low-Cholesterol membrane coated ICB agent and QFN.

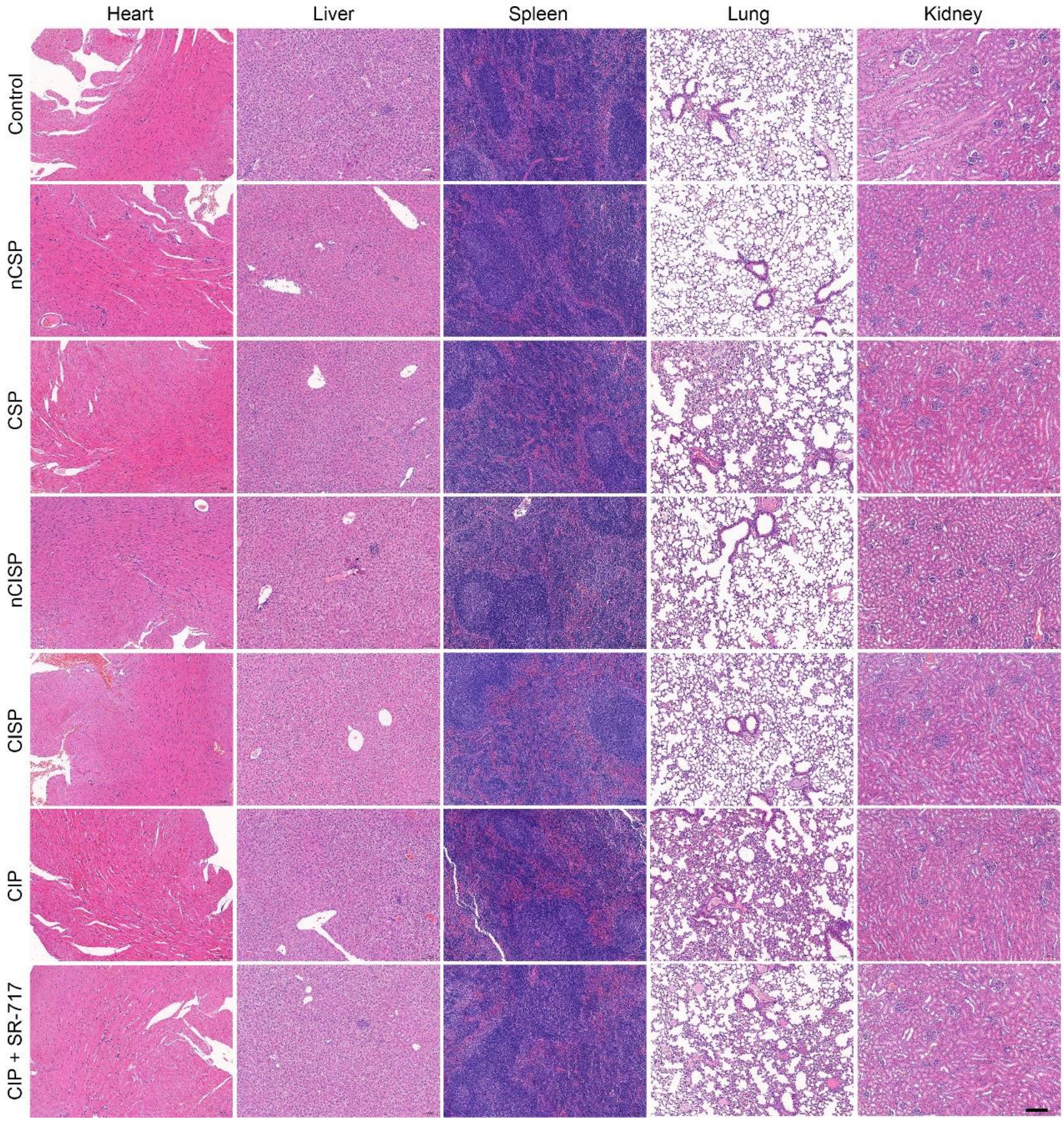

Supplementary Figure 55. H&E staining of major organs in mice bearing LLC tumors. Mice bearing LLC tumors receiving the indicated treatment were sacrificed at 18 days post tumor inoculation. Scale bar = 100 µm. *n* = 3 mice. QFN, Quercetin-ferrum nanoparticles; QFN_{717}, QFN loaded with SR-717; nCSP, normal-Cholesterol cell membrane coated QFN_{717}; CSP, low-Cholesterol membrane coated QFN_{717}; nCISP, normal-Cholesterol cell membrane coated ICB agent and QFN_{717}; CISP, low-Cholesterol membrane coated ICB agent and QFN_{717}; CIP, low-Cholesterol membrane coated ICB agent and QFN.

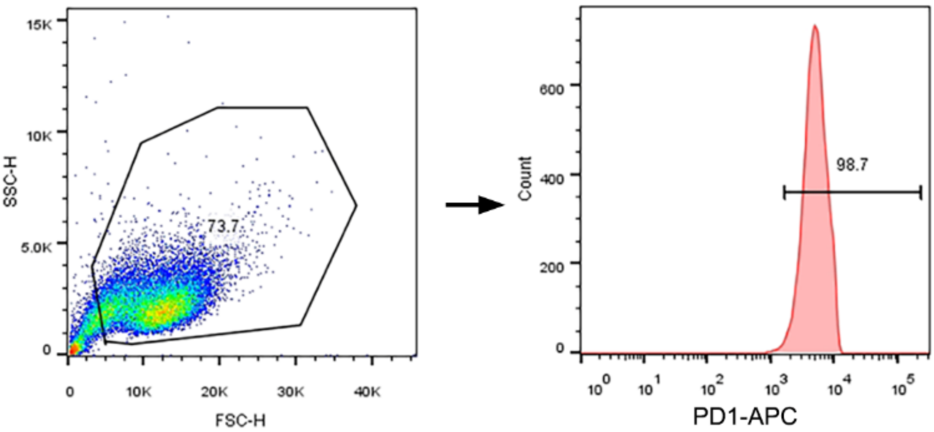

Supplementary Figure 56. Gating strategy to quantify PD-1 level on the membrane of CTLL2-PD1 cells (Fig. 1b).

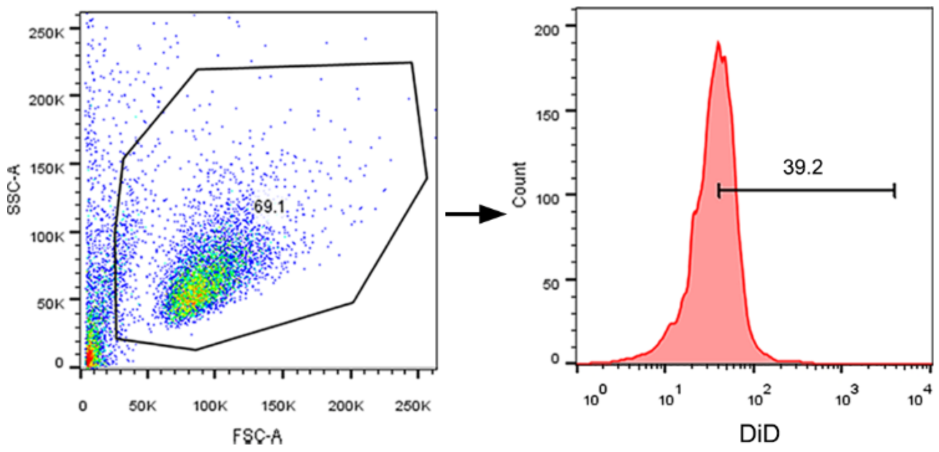

Supplementary Figure 57. Gating strategy to quantify the uptake of nanoparticles by cells (Fig. 2a, b, Supplementary Figure 18a-d).

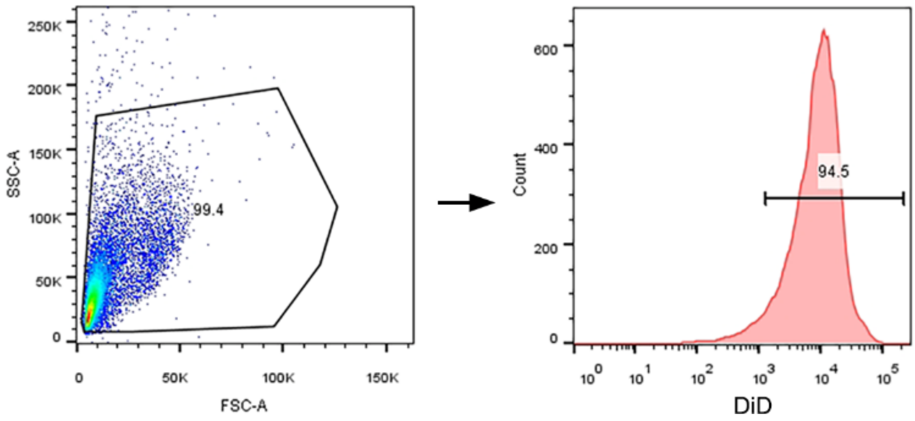

Supplementary Figure 58. Gating strategy to quantify the uptake of nanoparticles by lymphocytes, neutrophils and monocytes in the blood (Fig. 4j-o and Fig. 5f).

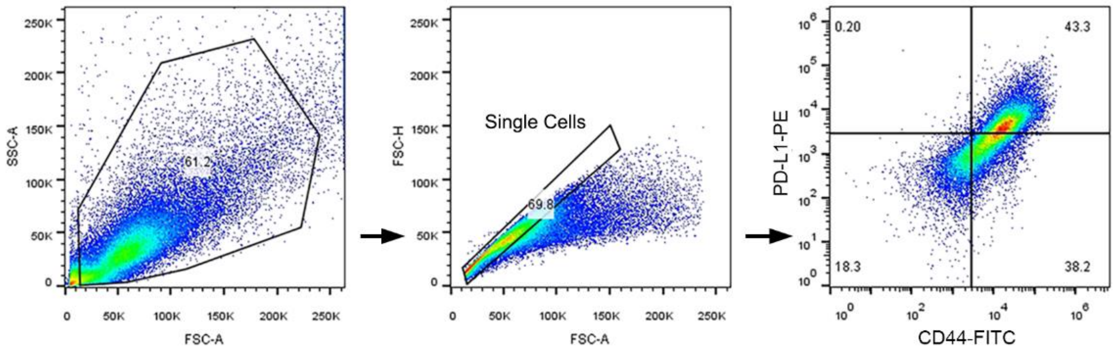

Supplementary Figure 59. Gating strategy to quantify CD44$^+$PD-L1$^+$ cells in tumors (Fig. 6c, Fig. 7b and Supplementary Figure 45d).

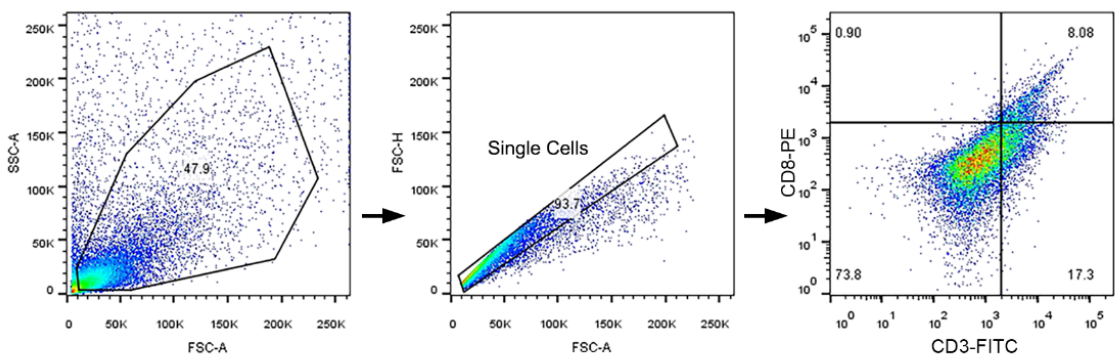

Supplementary Figure 60. Gating strategy to quantify $CD3^+CD8^+$ cells in tumors (Fig. 6d, Fig. 7c and Supplementary Figure 45e).

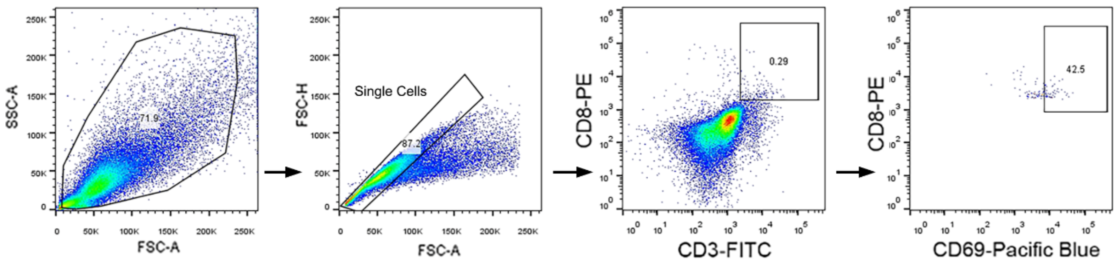

Supplementary Figure 61. Gating strategy to quantify CD69$^+$ cells in CD8 T cells in tumors (Fig. 6e and Fig. 7d).

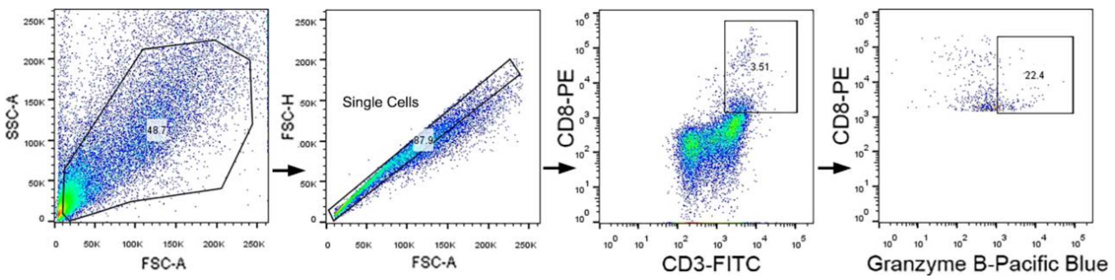

Supplementary Figure 62. Gating strategy to quantify Granzyme B$^+$ cells in CD8 T cells in tumors (Fig. 6f and Fig. 7e).

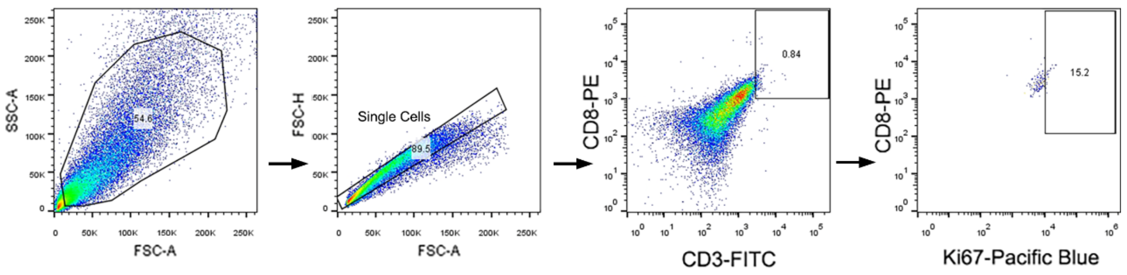

Supplementary Figure 63. Gating strategy to quantify Ki67$^+$ cells in CD8 T cells in tumors (Fig. 6g and Fig. 7f).

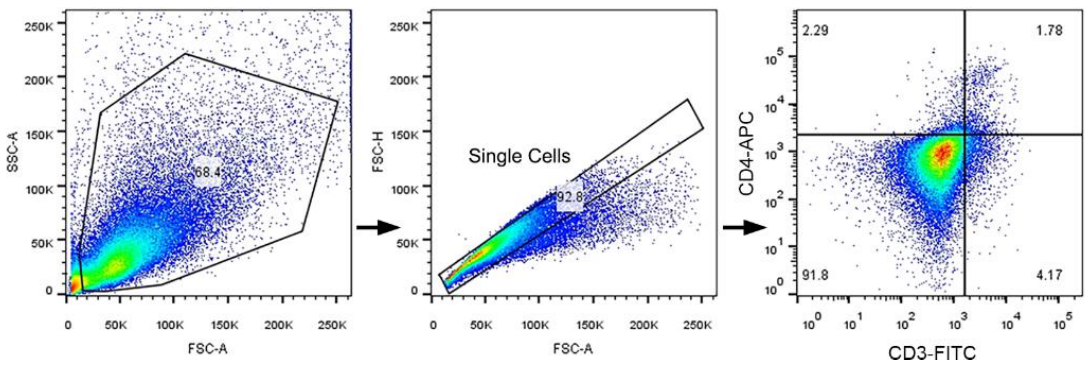

Supplementary Figure 64. Gating strategy to quantify $CD3^{+}CD4^{+}$ cells in tumors (Fig. 6h and Fig. 7g).

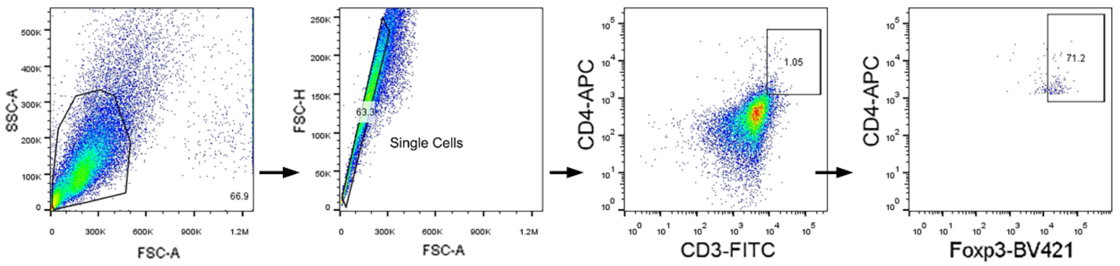

Supplementary Figure 65. Gating strategy to quantify Foxp3^{+} cells in CD4 T cells in tumors (Fig. 6i and Fig. 7h).

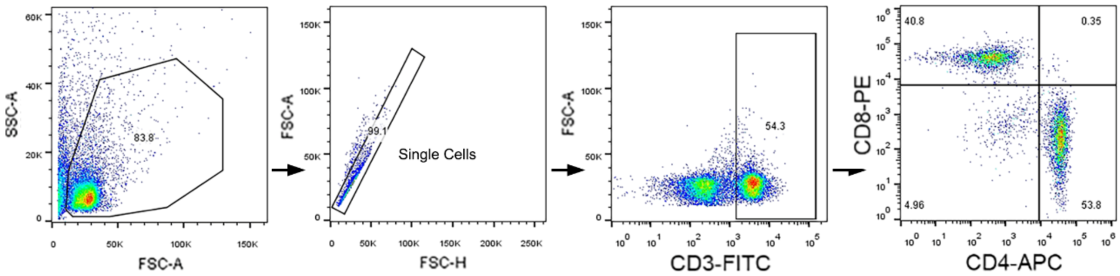

Supplementary Figure 66. Gating strategy to quantify the proportions of $CD8^+$ and $CD4^+$ T cells in TdLNs in mice (Supplementary Figure 41a, b).

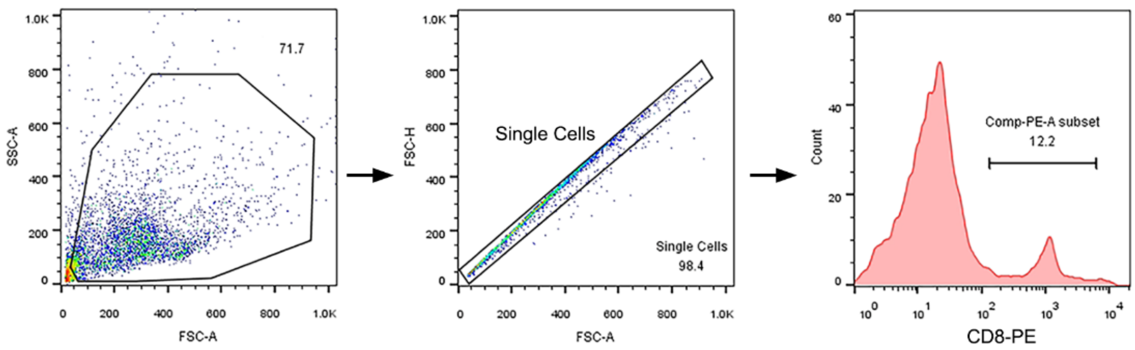

Supplementary Figure 67. Gating strategy to quantify $CD8^+$ T cells in splenocytes and purified splenocytes (Supplementary Figure 8a).

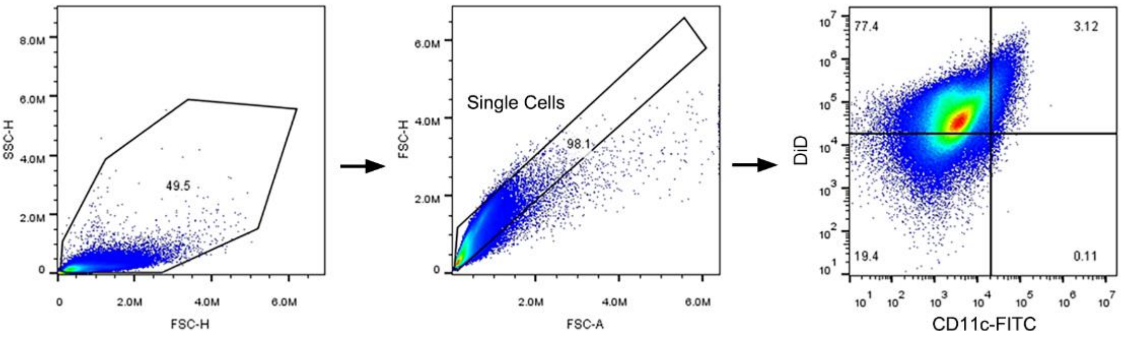

Supplementary Figure 68. Gating strategy to quantify the uptake of nanoparticles by DCs in tumors (Supplementary Figure 11a, b).

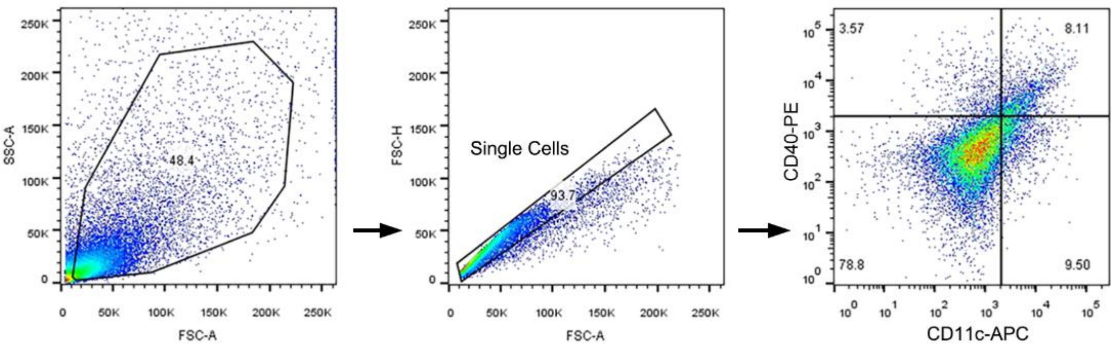

Supplementary Figure 69. Gating strategy to quantify CD11c^{+}CD40^{+} cells in tumors (Supplementary Figure 30a).

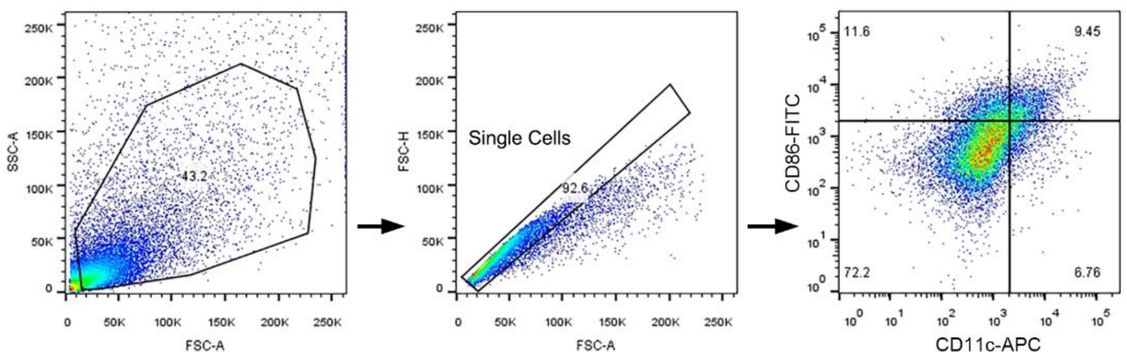

Supplementary Figure 70. Gating strategy to quantify $CD11c^{+}CD86^{+}$ cells in tumors (Supplementary Figure 30b and Supplementary Figure 38c).

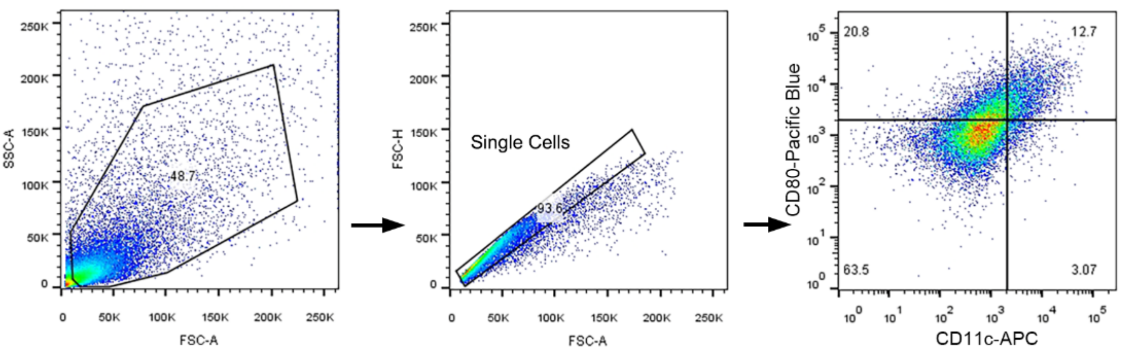

Supplementary Figure 71. Gating strategy to quantify CD11c$^+$CD80$^+$ cells in tumors (Supplementary Figure 30c).

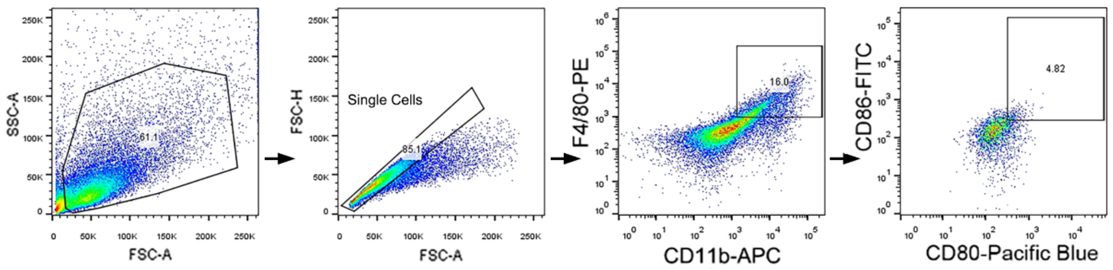

Supplementary Figure 72. Gating strategy to quantify $CD80^+CD86^+$ cells in $CD11b^+F4/80^+$ cells in tumors (Supplementary Figure 31a, Supplementary Figure 38a, Supplementary Figure 45g and Supplementary Figure 49g).

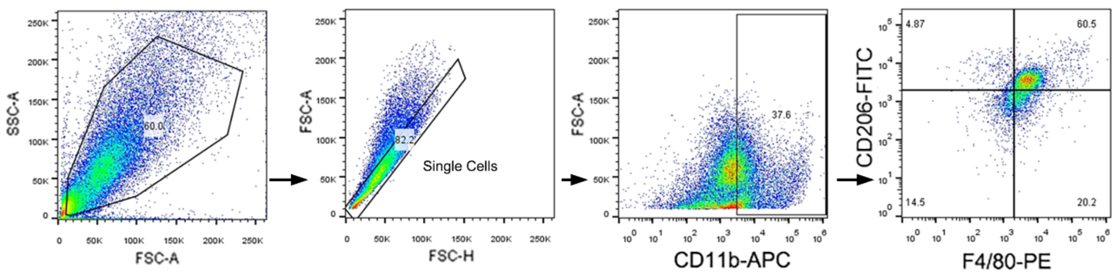

Supplementary Figure 73. Gating strategy to quantify $CD206^{+}F4/80^{+}$ cells in $CD11b^{+}$ cells in tumors (Supplementary Figure 31b).

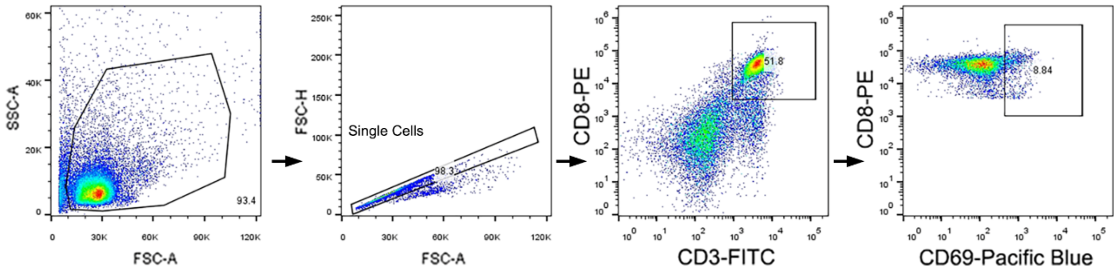

Supplementary Figure 74. Gating strategy to quantify CD69$^+$ cells in CD8 T cells in TdLNs (Supplementary Figure 33a, Supplementary Figure 39c).

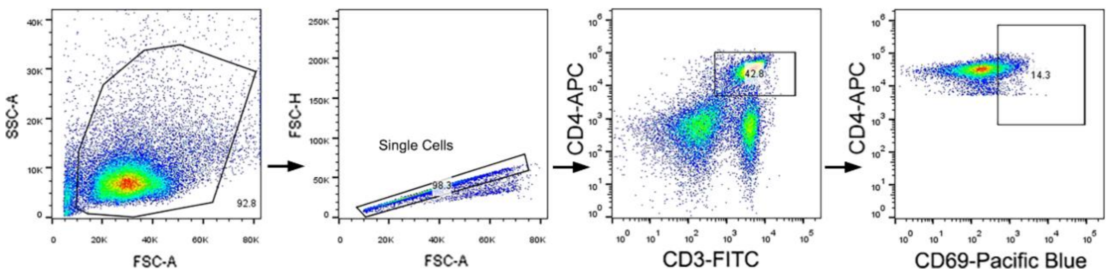

Supplementary Figure 75. Gating strategy to quantify $CD69^+$ cells in CD4 T cells in TdLNs (Supplementary Figure 33b, Supplementary Figure 39d).

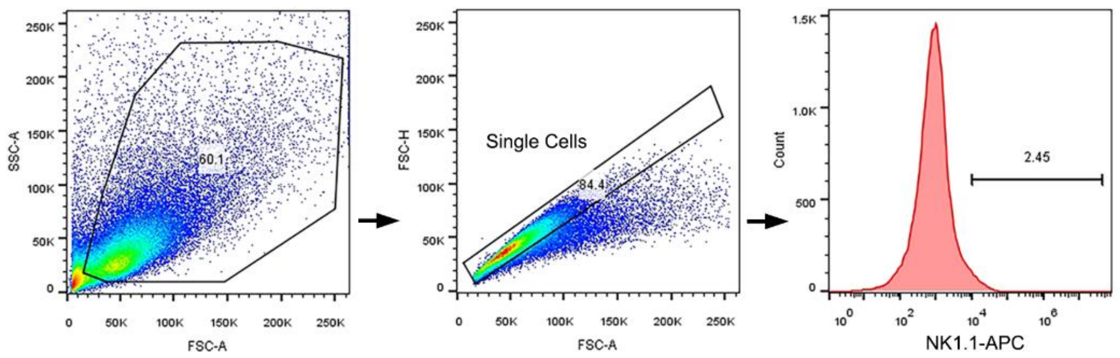

Supplementary Figure 76. Gating strategy to quantify NK1.1$^+$ cells in tumors (Supplementary Figure 38b, Supplementary Figure 45f).

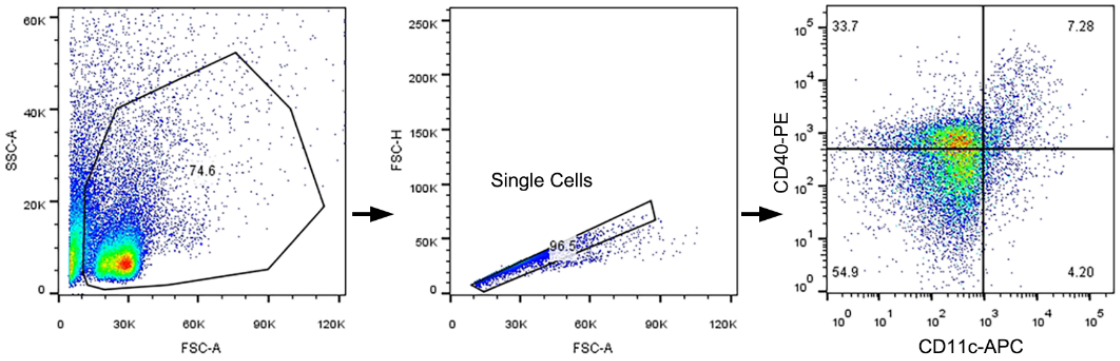

Supplementary Figure 77. Gating strategy to quantify CD11c^{+}CD40^{+} cells in TdLNs (Supplementary Figure 39a).

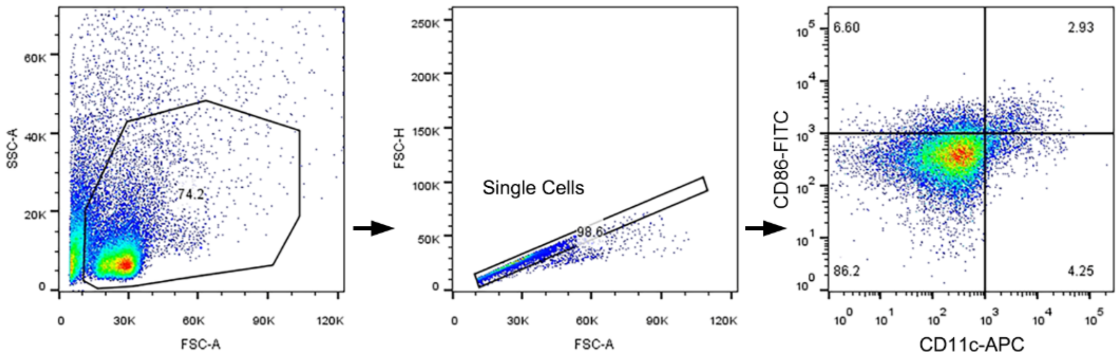

Supplementary Figure 78. Gating strategy to quantify CD11c$^+$CD86$^+$ cells in TdLNs (Supplementary Figure 39b).

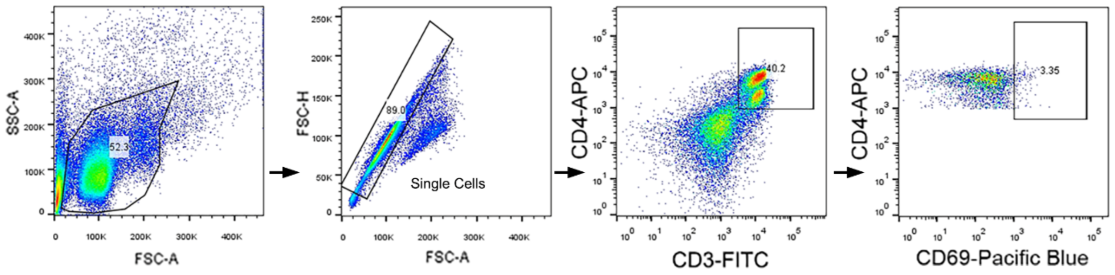

Supplementary Figure 79. Gating strategy to quantify CD69$^+$ cells in CD4 T cells in splenocytes (Supplementary Figure 40a).

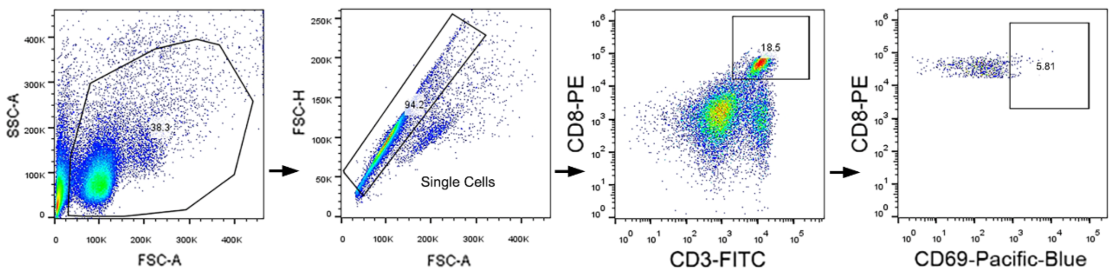

Supplementary Figure 80. Gating strategy to quantify CD69$^+$ cells in CD8 T cells in splenocytes (Supplementary Figure 40b).

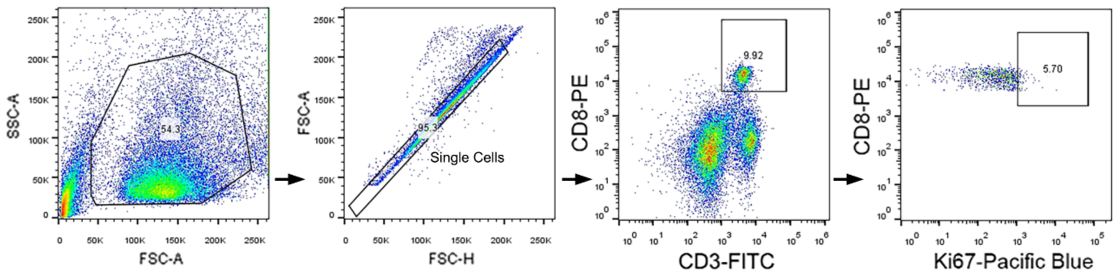

Supplementary Figure 81. Gating strategy to quantify Ki67$^+$ cells in CD8 T cells in splenocytes (Supplementary Figure 40c).

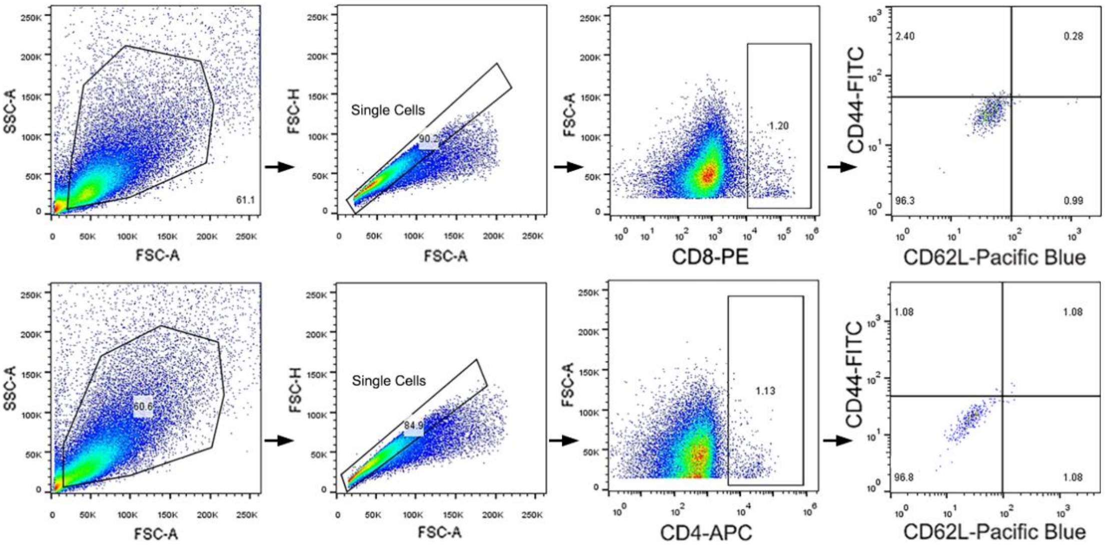

Supplementary Figure 82. Gating strategy to quantify memory T cells in tumors (Supplementary Figure 32).

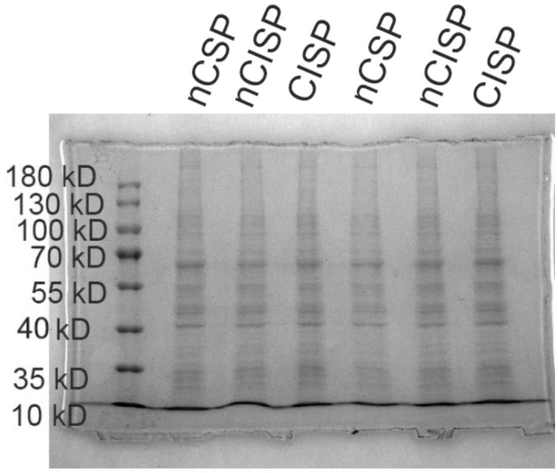

Supplementary Figure 83. Uncropped scans of gel presented in supplementary Figure 4.

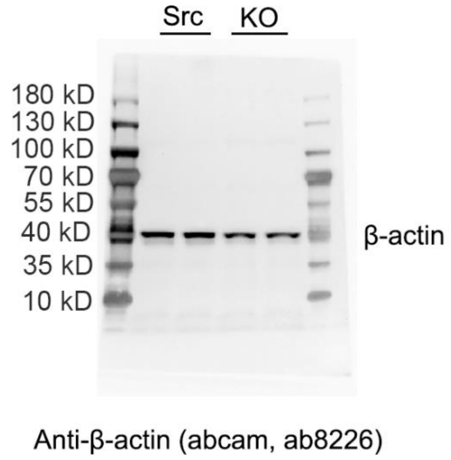

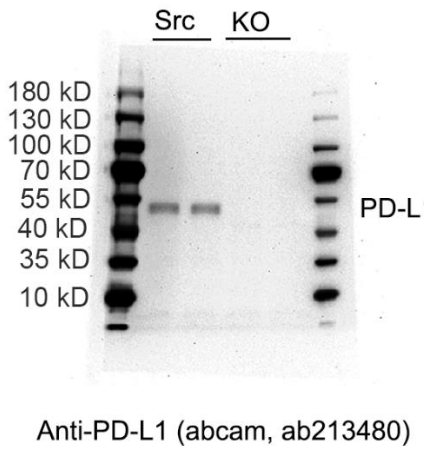

Supplementary Figure 84. Uncropped scans of blots presented in supplementary Figure 7a.

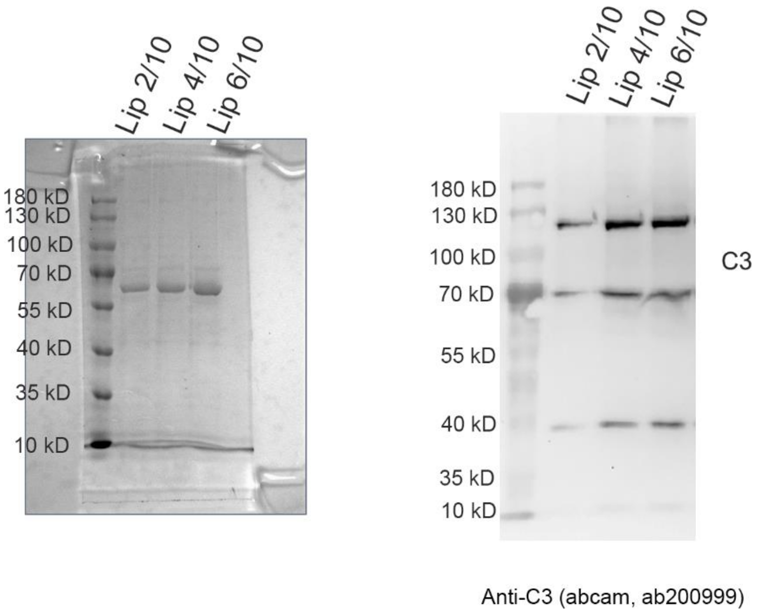

Supplementary Figure 85. Uncropped scans of gel and blot presented in supplementary Figure 27a, b.
