# Supplementary material for: Cholesterol removal improves performance of a model biomimetic system to co-deliver a photothermal agent and a STING agonist for cancer immunotherapy
Source: Nat Commun. 2023 Aug 22;14:5111. doi: 10.1038/s41467-023-40814-9 (PMC10444796; doi:10.1038/s41467-023-40814-9)
Supplement: Supplementary file 3 — Reporting Summary [file 41467_2023_40814_MOESM3_ESM.pdf]

nature portfolio

Corresponding author(s): Ling Zhang and Zhenmi Liu

Last updated by author(s): Aug 3, 2023

Reporting Summary

Nature Portfolio wishes to improve the reproducibility of the work that we publish. This form provides structure for consistency and transparency in reporting. For further information on Nature Portfolio policies, see our Editorial Policies and the Editorial Policy Checklist.

Statistics

For all statistical analyses, confirm that the following items are present in the figure legend, table legend, main text, or Methods section.

n/a	Confirmed	
[ ]	[x]	The exact sample size (*n*) for each experimental group/condition, given as a discrete number and unit of measurement
[ ]	[x]	A statement on whether measurements were taken from distinct samples or whether the same sample was measured repeatedly
[ ]	[x]	The statistical test(s) used AND whether they are one- or two-sided<br>*Only common tests should be described solely by name; describe more complex techniques in the Methods section.*
[x]	[ ]	A description of all covariates tested
[ ]	[x]	A description of any assumptions or corrections, such as tests of normality and adjustment for multiple comparisons
[ ]	[x]	A full description of the statistical parameters including central tendency (e.g. means) or other basic estimates (e.g. regression coefficient) AND variation (e.g. standard deviation) or associated estimates of uncertainty (e.g. confidence intervals)
[ ]	[x]	For null hypothesis testing, the test statistic (e.g. *F*, *t*, *r*) with confidence intervals, effect sizes, degrees of freedom and *P* value noted<br>*Give P values as exact values whenever suitable.*
[x]	[ ]	For Bayesian analysis, information on the choice of priors and Markov chain Monte Carlo settings
[x]	[ ]	For hierarchical and complex designs, identification of the appropriate level for tests and full reporting of outcomes
[x]	[ ]	Estimates of effect sizes (e.g. Cohen's *d*, Pearson's *r*), indicating how they were calculated

Our web collection on statistics for biologists contains articles on many of the points above.

Software and code

Policy information about availability of computer code

Data collection	No stand-alone or custom software was used.
Data analysis	Softwares used in analysis include Flowjo V10, Graph Prism 8, DAS2.0 software

For manuscripts utilizing custom algorithms or software that are central to the research but not yet described in published literature, software must be made available to editors and reviewers. We strongly encourage code deposition in a community repository (e.g. GitHub). See the Nature Portfolio guidelines for submitting code & software for further information.

Data

Policy information about availability of data

All manuscripts must include a data availability statement. This statement should provide the following information, where applicable:

- Accession codes, unique identifiers, or web links for publicly available datasets
- A description of any restrictions on data availability
- For clinical datasets or third party data, please ensure that the statement adheres to our policy

The data that support the findings of this study are available within the paper and its Supplementary Information files. The source data underlying Figure 1d, 1f, 1g, 2b, 2d-g, 3b, 3d-f, 4b, 4c, 4g, 4i, 4k, 4m, 4o, 5b, 5c, 5d, 5f, 5h, 6b-q, 7b-j, Supplementary Figure 2, 3b-d, 4, 5, 6, 7a, 7b, 8a, 9b, 10b, 11b, 13b, 14, 16c, 16e, 17, 18b, 18d, 18f, 19b, 20d, 20f, 20h, 20j, 21b, 22b, 23, 24, 25c, 25e, 25g, 25i, 26, 27a, 27b, 28b, 30a-c, 31a, 31b, 32a-d, 33a, 33b, 35, 36b, 38a-c, 39a-d, 40a-c, 41b, 42a-e, 44, 45b, 45d-n, 47, 48b, 49b, 49e-h, 50a-c, 51a-f, 52a-f, 53a-f are provided as a Source Data file. The Proteomics data generated in this study have been deposited in

the PRIDE under the accession code [https://www.ebi.ac.uk/pride/archive/projects/PXD044151]. Source data are provided with this paper.

Human research participants

Policy information about studies involving human research participants and Sex and Gender in Research.

Reporting on sex and gender	To study the uptake of nCISP and CISP in human blood, blood was obtained from healthy male human (according to local approved protocols and with individual consent. Approved number, Gwll2022117). The experiment was designed without considering the sex of human, and blood of male human was selected to ensure gender uniformity.
Population characteristics	Two healthy males (20-40 years old) were recruited from the community.
Recruitment	Healthy males (20-40 years old) were recruited from the community by posting volunteer recruitment posters in the community. The blood samples were collected in West China Fourth Hospital, and blood was used for incubating with nanoparticles and obtaining monocytes. Considering that the phagocytic activity of phagocytes in the blood may be related to the level of inflammation in the body and may affect the clearance of nanoparticles, the volunteers should have no colds, asthma, arthritis or surgery in the past six months.
Ethics oversight	The collection of blood samples from healthy human was approved Ethics Committee of West China Fourth Hospotal and West China School of Public health, Sichuan University.

Note that full information on the approval of the study protocol must also be provided in the manuscript.

Field-specific reporting

Please select the one below that is the best fit for your research. If you are not sure, read the appropriate sections before making your selection.

☒ Life sciences ☐ Behavioural & social sciences ☐ Ecological, evolutionary & environmental sciences

For a reference copy of the document with all sections, see nature.com/documents/nr-reporting-summary-flat.pdf

Life sciences study design

All studies must disclose on these points even when the disclosure is negative.

Sample size	Sample size were selected to ensure that they are sufficient for statistical comparation between different groups. In addition, the sample sizes of this study were determined on the basis of similar published studies (Nat Nanotechnol. doi:10.1038/s41565-021-00972-7). Details regarding sample size of all experiments are provided in the Methods section and figure legends.
Data exclusions	No data were excluded from the analyses.
Replication	Most in vitro experiments were repeated independently for at least 2 times. All in vivo studies were repeated at least 3 mice, 4 mice per group for analyzing immunes cells, and 5-8 mice per group for monitoring tumor growth and survival periods. The tumor targeting and therapeutic efficacy of CISP were validated in four tumor models. This information is also shown in the figure legends and methods section.
Randomization	Samples were randomly allocated to corresponding experimental groups. Organisms were cultured and maintained in the same environment and randomly allocated to each group.
Blinding	No formal blinding was used in this study. The investigators should keep careful track of experiment protocols because most of experiments needed multiple treatments (e.g. formulations, cells, tumor treatment, and so on). Therefore, it is difficult to blind the investigators to group allocation, drug preparation, drug treatment and data collection. The bioluminescence images were collected by an independent operator, which was unaware of the treatment conditions. The immune cells in tumors were examined by flow cytometry, which was unaware of the treatment conditions.

Behavioural & social sciences study design

All studies must disclose on these points even when the disclosure is negative.

Study description	*Briefly describe the study type including whether data are quantitative, qualitative, or mixed-methods (e.g. qualitative cross-sectional, quantitative experimental, mixed-methods case study).*
Research sample	*State the research sample (e.g. Harvard university undergraduates, villagers in rural India) and provide relevant demographic information (e.g. age, sex) and indicate whether the sample is representative. Provide a rationale for the study sample chosen. For studies involving existing datasets, please describe the dataset and source.*

Sampling strategy	*Describe the sampling procedure (e.g. random, snowball, stratified, convenience). Describe the statistical methods that were used to predetermine sample size OR if no sample-size calculation was performed, describe how sample sizes were chosen and provide a rationale for why these sample sizes are sufficient. For qualitative data, please indicate whether data saturation was considered, and what criteria were used to decide that no further sampling was needed.*
Data collection	*Provide details about the data collection procedure, including the instruments or devices used to record the data (e.g. pen and paper, computer, eye tracker, video or audio equipment) whether anyone was present besides the participant(s) and the researcher, and whether the researcher was blind to experimental condition and/or the study hypothesis during data collection.*
Timing	*Indicate the start and stop dates of data collection. If there is a gap between collection periods, state the dates for each sample cohort.*
Data exclusions	*If no data were excluded from the analyses, state so OR if data were excluded, provide the exact number of exclusions and the rationale behind them, indicating whether exclusion criteria were pre-established.*
Non-participation	*State how many participants dropped out/declined participation and the reason(s) given OR provide response rate OR state that no participants dropped out/declined participation.*
Randomization	*If participants were not allocated into experimental groups, state so OR describe how participants were allocated to groups, and if allocation was not random, describe how covariates were controlled.*

Ecological, evolutionary & environmental sciences study design

All studies must disclose on these points even when the disclosure is negative.

Study description	*Briefly describe the study. For quantitative data include treatment factors and interactions, design structure (e.g. factorial, nested, hierarchical), nature and number of experimental units and replicates.*
Research sample	*Describe the research sample (e.g. a group of tagged Passer domesticus, all Stenocereus thurberi within Organ Pipe Cactus National Monument), and provide a rationale for the sample choice. When relevant, describe the organism taxa, source, sex, age range and any manipulations. State what population the sample is meant to represent when applicable. For studies involving existing datasets, describe the data and its source.*
Sampling strategy	*Note the sampling procedure. Describe the statistical methods that were used to predetermine sample size OR if no sample-size calculation was performed, describe how sample sizes were chosen and provide a rationale for why these sample sizes are sufficient.*
Data collection	*Describe the data collection procedure, including who recorded the data and how.*
Timing and spatial scale	*Indicate the start and stop dates of data collection, noting the frequency and periodicity of sampling and providing a rationale for these choices. If there is a gap between collection periods, state the dates for each sample cohort. Specify the spatial scale from which the data are taken*
Data exclusions	*If no data were excluded from the analyses, state so OR if data were excluded, describe the exclusions and the rationale behind them, indicating whether exclusion criteria were pre-established.*
Reproducibility	*Describe the measures taken to verify the reproducibility of experimental findings. For each experiment, note whether any attempts to repeat the experiment failed OR state that all attempts to repeat the experiment were successful.*
Randomization	*Describe how samples/organisms/participants were allocated into groups. If allocation was not random, describe how covariates were controlled. If this is not relevant to your study, explain why.*
Blinding	*Describe the extent of blinding used during data acquisition and analysis. If blinding was not possible, describe why OR explain why blinding was not relevant to your study.*

Did the study involve field work? ☐ Yes ☐ No

Field work, collection and transport

Field conditions	*Describe the study conditions for field work, providing relevant parameters (e.g. temperature, rainfall).*
Location	*State the location of the sampling or experiment, providing relevant parameters (e.g. latitude and longitude, elevation, water depth).*
Access & import/export	*Describe the efforts you have made to access habitats and to collect and import/export your samples in a responsible manner and in compliance with local, national and international laws, noting any permits that were obtained (give the name of the issuing authority, the date of issue, and any identifying information).*

Disturbance | Describe any disturbance caused by the study and how it was minimized.

Reporting for specific materials, systems and methods

We require information from authors about some types of materials, experimental systems and methods used in many studies. Here, indicate whether each material, system or method listed is relevant to your study. If you are not sure if a list item applies to your research, read the appropriate section before selecting a response.

Materials & experimental systems

n/a	Involved in the study
☐	☒ Antibodies
☐	☒ Eukaryotic cell lines
☒	☐ Palaeontology and archaeology
☐	☒ Animals and other organisms
☒	☐ Clinical data
☒	☐ Dual use research of concern

Methods

n/a	Involved in the study
☒	☐ ChIP-seq
☐	☒ Flow cytometry
☒	☐ MRI-based neuroimaging

Antibodies

Antibodies used

FITC anti-mouse CD3 (catalog number 100204, clone: 17A2, dilution: 1:100), APC anti-mouse CD4 (catalog number 100412, clone: GK1.5, dilution: 1:100), Pacific Blue anti-mouse CD69 (catalog number 104524, clone: H1.2F3, dilution: 1:100), PE anti-mouse CD8a (catalog number 100708, clone: 53-6.7, dilution: 1:200), Brilliant Violet 421 anti-mouse FOXP3 (catalog number 126419, clone: MF-14, dilution: 1:50), PE anti-mouse CD274 (B7-H1, PD-L1) (catalog number 124308, clone: 10F.9G2, dilution: 1:100), FITC anti-mouse/human CD44 (catalog number 103006, clone: IM7, dilution: 1:200), Pacific Blue anti-human/mouse Granzyme B (catalog number 515408, clone: GB11, dilution: 1:20), Pacific Blue anti-mouse Ki-67 (catalog number 652422, clone: 16A8, dilution: 1:100), APC anti-mouse/human CD11b (catalog number 101212, clone: M1/70, dilution: 1:100), PE anti-mouse F4/80 (catalog number 111704, clone: W20065D, dilution: 1:100), Pacific Blue anti-mouse CD80 (catalog number 104724, clone:16-10A1, dilution: 1:200), FITC anti-mouse CD86 (catalog number 105110, clone: PO3, dilution: 1:50), APC anti-mouse NK-1.1 (catalog number 156506, clone: S17016D, dilution: 1:100), APC anti-mouse CD11c (catalog number 117310, clone: N418, dilution: 1:100), FITC anti-mouse CD11c (catalog number 117306, clone: N418, dilution: 1:200), PE anti-mouse CD40 (catalog number 157506, clone: FGK45, dilution: 1:50), FITC anti-mouse CD206 (catalog number 141704, clone: C068C2, dilution: 1:400), Pacific Blue anti-mouse CD62L (catalog number 161208, clone: W18021D, dilution: 1:200), and PE anti-mouse CD279 (PD-1) (catalog number 135206, clone: 29F.1A12, dilution: 1:50) were purchased from Biolegend. Alexa fluor 488 anti-mouse cd274 (PD-L1) was obtain from BD Biosciences (catalog number 568304, clone: 10F.9G2(RUO), dilution: 1:100). Anti-PD-1 (ab214421, clone: EPR20665, dilution: 1:1000), anti-PD-L1 (ab213480, clone: EPR20529, dilution: 1:1000), anti-C3 (ab200999, clone: EPR19394, dilution: 1:1000), anti-TBK1 (ab40676, clone: EP611Y, dilution: 1:5000) and anti-β actin (ab8226, clone: mAbcam 8226, dilution: 1:1000) for western blot were obtained from Abcam. Anti-p-TBK1 (catalog number 5483, dilution: 1:1000) and anti-p-IRF3 (catalog number 29047, dilution: 1:1000) were purchased from Cell Signaling Technology. Anti-IRF3 (catalog number A19717, clone: ARC0198, dilution: 1:1000) was purchased from Abclonal Technology in China. Anti-Na+/K+ ATPase (catalog number ET1609-76, dilution: 1:10000) was purchased from HuaBio in China. PE–conjugated anti-mouse IgG(H+L) secondary antibody (catalog number, SA00008-9, dilution: 1:50), HRP-conjugated Affinipure Goat anti-mouse IgG(H+L) secondary antibody (catalog number, SA00001-1, dilution: 1:8000), HRP-conjugated Affinipure Goat anti-rabbit IgG(H+L) secondary antibody (SA00001-2, dilution: 1:8000), and Anti-LAMP1 (catalog number 67300-1-Ig, dilution: 1:100) was purchased from Proteintech in China.

Validation

FITC anti-mouse CD3 (catalog number 100204, clone: 17A2), (application, FC-Quality tested; verified reactivity, mouse)
https://www.biolegend.com/en-us/products/fitc-anti-mouse-cd3-antibody-45
APC anti-mouse CD4 (catalog number 100412, clone: GK1.5), (application, FC-Quality tested; verified reactivity, mouse)
https://www.biolegend.com/en-us/products/apc-anti-mouse-cd4-antibody-245
Pacific Blue anti-mouse CD69 (catalog number 104524, clone: H1.2F3), (application, FC-Quality tested; verified reactivity, mouse)
https://www.biolegend.com/en-us/products/pacific-blue-anti-mouse-cd69-antibody-4403
PE anti-mouse CD8a (catalog number 100708, clone: 53-6.7), (application, FC-Quality tested; verified reactivity, mouse)
https://www.biolegend.com/en-us/products/pe-anti-mouse-cd8a-antibody-155
Brilliant Violet 421 anti-mouse FOXP3 (catalog number 126419, clone: MF-14), (application, FC-Quality tested; verified reactivity, mouse)
https://www.biolegend.com/en-us/products/pe-anti-mouse-cd8a-antibody-155
PE anti-mouse CD274 (B7-H1, PD-L1) (catalog number 124308, clone: 10F.9G2), (application, FC-Quality tested; verified reactivity, mouse)
https://www.biolegend.com/en-us/products/pe-anti-mouse-cd274-b7-h1-pd-l1-antibody-4497
FITC anti-mouse/human CD44 (catalog number 103006, clone: IM7), (application, FC-Quality tested; verified reactivity, mouse/human)
https://www.biolegend.com/en-us/products/fitc-anti-mouse-human-cd44-antibody-314
Pacific Blue anti-human/mouse Granzyme B (catalog number 515408, clone: GB11), (application, ICFC-Quality tested; verified reactivity, mouse/human)
https://www.biolegend.com/en-us/products/pacific-blue-anti-human-mouse-granzyme-b-antibody-8612
Pacific Blue anti-mouse Ki-67 (catalog number 652422, clone: 16A8), (application, ICFC-Quality tested; verified reactivity, mouse)
https://www.biolegend.com/en-us/products/pacific-blue-anti-mouse-ki-67-antibody-10553

APC anti-mouse/human CD11b (catalog number 101212, clone: M1/70), (application, FC-Quality tested; verified reactivity, Mouse, Human, Cynomolgus, Rhesus)
https://www.biolegend.com/en-us/products/apc-anti-mouse-human-cd11b-antibody-345
PE anti-mouse F4/80 (catalog number 111704, clone: W20065D), (application, FC-Quality tested; verified reactivity, mouse)
https://www.biolegend.com/en-us/products/pe-anti-mouse-f4-80-antibody-22815
Pacific Blue anti-mouse CD80 (catalog number 104724, clone:16-10A1), (application, FC-Quality tested; verified reactivity, mouse)
https://www.biolegend.com/en-us/products/pacific-blue-anti-mouse-cd80-antibody-6267
FITC anti-mouse CD86 (catalog number 105110, clone: PO3), (application, FC-Quality tested; verified reactivity, mouse)
https://www.biolegend.com/en-us/products/fitc-anti-mouse-cd86-antibody-1965
APC anti-mouse NK-1.1 (catalog number 156506, clone: S17016D), (application, FC-Quality tested; verified reactivity, mouse)
https://www.biolegend.com/en-us/products/apc-anti-mouse-nk-11-antibody-19843
APC anti-mouse CD11c (catalog number 117310, clone: N418), (application, FC-Quality tested; verified reactivity, mouse)
https://www.biolegend.com/en-us/products/apc-anti-mouse-cd11c-antibody-1813
FITC anti-mouse CD11c (catalog number 117306, clone: N418), (application, FC-Quality tested; verified reactivity, mouse)
https://www.biolegend.com/en-us/products/fitc-anti-mouse-cd11c-antibody-1815
PE anti-mouse CD40 (catalog number 157506, clone: FGK45), (application, FC-Quality tested; verified reactivity, mouse)
https://www.biolegend.com/en-us/products/pe-anti-mouse-cd40-antibody-19117
FITC anti-mouse CD206 (catalog number 141704, clone: C068C2), (application, ICFC-Quality tested; verified reactivity, mouse)
https://www.biolegend.com/en-us/products/fitc-anti-mouse-cd206-mmr-antibody-7318
Pcific Blue anti-mouse CD62L (catalog number 161208, clone: W18021D), (application, FC-Quality tested; verified reactivity, mouse)
https://www.biolegend.com/en-us/products/pacific-blue-anti-mouse-cd62l-antibody-21800
PE anti-mouse CD279 (PD-1) (catalog number 135206, clone: 29F.1A12), (application, FC-Quality tested; verified reactivity, mouse)
https://www.biolegend.com/en-us/products/pe-anti-mouse-cd279-pd-1-antibody-6170
Alexa fluor 488 anti-mouse cd274 (PD-L1) (catalog number 568304, clone: 10F.9G2(RUO)), (application, FC-Quality tested; verified reactivity, mouse)
https://www.bdbiosciences.com/zh-cn/products/reagents/flow-cytometry-reagents/research-reagents/single-color-antibodies-ruo/alexa-fluor-488-rat-anti-mouse-cd274-pd-l1.568304
Anti-PD-1 (ab214421, clone: EPR20665), (Suitable for: WB, IHC-P, ICC/IF;
Reacts with: Mouse)
https://www.abcam.cn/products/primary-antibodies/pd1-antibody-epr20665-ab214421.html
Anti-PD-L1 (ab213480, clone: EPR20529) [Knockout validated], (Suitable for: ICC/IF, IP, WB; Reacts with: Mouse)
https://www.abcam.cn/products/primary-antibodies/pd-l1-antibody-epr20529-ab213480.html
Anti-C3 (ab200999, clone: EPR19394), (Suitable for: IHC-P, WB; Reacts with: Mouse, Rat, Human)
https://www.abcam.cn/products/primary-antibodies/c3-antibody-epr19394-ab200999.html
Anti-TBK1 (ab40676, clone: EP611Y) [Knockout validated], (Suitable for: ICC/IF, WB, IHC-P; Reacts with: Mouse, Rat, Human)
https://www.abcam.cn/products/primary-antibodies/naktbk1-antibody-ep611y-ab40676.html
Anti-β-actin (ab8226, clone: mAbcam 8226), (Suitable for: ICC/IF, IHC-P, WB; Reacts with: Mouse, Rat, Human)
https://www.abcam.cn/products/primary-antibodies/beta-actin-antibody-mabcam-8226-loading-control-ab8226.html
Anti-p-TBK1 (catalog number 5483), (Suitable for: ICC/IF, WB; Reacts with: Mouse, Human)
https://www.cellsignal.cn/products/primary-antibodies/phospho-tbk1-nak-ser172-d52c2-xp-rabbit-mab/5483?site-search-type=Products&N=4294956287&Ntt=5483&fromPage=plp&_requestid=5747770
Anti-p-IRF3 (catalog number 29047, anti-p-IRF3), (Suitable for: IP, WB; Reacts with: Mouse, Human, Rats)
https://www.cellsignal.cn/products/primary-antibodies/phospho-irf-3-ser396-d6o1m-rabbit-mab/29047?site-search-type=Products&N=4294956287&Ntt=29047&fromPage=plp&_requestid=5813991
Anti-IRF3 (catalog number A19717, clone: ARC0198, dilution: 1:1000) [KO Validated], (Suitable for: WB; Reacts with: Human, Mouse)
https://abclonal.com.cn/catalog/A19717
Anti-Na+/K+ ATPase (catalog number ET1609-76, dilution), (Suitable for: WB, IF, IHC-P; Reacts with: Human, Mouse, Rat)
http://www.huabio.cn/product/Sodium-Potassium-ATPase-antibody-ET1609-76
PE–conjugated anti-mouse IgG(H+L) secondary antibody (catalog number, SA00008-9), (Applications: IF, FC)
https://www.ptgcn.com/products/R-PE-conjugated-AffiniPure-F-ab-2-Fragment-Donkey-Anti-Mouse-IgG-H-L.htm
HRP-conjugated Affinipure Goat anti-mouse IgG(H+L) secondary antibody (catalog number, SA00001-1) (Applications: WB, ELISA)
https://www.ptgcn.com/products/HRP-conjugated-Affinipure-Goat-Anti-Mouse-IgG-H-L-secondary-antibody.htm
HRP-conjugated Affinipure Goat anti-rabbit IgG(H+L) secondary antibody (SA00001-2) (Applications: WB, ELISA)
https://www.ptgcn.com/products/HRP-conjugated-Affinipure-Goat-Anti-Rabbit-IgG-H-L-secondary-antibody.htm
Anti-LAMP1 (catalog number 67300-1-Ig) (Applications: IF, IHC, WB, ELISA; Cited Reactivity : Human, Mouse)
https://www.ptgcn.com/products/CD107a-Antibody-67300-1-Ig.htm

Eukaryotic cell lines

Policy information about cell lines and Sex and Gender in Research

Cell line source(s)

MC38 and B16F10 were brought from American Type Culture Collection and were cultured in a complete RPMI-1640 medium. B16F10 cells transfected with Control Double Nickase Plasmid (Scramble B16F10), and B16F10 cells transfected with PD-L1 Double Nickase Plasmids (B16F10PD-L1 KO cells) were established in our previous work (Acta Biomaterialia. doi:10.1016/j.actbio.2022.10.008) (a kind gift from Prof. Zhirong Zhang, West China School of Pharmacy, Sichuan University, Chengdu, China), and they were cultured in complete RPMI-1640 medium. CTLL2 (catalog number CL-0331) was purchased from Procell Life Science & Tech-nology.Co.,Ltd, and it was cultured in a complete RPMI-1640 medium with IL-2 (100 U mL

	−1). A375 (catalog number CL-0014) and LLC (catalog number CL-0140) were brought from Procell Life Science & Tech-nology.Co.,Ltd, and they were cultured in complete DMEM medium. RAW264.7 (catalog number CL-0190) was brought from Procell Life Science & Tech-nology.Co.,Ltd, and it was cultured in complete RPMI-1640 medium. CTLL2-PD1 was established by Shanghai Genechem in China, and it was cultured in complete RPMI-1640 medium with IL-2 (100 U mL−1). All cells were maintained in a humidified atmosphere incubator containing 5% CO_2 at 37 °C. CD8+ T cells were isolated from spleen of mice.
Authentication	The expression of PD-L1 in B16F10PD-L1 KO cells, the expression of PD-1 in CTLL2-PD1 cells and purity of CD8+ T cells were isolated from spleen were confirmed in this study. No further authentication was conducted on other cell lines.
Mycoplasma contamination	All cell lines were tested negative for Mycoplasm contamination.
Commonly misidentified lines (See ICLAC register)	No commonly misidentified lines were used in this study.

Palaeontology and Archaeology

Specimen provenance	*Provide provenance information for specimens and describe permits that were obtained for the work (including the name of the issuing authority, the date of issue, and any identifying information). Permits should encompass collection and, where applicable, export.*
Specimen deposition	*Indicate where the specimens have been deposited to permit free access by other researchers.*
Dating methods	*If new dates are provided, describe how they were obtained (e.g. collection, storage, sample pretreatment and measurement), where they were obtained (i.e. lab name), the calibration program and the protocol for quality assurance OR state that no new dates are provided.*

☐ Tick this box to confirm that the raw and calibrated dates are available in the paper or in Supplementary Information.

Ethics oversight	*Identify the organization(s) that approved or provided guidance on the study protocol, OR state that no ethical approval or guidance was required and explain why not.*

Note that full information on the approval of the study protocol must also be provided in the manuscript.

Animals and other research organisms

Policy information about studies involving animals; ARRIVE guidelines recommended for reporting animal research, and Sex and Gender in Research

Laboratory animals	Male C57BL/6 mice (18 ± 2 g, 6–8 weeks) were obtained from GemPharmatech in China. Mice were housed in constant environmental conditions (room temperature, 21 ± 1 °C; relative humidity, 40–70% and a 12 h light-dark cycle). Mice were accessed to food and water free.
Wild animals	No wild animals were used in this study.
Reporting on sex	The experiment was designed without considering the sex of mice, and male mice were selected to ensure gender uniformity.
Field-collected samples	No field-collected samples were used in the study.
Ethics oversight	All animal studies were conducted according to the requirements of the national act on the use of experimental animals (China) and in compliance with guidelines evaluated and approved by the Animal Ethics Committee of Sichuan University.

Note that full information on the approval of the study protocol must also be provided in the manuscript.

Clinical data

Policy information about clinical studies

All manuscripts should comply with the ICMJEguidelines for publication of clinical research and a completedCONSORT checklist must be included with all submissions.

Clinical trial registration	*Provide the trial registration number from ClinicalTrials.gov or an equivalent agency.*
Study protocol	*Note where the full trial protocol can be accessed OR if not available, explain why.*
Data collection	*Describe the settings and locales of data collection, noting the time periods of recruitment and data collection.*
Outcomes	*Describe how you pre-defined primary and secondary outcome measures and how you assessed these measures.*

Dual use research of concern

Policy information about dual use research of concern

Hazards

Could the accidental, deliberate or reckless misuse of agents or technologies generated in the work, or the application of information presented in the manuscript, pose a threat to:

No	Yes	
☐	☐	Public health
☐	☐	National security
☐	☐	Crops and/or livestock
☐	☐	Ecosystems
☐	☐	Any other significant area

Experiments of concern

Does the work involve any of these experiments of concern:

No	Yes	
☐	☐	Demonstrate how to render a vaccine ineffective
☐	☐	Confer resistance to therapeutically useful antibiotics or antiviral agents
☐	☐	Enhance the virulence of a pathogen or render a nonpathogen virulent
☐	☐	Increase transmissibility of a pathogen
☐	☐	Alter the host range of a pathogen
☐	☐	Enable evasion of diagnostic/detection modalities
☐	☐	Enable the weaponization of a biological agent or toxin
☐	☐	Any other potentially harmful combination of experiments and agents

ChIP-seq

Data deposition

☐ Confirm that both raw and final processed data have been deposited in a public database such as GEO.

☐ Confirm that you have deposited or provided access to graph files (e.g. BED files) for the called peaks.

Data access links<br>*May remain private before publication.*	*For "Initial submission" or "Revised version" documents, provide reviewer access links. For your "Final submission" document, provide a link to the deposited data.*
Files in database submission	*Provide a list of all files available in the database submission.*
Genome browser session<br>(e.g. UCSC)	*Provide a link to an anonymized genome browser session for "Initial submission" and "Revised version" documents only, to enable peer review. Write "no longer applicable" for "Final submission" documents.*

Methodology

Replicates	*Describe the experimental replicates, specifying number, type and replicate agreement.*
Sequencing depth	*Describe the sequencing depth for each experiment, providing the total number of reads, uniquely mapped reads, length of reads and whether they were paired- or single-end.*
Antibodies	*Describe the antibodies used for the ChIP-seq experiments; as applicable, provide supplier name, catalog number, clone name, and lot number.*
Peak calling parameters	*Specify the command line program and parameters used for read mapping and peak calling, including the ChIP, control and index files used.*
Data quality	*Describe the methods used to ensure data quality in full detail, including how many peaks are at FDR 5% and above 5-fold enrichment.*
Software	*Describe the software used to collect and analyze the ChIP-seq data. For custom code that has been deposited into a community repository, provide accession details.*

Flow Cytometry

Plots

Confirm that:

- [x] The axis labels state the marker and fluorochrome used (e.g. CD4-FITC).
- [x] The axis scales are clearly visible. Include numbers along axes only for bottom left plot of group (a 'group' is an analysis of identical markers).
- [x] All plots are contour plots with outliers or pseudocolor plots.
- [x] A numerical value for number of cells or percentage (with statistics) is provided.

Methodology

Sample preparation	Tumor, tumor-draining lymph node (TdLNs) and spleen were harvested immediately after mice sacrifice. TdLNs were squeezed to filter through a 70 μm cell sieve, cells were resuspended in PBS buffer. Spleens were squeezed to filter through a 70 μm cell sieve, cells were resuspended in ammonium-chloridepotassium (ACK) lysing buffer for 30 min. Tumors were squeezed to filter through a 70 μm cell sieve, cells were resuspended in ACK lysing buffer for 30 min. The obtained cells were stained with the relevant antibodies, and cell were then detected on BD flow cytometer. The obtained flow cytometry data was analyzed by Flowjo V10.
Instrument	BD FACSCelestaTM flow cytometer.
Software	FlowJo V10.
Cell population abundance	The relative abundance was maintained by diluting all the samples at equal volume and collecting samples at a fixed and consistent time.
Gating strategy	A forward/side scatter (FSC/SSC) dot plot was used to gate the main cell population, and the FSC-A/FSC-H dot plot was used to gate the single cell population. Antigen-postive cells were gated according to the cells stained with single antibody. The detail gating strategy used in this study is provided in the Supplementary Information.

- [x] Tick this box to confirm that a figure exemplifying the gating strategy is provided in the Supplementary Information.

Magnetic resonance imaging

Experimental design

Design type	*Indicate task or resting state; event-related or block design.*
Design specifications	*Specify the number of blocks, trials or experimental units per session and/or subject, and specify the length of each trial or block (if trials are blocked) and interval between trials.*
Behavioral performance measures	*State number and/or type of variables recorded (e.g. correct button press, response time) and what statistics were used to establish that the subjects were performing the task as expected (e.g. mean, range, and/or standard deviation across subjects).*

Acquisition

Imaging type(s)	*Specify: functional, structural, diffusion, perfusion.*
Field strength	*Specify in Tesla*
Sequence & imaging parameters	*Specify the pulse sequence type (gradient echo, spin echo, etc.), imaging type (EPI, spiral, etc.), field of view, matrix size, slice thickness, orientation and TE/TR/flip angle.*
Area of acquisition	*State whether a whole brain scan was used OR define the area of acquisition, describing how the region was determined.*

Diffusion MRI ☐ Used ☐ Not used

Preprocessing

Preprocessing software	*Provide detail on software version and revision number and on specific parameters (model/functions, brain extraction, segmentation, smoothing kernel size, etc.).*
Normalization	*If data were normalized/standardized, describe the approach(es): specify linear or non-linear and define image types used for transformation OR indicate that data were not normalized and explain rationale for lack of normalization.*

Normalization template | *Describe the template used for normalization/transformation, specifying subject space or group standardized space (e.g. original Talairach, MNI305, ICBM152) OR indicate that the data were not normalized.*

Noise and artifact removal | *Describe your procedure(s) for artifact and structured noise removal, specifying motion parameters, tissue signals and physiological signals (heart rate, respiration).*

Volume censoring | *Define your software and/or method and criteria for volume censoring, and state the extent of such censoring.*

Statistical modeling & inference

Model type and settings | *Specify type (mass univariate, multivariate, RSA, predictive, etc.) and describe essential details of the model at the first and second levels (e.g. fixed, random or mixed effects; drift or auto-correlation).*

Effect(s) tested | *Define precise effect in terms of the task or stimulus conditions instead of psychological concepts and indicate whether ANOVA or factorial designs were used.*

Specify type of analysis: ☐ Whole brain ☐ ROI-based ☐ Both

Statistic type for inference (See Eklund et al. 2016) | *Specify voxel-wise or cluster-wise and report all relevant parameters for cluster-wise methods.*

Correction | *Describe the type of correction and how it is obtained for multiple comparisons (e.g. FWE, FDR, permutation or Monte Carlo).*

Models & analysis

n/a	Involved in the study
☐	☐ Functional and/or effective connectivity
☐	☐ Graph analysis
☐	☐ Multivariate modeling or predictive analysis

Functional and/or effective connectivity | *Report the measures of dependence used and the model details (e.g. Pearson correlation, partial correlation, mutual information).*

Graph analysis | *Report the dependent variable and connectivity measure, specifying weighted graph or binarized graph, subject- or group-level, and the global and/or node summaries used (e.g. clustering coefficient, efficiency, etc.).*

Multivariate modeling and predictive analysis | *Specify independent variables, features extraction and dimension reduction, model, training and evaluation metrics.*
